# Predicting relationship quality with itself? A single general factor captures most of the variance across 34 common relationship measures

**DOI:** 10.1371/journal.pone.0342451

**Published:** 2026-04-01

**Authors:** James J. Kim, Samantha Joel, Ariana M. Gonzales, Brett A. Murphy, Jacqueline C. Perez, Victor A. Kaufman, Thomas N. Bradbury, Paul W. Eastwick, Benjamin R. Karney

**Affiliations:** 1 Department of Psychology, Lakehead University, Thunder Bay, Ontario, Canada; 2 Department of Psychology, Western University, London, Ontario, Canada; 3 Department of Psychology, University of California, Los Angeles, Los Angeles, California, United States of America; 4 Department of Health and Behavioral Sciences, Texas A&M University-San Antonio, San Antonio, Texas, United States of America; 5 Department of Psychology, University of California, Davis, Davis, California, United States of America; Universiti Sains Malaysia, MALAYSIA

## Abstract

In relationship science, researchers have generated a wide array of constructs and corresponding self-report measures to characterize, explain, and predict *relationship quality* – the foremost studied outcome in the field. Collectively, however, the boundaries among these variables remain unclear. In the current research, we examined the extent to which measures of relationship quality and other important relationship constructs are empirically separable from one another. Across two studies of US census-matched participants (total *N* = 3,439), we applied latent variable techniques (e.g., exploratory bifactor analysis) on broad pools of items representing various prominent relationship-specific constructs. Results revealed robust evidence that a single general factor *Q* (representing global relationship sentiment) accounts for a vast majority of common variance across distinct relationship measures. Thus, respondents appear to draw primarily on their overall global relationship evaluations when reporting on an array of presumably-distinct relationship facets. This is consistent with a ‘sentiment override’ perspective. Our findings provide novel empirical evidence for a relationship-specific response bias that challenges prevailing assumptions and practice in the field, including the widespread use of self-report methods to capture meaningful aspects of relationship functioning.

## Introduction

Emerging scientific disciplines often face a common problem. The popularization of a new field is typically accompanied by an explosion of empirical research, which can quickly outpace the development of organizing theoretical principles. Forscher [1] articulated this issue sixty years ago using a metaphor in which builders (researchers) become so adept at building bricks (findings) that they lose sight of the ultimate goal of constructing edifices (theoretical principles):

The brickmakers became obsessed with the making of bricks. When reminded that the ultimate goal was edifices, not bricks, they replied that, if enough bricks were available, the builders would be able to select what was necessary and still continue to construct edifices. The flaws in this argument were not readily apparent and so, with the help of citizens who were waiting to use the edifices yet to be built… it happened that the land became flooded with bricks.

This metaphor is also apt in the context of subdisciplines within psychology that rely primarily on self-report methods as part of their standard measurement practices [2,3]. When researchers propose a new psychological construct, they frequently develop a new self-report measure to represent it. However, in the absence of coordinated validation efforts to adequately differentiate constructs, this can lead to issues of construct and measure redundancy [3,4]. Indeed, several domains of psychological science have struggled with the proliferation of potentially overlapping constructs and measures in recent years, whether as broad fields (e.g., industrial-organizational psychology; [5]) or as narrower research areas (e.g., empathy; [6], neuroticism; [7]). Studies show that many widely used self-report measures in psychology have been developed with limited evidence of construct validity to support their usage more broadly [8].

The current state of relationship science — the interdisciplinary study of the dynamics, processes, and outcomes of close relationships — is arguably facing a similar issue. A robust body of research links romantic relationship quality to several important life outcomes, including physical health [9], psychological wellbeing [10], stress [11], immune system functioning [12], and satisfaction with life [13]. As such, researchers have made significant efforts over the years and across multiple disciplines (e.g., psychology, sociology, communication, economics, family studies) to identify key correlates and causes of relationship quality. Yet, despite the development of numerous theories, constructs, and measures to study relationship functioning, the accumulation of ostensibly unique relationship variables and assessment tools has unfolded largely without integration [14,15]. Indeed, recent research highlights ongoing conceptual ambiguities regarding key relationship constructs and their empirical relations with relationship quality (e.g., [16]; see [17] for review).

Related to this, relationship research has traditionally relied quite heavily on self-report to capture key phenomena of interest [18]. Yet to date, there have been few systematic investigations assessing the empirical separability of commonly employed relationship measures when assessed using this method. As the number of available constructs and corresponding measures continues to grow, it thus becomes increasingly important that their contributions be examined *collectively,* rather than individually. Similar to other domains of psychological science, such investigations are essential to prevent the piling up of “redundant measures, bifurcated literatures, and constructs without unique psychological importance” [4]. In other words, through critical analysis, we can ensure that each collection of self-report measures adds up to an edifice, rather than a pile of bricks.

In the current article, we conducted a large-scale examination of empirical overlap among prominent constructs within relationship science. Across two studies, we administered a comprehensive set of relationship measures that broadly reflect theory and self-report methods in the field. We then used exploratory bifactor analysis and latent modeling approaches to identify shared and unique sources of variance within the item pool(s). In doing so, our investigation was well-positioned to identify common sources of variance and consolidate diverse self-report items down to a parsimonious collection of empirically distinguishable domains. Such a consolidation effort could have important implications for advancing theory and research practice in the field. For example, findings could help corroborate theoretical frameworks of relationship quality and relationship functioning (e.g., [14,19]) and potentially allow future research to test the potential added value of new relationship constructs or measures without needing to administer an extensive pool of items. More critically, quantifying the empirical overlap among prominent relationship constructs could also inform the degree to which researchers may be engaging in redundant measurement practices, or drawing spurious inferences about associations among relationship variables that are conceptually, but not empirically, distinct.

## Why studying empirical overlap matters in relationship science

Poor construct and measurement clarity remains a pervasive challenge across psychology, and can hinder the theoretical and empirical progress within a discipline. For example, these issues have received particular attention in personality psychology, where construct and measurement proliferation remain major sources of concern (e.g., [20,21]). Most notably, the Big 5 dimensions (and other “Big Few” models) represent a widely influential attempt to create parsimonious coherence out of the “jingle-jangle jungle” [22] of overlapping construct measurements. Researchers commit the “jingle fallacy” [23] when they assume that measures of distinct constructs are identical merely because they have similar labels. The same sources of vagueness also leave researchers susceptible to the “jangle fallacy” [24], wherein researchers assume that measures of identical constructs are different merely because they have different labels. The Big 5 approach, developed through administering large pools of personality items and distilling them into a smaller number of distinct domains (e.g., [25,26]), has since prompted further research to test the distinguishability of related construct measures. For example, in work that bears some conceptual similarity to the approach here, Bainbridge et al. [20] recently conducted two studies where they had participants complete an extensive collection of stand-alone personality scales, as well as a Big 5 inventory, and used exploratory structural equation modeling to test which scales were meaningfully distinguishable from Big 5 measurements; they observed many of these scales offer little incremental validity and can reasonably be labeled as facets of the Big 5.

In relationships research, similar abatement of construct redundancy could be achieved by using latent variable modeling to distill a comprehensive collection of relationship self-report items down to a parsimonious collection of empirically distinguishable domains, potentially creating a “big few” of measurable self-report dimensions in relationship science. Indeed, scholars have noted that a modern challenge for relationship science is to consolidate its theoretical and empirical body of knowledge so that researchers can focus their efforts and resources on identifying central principles and mechanisms responsible for improving relationship outcomes [14]. Yet, there have been few systematic investigations assessing the degree of empirical overlap among the field’s central relationship variables. Below, we highlight a few reasons why relationship research may be particularly affected by the pernicious effects of construct redundancy, including: the large number of relationship variables that currently exist, ongoing conceptual ambiguity surrounding focal relationship constructs, and the reliance on assessing individuals’ subjective evaluations to study relationship phenomena.

### Construct proliferation

Relationship science has long been susceptible to a proliferation of variables. Early inventories developed for research on family relationships initially contained over 700 variables [27], prompting critiques to address the “profusion of variables and propositions in the literature on relationships” [28]. More recently, in a large-scale collaboration, researchers compiled 43 longitudinal datasets of couples from 29 relationship science labs [29] to examine an extensive set of relationship-specific variables (i.e., constructs reflecting judgments about the relationship or the partner) and their influence on relationship quality. A total of 1148 relationship variables with unique labels were identified across studies, which the authors reduced to 62 different relationship-specific constructs through thematic coding. Conceptually similar constructs and measures frequently originate from different theoretical backgrounds and are studied in isolation, leading researchers to assume they function independently. However, these assumptions are rarely adequately tested.

### Fuzzy conceptual boundaries

Relationship scientists have yet to reach consensus about the definition and conceptual boundaries of the primary outcome variable in research on relationships, namely overall relationship quality [30]. We use **relationship quality** as an umbrella term denoting a person’s global, subjective evaluation of whether their relationship is relatively good or bad [19]; other common terms include adjustment (e.g., [31]) or satisfaction (e.g., [32]). These global relationship evaluations are the key outcome that most theoretical models seek to explain and predict in close relationships research (e.g., [14,33]), and serve as the central criterion by which the effectiveness of clinical treatments and programs addressed to couples are measured [34].

For decades, researchers have wrestled with the proper specification and measurement of relationship quality, particularly when deciding which relationship variables fall within versus outside its definition. Yet past attempts to refine its conceptualization and measurement have not addressed the potential circularity of variables considered as predictors or measures of relationship quality. For example, several unidimensional measures of *relationship satisfaction* have been developed over the years and remain widely used, including the Kansas Marital Satisfaction scale (e.g., “How satisfied are you with your marriage?”; [35]), the Relationship Assessment Scale (e.g., “How much do you love your partner?”; [32]), the Quality of Marriage Index (e.g., “My relationship with my partner is very stable”; [36]), and the Couples Satisfaction Index (e.g., “I feel that I can confide in my partner about virtually anything”; [37]). Such measures have conceptualized relationship quality as a global, homogenous dependent variable, thereby implying that by differentiating global evaluations from more specific aspects of the relationship (e.g., communication, conflict, affection), researchers are justified in treating those latter constructs as *predictors* of relationship quality [36,38]. Yet, as evidenced by the example items, such measures appear to subsume a diverse array of relationship aspects. Indeed, one recent examination of 26 relationship quality measures identified 25 different aspects of relationship functioning embedded within the items (e.g., trust, power, forgiveness, sexuality), suggesting that these measures capture highly heterogenous content beyond global evaluations [16]. Overall, the distinctions among relationship quality, its correlates, and its predictors remain vague. Without clearer empirical guidelines, researchers in this area run the risk of attempting to predict relationship quality with itself.

#### Reliance on self-reports and the issue of sentiment override.

Relationship science relies heavily on evaluative self-report assessments of different relationship processes. Although studies in the field frequently involve multi-method approaches (e.g., dyadic, experimental, longitudinal, daily experience), self-report survey methods (e.g., relationship intake surveys) remain prominent. For example, a recent review of 771 independent studies of relationships published in prominent journals between 2014 and 2018 found that self-report data were featured in 96% of them, whereas other types of data collection (e.g., observational or informant ratings) were relatively rare ([18]; see [29] for similar review conclusions). This is unsurprising as most relationship phenomena of interest involve tapping into people’s relationship-specific judgments (e.g., of love, trust, intimacy, perceived partner commitment, etc.).

Reliance on self-reports to study diverse relationship constructs assumes that participants distinguish between different constructs that researchers presume capture distinct features of close relationships. However, there is little direct evidence to support this assumption. Indeed, even when participants are asked to report on specific, concrete relationship behaviors, research indicates such reports are heavily guided by their global evaluations of the relationship (e.g., [39,40]). This phenomenon has been referred to as *sentiment override* [41]; i.e., the tendency for self-reports of specific behaviors to reflect individuals’ overall satisfaction more than particular features of the relationship or partner (e.g., “*How often did my partner hug me this week? I have no idea, but I am quite happy in this relationship, so it must have been a lot.*”). The phenomenon of sentiment override poses a challenge for testing major theoretical propositions within relationship science, insofar as measures of relationship constructs that can be distinguished from relationship satisfaction *in theory* may not be distinguishable *empirically* if the same global sentiments shape responses to both assessments.

Taken together, understanding how intimate relationships succeed or fail requires that researchers incorporate and measure constructs that are clearly distinct from the outcomes they are trying to explain. In the present research, we sought to identify common sources of variance underlying participant self-reports across a comprehensive set of relationship measures to clarify their empirical relations.

## Bifactor modeling as a method for lumping and splitting relationship constructs

To identify the empirical structure underlying relationship science’s multitude of constructs, we employ factor analytic procedures, including exploratory bifactor analysis, which has become instrumental in recent years for researchers seeking to investigate matters of construct-relevant multidimensionality. Bifactor models are especially well-suited for addressing whether it is empirically justified to split research measurements into multiple dimensions, versus lumping them together into a single dimension (e.g., [42,43]). See Rodriguez et al. [44, 45 for an overview of psychometric bifactor indices used to help scholars adjudicate evidence for meaningful multidimensional substance when assessing specific subdomains within item pools. (We provide an overview of the challenges of interpreting general factors in the supplemental materials). In contrast to traditional factor analytic techniques (EFA and CFA), bifactor models decompose the covariance among a collection of indicators (i.e., scale items) into two types of factors: a *general factor* which reflects a single source of common variance among all the indicators, and one or more *specific factors* (orthogonal to the general factor) that explain the remaining unique residual covariance among subsets of indicators. This approach is particularly advantageous when trying to evaluate the empirical distinguishability of multiple overlapping constructs [43,46]. Furthermore, modern applications of exploratory bifactor analysis (EBFA) have been developed and tested over the last decade, providing more robust avenues for mapping the shared and distinctive characteristics across conceptually similar psychological instruments [42,47]. For our present purposes, EBFA is ideal as it allows for evaluating the presence of a general factor to shed light on whether specific relationship judgments may be *principally* driven by overall assessments of relationship quality. In turn, examining specific factors can help inform which relationship constructs are empirically distinguishable and can thus be treated as theoretically distinct predictors of overall relationship quality.

Findings from a bifactor approach can also have implications for evaluating existing relationship theories and frameworks. For example, the Perceived Relationship Quality Components model (PRQC; [19]) remains one of the few direct attempts to empirically delineate the boundaries of relationship quality. Drawing on CFA methods available at the time, this model specifies relationship quality as a higher-order factor model, in which a single, general relationship quality factor is reflected by six *quasi-independent* relationship constructs (satisfaction, commitment, intimacy, trust, passion, and love). Yet, it does not offer clear guidance about whether the overlapping constructs (e.g., love, trust, commitment) should be treated as predictors of relationship quality or facets of it, nor does it clarify whether other (particularly newer) relationship constructs are separate from relationship quality. A bifactor approach partitions the unique covariance accounted for by specific factors from that of a potential general factor, thus providing clearer guidance regarding the incremental validity of measures that purportedly capture distinct relationship constructs [48]. If a general factor explains the vast majority of the variance among constructs, then there would be little empirical justification for considering those constructs separately (e.g., using trust to predict satisfaction “over and above” intimacy would be inappropriate if measures of these constructs are not empirically distinct).

## Overview and hypotheses

In the present work, we explored the empirical relations among a broad range of relationship constructs using a common self-report study design. Across two independent studies, we selected and administered prototypical measures of relationship quality and related relationship constructs to examine the underlying factor structure within these item pools. We aimed to capture a set of measures that would be generally representative of theory and practice in the field. Study 1 focused on relationship satisfaction measures specifically, given that relationship satisfaction is the most common operationalization of relationship quality [49], and research indicating that the large number of satisfaction measures in the literature comprised of highly heterogenous relationship variables [16]. Study 2 included a broader set of relationship measures and constructs, drawing on the most widely measured constructs in the field. We employed multiple factor analytic procedures (primarily EBFA and EFA, but also CFA and CBFA in auxiliary analyses) to ensure that a full range of plausible factor solutions are evaluated [50,51]. To enhance confidence in the stability of our results, several alternative models were additionally tested for convergent evidence; this allowed us to evaluate whether results were robust across different methodological decisions within the factor analytic process (e.g., various rotation/extraction methods).

**Fig 1:**
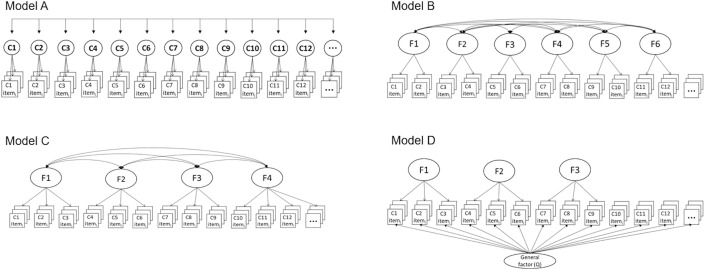
Each model is depicted by a set of relationship constructs, each represented by their respective scale items (depicted via sets of three overlapping squares). Twelve constructs (C1-C12) shown for illustrative purposes. We discuss why one might expect that factor structure to emerge, statistical evidence required to support the model, and the implications of each model for research practices within the field. A) Model A (Independence): Each Relationship Measure Represents a Distinct Construct. Most existing literature assumes that each uniquely-labelled relationship construct is related to—but conceptually independent from—every other construct. The Independence Model would be supported via a factor structure in which representative item sets for each construct load onto separate (correlated) factors. This model would justify the continued practice of measuring and treating these constructs as independent. B) Model B (Corroborative): Relationship Measures Are Theoretically Organized. Relationship measures may cluster into a “big few” of broader, distinct domains with theoretical relevance. Relationship science is built on several prominent theories that may align neatly with such a model [14]. Support for Model B would provide researchers with evidence-based, synthesized frameworks to guide understandings of relationship evaluation and the operationalization of relationship quality. Researchers could develop reliable and valid measures specifically targeting these core dimensions. C) Model C (Unidimensional): Relationship Measures Reflect a Single Construct. Relationship judgments across presumably distinct domains may be explained by a single, overarching factor. This could be interpreted as consistent with the theory of sentiment override [41] in its most assertive form, whereby specific relationship judgments are posited to be primarily driven by an overall assessment of relationship quality (labelled here as ‘Q’). Model C would represent a disconcerting critique of prevailing theories and practices which rest on assumptions that individuals reliably distinguish between distinct aspects of their relationships, which are captured via standard self-report methods. D) Model D (Bifactor): Relationship Measures Have Shared and Unique Features. In Model D, the variance across relationship construct items is explained by both a general factor as well as several group specific factors. Item loadings and bifactor indices (e.g., omegaH, ECV) would inform whether a general factor systematically explains responses across all items (as in Model C), yet meaningful associations also exist among items beyond the influence of a general factor (as in Models A and B). Ultimately, Model D would challenge current practices and theory operating under assumptions of the Independence Model as evidence for a general factor would indicate that current relationship measures ineffectively capture different sources of variance attributable to common and specific relationship constructs.

In light of prior relationship theory, we generated and pre-registered four competing factor models that could plausibly emerge from exploratory factor analyses (i.e., EFA/EBFA). Fig 1 presents these models (Models A-D) with descriptions of what they each suggest about the construal and measurement of relationship quality, as well as their significance for current practice and theory. For each model, we discuss 1) why one might expect that factor structure to emerge, 2) what statistical evidence would be required to support the model, and 3) implications each model would have for research practices within the field.

Each model is depicted by a set of relationship constructs, each represented by their respective scale items (depicted via sets of three overlapping squares). Twelve constructs (C1-C12) shown for illustrative purposes. We discuss why one might expect that factor structure to emerge, statistical evidence required to support the model, and the implications of each model for research practices within the field. **Model A (Independence):** *Each Relationship Measure Represents a Distinct Construct*. Most existing literature assumes that each uniquely-labelled relationship construct is related to—but conceptually independent from—every other construct. The Independence Model would be supported via a factor structure in which representative item sets for each construct load onto separate (correlated) factors. This model would justify the continued practice of measuring and treating these constructs as independent. **Model B (Corroborative)**: *Relationship Measures Are Theoretically Organized.* Relationship measures may cluster into a “big few” of broader, distinct domains with theoretical relevance. Relationship science is built on several prominent theories that may align neatly with such a model [14]. Support for Model B would provide researchers with evidence-based, synthesized frameworks to guide understandings of relationship evaluation and the operationalization of relationship quality. Researchers could develop reliable and valid measures specifically targeting these core dimensions. **Model C (Unidimensional)**: *Relationship Measures Reflect a Single Construct.* Relationship judgments across presumably distinct domains may be explained by a single, overarching factor. This could be interpreted as consistent with the theory of sentiment override [41] in its most assertive form, whereby specific relationship judgments are posited to be primarily driven by an overall assessment of relationship quality (labelled here as ‘Q’). Model C would represent a disconcerting critique of prevailing theories and practices which rest on assumptions that individuals reliably distinguish between distinct aspects of their relationships, which are captured via standard self-report methods. **Model D (Bifactor)**: *Relationship Measures Have Shared and Unique Features*. In Model D, the variance across relationship construct items is explained by both a general factor as well as several group specific factors. Item loadings and bifactor indices (e.g., omegaH, ECV) would inform whether a general factor systematically explains responses across all items (as in Model C), yet meaningful associations also exist among items beyond the influence of a general factor (as in Models A and B). Ultimately, Model D would challenge current practices and theory operating under assumptions of the Independence Model as evidence for a general factor would indicate that current relationship measures ineffectively capture different sources of variance attributable to common and specific relationship constructs.

Broadly, we considered an *Independent Model* (Model A) that reflects existing assumptions regarding the empirical independence of distinct measures of relationship constructs; support for this model would substantiate the use of any given construct to predict another. We considered a *Corroborative Model* (Model B) that reflects the possibility of diverse relationship constructs organizing into a factor structure that corroborates a particular theoretical framework. For example, a factor solution could emerge in which item-level indicators for all relationship constructs are best represented by six first-order factors resembling the six PRQC constructs (i.e., satisfaction, commitment, trust, passion, intimacy, love; [19]). In addition, we considered a *Unidimensional Model* (Model C), whereby relationship evaluations across presumably distinct domains are instead driven by a single, general factor. Finally, we considered a *Bifactor Model* (Model D) in which indicators of relationship construct measures systematically reflect a general relationship quality factor, yet still organize into a number of sufficiently independent relationship constructs (thus, representing a potential integration of Models A, B, or C). Importantly, support for a factor structure other than the *Independence Model* (Model A) would indicate some extent of construct redundancy across measures, ranging from a select few (e.g., Model B) to the full array of relationship constructs (e.g., Model C).

Although the current research goals focused on exploratory factor analytic methods, we also conducted auxiliary analyses specifying confirmatory CFA/CBFA models based on the four pre-registered hypothesized models (Models A-D) to evaluate the evidence in support of each of the model structures; see supplemental materials for details. All EFA and EBFA models were conducted on the full item pool; no items were iteratively removed for the purposes of item reduction as our aim was to characterise the latent dimensionality underlying representative sets of existing relationship measures.

## Study 1

Study 1 used 206 self-report items taken from a set of prominent relationship scales that purport to measure relationship satisfaction and constructs closely related to relationship satisfaction (29 scales and 512 items total; see Table 1). Satisfaction instruments were emphasized given their ubiquitous use as de facto measures of relationship quality (Bradbury et al., 2000). Although many of these instruments are commonly used as measures of relationship satisfaction, inspection of their subscales and item content showed that they tap a wide range of themes (e.g., trust, commitment, perceived partner responsiveness). We deconstructed these original measures, reducing the original 512 items to 206 unique items based on their semantic content (see supplemental materials for additional details). This content coding of the items showed that the 206 items represented 27 ostensibly separate relationship constructs. Our specific interest was in determining whether items representing 27 separate constructs load onto that many factors, or whether an alternative factor structure best describes the data. Given the exploratory nature of this study, we conducted EFA and EBFA to identify latent dimensions that may account for the shared variance among observed variables. EBFA estimated the proportion of reliable variance among the items accounted for by a general factor, and the proportion accounted for by one or more specific factors. All data, code, and materials used in this research are available on OSF: https://osf.io/e452p.

**Table 1:** Sources, constructs, and number of retained items from satisfaction scales used in Study 1.

Scale	Source	Constructs/Subscales Coded	# Items	Citation #
Appreciation in Relationships (AIR)	Gordon et al. (2012)	Appreciation by the Partner, Appreciation for the Partner	9	312
Barrett-Lennard Relationship Inventory (BLRT)	Barrett-Lennard (1962)	Appreciation by the Partner, Expressing Affection, Perceived Partner Regard, Support, Trust	9	911
Commitment Inventory	Stanley & Markman (1992)	Own Commitment	2	1,055
Communal Strength Scale	Mills et al. (2004)	Communal Strength	4	315
Communication Patterns Questionnaire (CPQ)	Christensen & Sullaway (1984)	Being Understood, Communication	12	522
Couples Satisfaction Index (CSI-16)	Funk & Rogge (2007)	Global Positive Sentiment, Perceived Partner Satisfaction	4	1,702
Dyadic Adjustment Scale (DAS)	Spanier (1976)	Divorce Proneness, Global Negative Sentiment, Perceived Similarity, Shared Activities	7	10,915
Dyadic Trust Scale	Larzelere & Huston (1980)	Critique of the Partner, Trust	4	2,005
ENRICH Marital Satisfaction Scale (EMS)	Fowers & Olson (1993)	Communication, Idealistic Distortion, Perceived Similarity	4	665
Frequency and Acceptability of Partner Behavior	Doss & Christensen (2006)	Critique of the Partner, Expressing Affection, Support, Trust	5	33
Friendship Network Satisfaction Scale	Kaufman et al. (2021)	Being Understood, Communication, Own Commitment, Shared Activities, Support	9	4
Braiker-Kelley Partnership Questionnaire	Braiker & Kelley (1979)	Communication, Conflict, Global Negative Sentiment, Own Commitment, Perceived Partner Commitment	7	1,194
Index of Sexual Satisfaction (ISS)	Hudson et al. (1981)	Sex	2	546
Investment Model Scale	Rusbult et al. (1998)	Global Positive Sentiment, Investment, Own Commitment, Perceived Partner Commitment	11	2,661
Kansas Marital Satisfaction Scale (KMS)	Schumm et al. (1986)	Global Positive Sentiment	1	1,143
Marital Instability Scale	Booth et al. (1983)	Divorce Proneness	4	530
Marital Problems Scale	Amato & Rogers (1997)	Critique of the Partner	7	1,149
Marital Satisfaction Inventory (MSI)	Snyder (1979)	Communication, Conflict, Future Expectations, Global Negative Sentiment, Perceived Similarity	5	686
Marital Satisfaction Scale (MSS)	Blum & Mehrabian (1999)	Communication, Conflict, Critique of the Partner, Expressing Affection, Global Negative Sentiment, Global Positive Sentiment, Perceived Partner Satisfaction, Perceived Similarity, Sex, Shared Activities, Trust	21	353
Marital Satisfaction Scale (MSS)	Roach et al. (1981)	Admiration for the Partner, Communication, Critique of the Partner, Future Expectations, Global Negative Sentiment, Global Positive Sentiment, Perceived Partner Regard, Role Fulfillment, Shared Activities, Trust	21	472
Perceived Partner Responsiveness Scale (PPR)	Reis et al. (2017)	Being Understood, Empathy, Shared Activities	5	67
Perceived Relationship Quality Components (PRQC)	Fletcher et al. (2000)	Admiration for the Partner, Global Positive Sentiment, Perceived Partner Satisfaction, Perceived Partner Commitment, Sex, Trust	6	1,069
Personal Assessment of Intimacy in Relationships (PAIR)	Schaefer & Olson (1981)	Communication, Critique of the Partner, Empathy, Global Negative Sentiment, Idealistic Distortion, Perceived Similarity, Sex, Shared Activities, Socializing with Friends	18	1,246
Positive and Negative Semantic Differential (PN-SMD)	Mattson et al. (2013)	Global Negative Sentiment, Global Positive Sentiment	4	150
Quality of Marriage Index (QMI)	Norton (1983)	Global Positive Sentiment	3	2,332
Quality of Sex Inventory (QSI)	Shaw & Rogge (2016)	Sexual Satisfaction	6	41
Relationship Assessment Scale (RAS)	Hendrick (1988)	Global Negative Sentiment, Global Positive Sentiment	2	2,779
Relationship Satisfaction Scale (RS)	Roysamb et al. (2014)	Being Understood, Conflict, Divorce Proneness, Global Positive Sentiment, Perceived Similarity	5	50
Single item	Park et al. (2019)	Appreciation by the Partner	1	38
Face-valid	--	Admiration for the Partner, Perceived Partner Satisfaction, Perceived Partner Commitment, Sex, Shared Activities, Socializing with Friends, Support	8	--

*Notes. Scales, sources, constructs, and number of retained items from each of the original scales, as well as citation counts at the time the research was conducted. F*or space considerations, we provide references for scales in the supplemental materials.

### Method

#### Participants and procedure.

Items were administered in an online survey distributed via Dynata research platform in January 2021. Participants consisted of a US census-matched, national panel sample of individuals in romantic relationships, with demographic characteristics proportionate to the U.S. Census [52]. Participant demographic information is provided in Table S1 in S1 File and S2 File in the supplemental materials. Written informed consent was obtained from all participants prior to their involvement in completing the questionnaires. Only participants who indicated that they currently had a main romantic involvement and were between the ages of 18 and 75 were eligible. Among qualifying participants who received the survey invitation, 3,001 completed all survey questions (with no missing data). Data integrity measures included five attention checks, inserted randomly throughout the survey; participants who failed any of the engagement checks were excluded. This resulted in a final sample of 2,000 respondents. Survey completion took approximately 20 minutes. Respondents were compensated with cash, rewards points, or discounts. All procedures were approved by the University of California, Los Angeles’ Institutional Review Board.

### Measures

A literature review by four research team members was conducted targeting measures of relationship satisfaction and its proximal correlates; this process identified 29 distinct scales with previous usage in the literature (see Table 1, and section 4 in supplemental materials). Notably, all measures ask respondents to make an evaluative rating on some aspect of their relationship, and many of these instruments contained multiple subscales. Measured constructs ranged from relationship satisfaction itself to constructs presumed to account for variance in satisfaction (e.g., trust, partner responsiveness, conflict). The initial item set comprised 512 total items representing 27 different content areas. Given the high degree of item-content overlap, this item set was consolidated further by: eliminating duplicate items; generalizing wording with equivalent meaning (e.g., spouse versus wife/husband versus partner); separating double-barreled items; removing technical language and jargon; and rephrasing questions to statements to ensure commensurate responding on the same response scale. The final item set contained 206 unique items that included multiple items assessing each of the original 27 content domains. We provide a complete list of the 206 items and their scale sources in S4 Table in S1 File of the supplement. Items were presented in random order, with participants rating their agreement with each item on a 6-point scale from 0 (Not at all Agree) to 5 (Completely Agree).

### Analyses

#### Exploratory factor analysis.

We used the *psych* package [53] in R [54] to conduct a series of exploratory analyses. We examined factor solutions based on common EFA selection criteria guidelines [50,51]. This included screeplots, hierarchical item clustering, Horn’s parallel analysis, Velicer’s minimum average partials (MAP), Bayesian Information Criterion (BIC), and sample size adjusted BIC (SABIC). Relationship constructs were expected to be correlated with one another; thus EFA models were estimated using oblique rotations (promax) given its efficiency with larger datasets [55]. We examined all candidate factor solutions suggested across the various selection methods, conducting separate analyses for each potential n-factor solution derived from EFA metrics with three different extraction methods (maximum likelihood, minimum residual, principal axis). We determined the final number of factors based on EFA metrics and factor solution interpretability. We employed lenient lower-end thresholds for statistical criteria to allow maximal opportunities for higher factor solutions to be retained (primary factor loadings ≥ .30 and cross-loadings ≤ .30 on other factors).

#### Exploratory bifactor analysis.

When the final solutions from EFA models include multiple correlated factors, it is appropriate to evaluate whether the multiple factors are distinct, reliable facets or whether all items are informed by one general underlying dimension [44,56]. When there is possible multidimensionality, as was the case here, EBFA is useful for evaluating the incremental benefit, if any, of scoring subdomains represented by specific factors, or whether a total score (representing a general factor) is sufficient to account for the variability in a set of items [44,56].

A key feature of the bifactor model is the estimation of several psychometric indices that inform the presence and strength of general and specific factors, including omega hierarchical (omegaH: *ω*_H_), omega hierarchical subscale (omegaHS: *ω*_HS_), and explained common variance coefficients (ECV, ECV_SS_) [44,57]. OmegaH (*ω*_H_) reflects the proportion of variance in a unit-weighted total score attributable to a general factor. Higher omegaH (e.g., *ω*_H_ > .80; [44]) reflects the reliable variance in unit-weighted composite scores primarily attributable to a single latent construct, an indicator that items are essentially unidimensional [45]. Although there is no clear consensus on strict cutoffs for bifactor indices, prior research suggests *ω*_H_ values should be at least.50, and optimally.75 or.80 to signify acceptable reliability [44,58]. *ω*_HS_ estimates the proportion of reliable systematic variance remaining in group factors after partialing out variance associated with the general factor. Previous research suggests *ω*_HS_ values of.50 as a reasonable minimum for interpreting that a subset of items coheres as a unique dimension that is independent of the general factor [59,60]. Further, ECV is an index of unidimensionality that estimates the proportion of total variance explained by the general factor alone, where factors are assumed to be uncorrelated. Scale scores are perfectly unidimensional when all common variance is due to a general factor; as ECV approaches zero, scores reflect multiple uncorrelated dimensions. A high ECV value (e.g., > .70; [45]) lends support for a strong general factor and unidimensionality of a scale’s items. ECV_SS_ indices can be computed for specific factors to estimate the explained common variance for items loading on a specific factor. Dueber and Toland [57] have suggested a ECV_SS_ value ≥ .30 as sufficient to interpret a specific factor subscore in the context of confirmatory models.

Overall, we evaluated *ω*_H_ and ECV values, whereby high values would indicate the presence of a prominent general factor underlying the set of indicators, and low values would indicate potential evidence of multidimensionality. We also evaluated *ω*_HS_ and ECV_SS_ values to assess whether any specific factors identified representing distinct subdomains could be interpreted alongside a general factor. Low values would indicate that a specific factor does not explain sufficient variance beyond the influence of the general factor to be considered a meaningful subdomain. Given a range of different cutoffs for bifactor indices in the literature, we erred on interpreting support for multidimensionality more generously, to align with prevailing assumptions and practices in the field. To date, there are no fixed sample size guidelines for EBFA, and simulation studies suggest that sample size adequacy depends on model features rather than a single heuristic [61]. However, we note that our final samples (Study 1: N = 2,000; Study 2: N = 1,439) are above levels Bader et al. [61] found sufficient in most scenarios (i.e., ≥ 500), and meet or exceed thresholds used in simulation-based accurate recovery of exploratory bifactor structures (e.g., [62–64]).

EBFA was conducted using the *fungible* (Waller, 2021) and *psych* (Revelle, 2021) packages in R (R Core Team, 2022), drawing on growing EBFA resources available to researchers (e.g., [43,62,63]). At present, more work is needed to definitively establish which rotational methods are optimal across different analytic contexts. However, in recovering complex bifactor structures consisting of items cross-loading across multiple specific factors and/or items loading strongly on the general factor but not appreciably on any specific factor (i.e., *pure indicators* of the general factor; [62,65,66]), studies suggest that Schmid-Leiman with iterative target rotation (SLiD) modestly outperforms other methods in accurately recovering factor structures [62, 63, 67]. We expected our data would likely take on a more complex rather than simple bifactor structure as it would be reasonable to anticipate several ‘pure indicators’ [44] of a general relationship quality factor emerging from this item pool. Thus, we applied SLiD and report these results throughout this manuscript. However, as a robustness measure, we re-ran all analyses using five other bifactor algorithms to see whether results convergent and ensure that our conclusions were not simply a by-product of the estimation method (see Table S3 in S1 File supplemental materials). The *BifactorIndicesCalculator* package in R [68] was used to extract *omegaH* and *ECV* reliability estimates*.*

### Study 1 results

#### Exploratory Factor Analyses (EFA).

Results suggested a wide range of different potential factor solutions across selection criteria, specifically models consisting of 1, 3, 4, 12, 13, 17, and 20 correlated factors. We examined each of these factor solutions across the different extraction methods (see Table S3 File in supplement). Results were highly consistent across extraction methods; here, we report results from maximum likelihood estimation. A one-factor model accounted for 45% of the variance with absolute factor loadings (|λ|) ranging from.09 to.92 (*M* = .65). A two-factor model accounted for 56% of the variance with 133 items loading onto a factor capturing all positively phrased items (|λ| = .50 to.87) and 73 items loading onto a factor capturing all negatively phrased items (|λ| = .42 to.83) (−.48 correlation between factors). A three-factor model accounted for 59% of the variance with 125 items loading onto a Positive factor (|λ| = .50 to.87), 72 items loading onto a Negative factor (|λ| = .47 to.82), and 9 items about Sex loading onto a separate factor (|λ| = .48 to.63) with factor correlations ranging from.25 to.48. Items on the Sex factor had cross-loadings above.30 with other factors. A four-factor model accounted for 60% of the variance with 125 items loading onto a positive factor (|λ| = .43 to.88), 71 items onto a negative factor (|λ| = .45 to.80), 11 items onto a factor about sex (|λ| = .45 to.70) and no items loading more strongly onto Factor 4, with factor correlations ranging from.01 to.53.Results from models with four or more factors indicated that factors were likely over-extracted given that no items loaded distinctly on the fourth factor, and the gains in explained variance were small. Thus, these results supported three correlated factors as the best characterization of the data. The top 10 loading items for each of the three factors in the EFA model are presented in Table 2 (see Appendix C for full pattern matrix).

**Table 2:** Study 1 Top 10 Item Factor Loadings in 3-factor EFA Model (206 items).

Item	F1	F2	F3
I feel very lucky to have my partner in my life.	**0.89**	0.07	−0.10
I am committed to maintaining my relationship with my partner.	**0.89**	0.07	−0.20
I want our relationship to last a very long time.	**0.88**	0.04	−0.21
My partner is one of the best people I know.	**0.87**	0.11	−0.11
My relationship with my partner is strong.	**0.87**	0.11	−0.04
I feel very attached to my partner.	**0.87**	0.02	−0.09
I really feel like part of a team with my partner.	**0.86**	0.05	−0.01
I want to grow old with my partner.	**0.86**	0.12	−0.22
The future of my relationship with my partner looks promising to me.	**0.86**	0.12	−0.06
All things considered, I am very happy in my relationship with my partner.	**0.86**	0.09	0.03
My partner and I often discuss or consider separation or ending our relationship.	−0.05	**0.82**	−0.15
My relationship with my partner is miserable.	0.11	**0.78**	−0.07
My partner makes unfair demands of my free time.	−0.05	**0.78**	−0.05
When we have problems, my partner and I threaten one another with negative consequences.	−0.08	**0.78**	−0.09
I often feel angry or resentful toward my partner.	0.07	**0.77**	0.04
During a discussion of a relationship issue or problem, my partner and I blame, accuse, and criticize one another.	0.04	**0.77**	0.00
I often consider ending my relationship with my partner.	0.13	**0.77**	−0.14
I feel that my partner disapproves of me.	0.03	**0.76**	0.02
When we have problems, my partner pushes, shoves, slaps, hits, or kicks me.	−0.19	**0.76**	−0.13
My partner gets me badly flustered and jittery.	0.02	**0.75**	−0.03
My partner thinks our relationship is in trouble.	0.01	**0.75**	−0.07
My sex life with my partner is very exciting.	0.34	−0.18	**0.63**
My sex life with my partner is fulfilling.	0.42	−0.08	**0.63**
My partner enjoys our sex life.	0.38	−0.10	**0.59**
Sex is fun for my partner and I.	0.44	−0.08	**0.58**
My partner is willing to try new things in bed.	0.34	−0.15	**0.56**
I am satisfied with our sexual relationship.	0.46	−0.06	**0.54**
My partner seems disinterested in sex.	−0.15	0.44	**0.53**
My partner is very sensitive to my sexual needs and desires.	0.41	−0.10	**0.49**
I am able to tell my partner when I want sexual intercourse.	0.41	−0.08	**0.47**
	Factor Correlations		
F1	–		
F2	−.48	–	
F3	.45	−.23	–

*Note.* Factor loadings in bold indicate the strongest loading items for each factor. See Appendix C for full pattern matrix.

When examining individual item loadings, we see that many items commonly treated as indicators of distinct relationships constructs loaded onto the same factors. For example, the first factor appeared to capture global, positive evaluations of the relationship, as represented by items like “I am satisfied with my partner” and “Our relationship is strong” loaded strongly (>.80); yet, it was also strongly represented by items designed to capture constructs that are often considered antecedents or consequences of partners’ positive evaluations. For example:

• Commitment: “I am committed to maintaining my relationship with my partner” (.89)
• Understanding: “My partner understands me” (.82)
• Trust: “I can always trust my partner” (.80)
• Communal strength: “Meeting the needs of my partner is a high priority for me” (.77)

A similar pattern emerged for the second factor. Items indicative of poor relationship quality (e.g., “My relationship with my partner is miserable,”) loaded strongly onto this factor but not the first factor, suggesting respondents distinguished negative evaluations of their relationships from positive ones. Yet, within this factor, items designed to assess distinct constructs considered related to relationship dissatisfaction loaded about as strongly as the negative evaluations themselves. For example:

• Divorce proneness: “My partner and I often discuss or consider divorce, separation, or terminating our relationship.” (.82)
• Hostile conflict: “My partner and I blame, accuse, and criticize one another” (.77)
• Perceived partner criticism: “I feel that my partner disapproves of me” (.76)
• Intimate partner violence: “When we have problems, my partner pushes, shoves, slaps, hits, or kicks me” (.76)

Only the third factor captured a specific content domain that respondents treated as distinct from global positive and global negative evaluations of the relationship: satisfaction with sex. Nine of the 206 items loaded strongly onto this factor (e.g., “My sex life is fulfilling,” λ = .63). Items assessing sexual enjoyment (“Sex is fun for my partner and me,” λ = .59), variety (“My partner is willing to try new things in bed”, λ = .56), and communication (“I am able to tell my partner when I want sexual intercourse”, λ = .48) also loaded onto this factor; these all had cross-loadings above.30 with other factors.

Overall, EFA results found that the 27 relationship constructs did not organize into 27 factors, but rather three. When examined together, the sole distinctions in participants’ responses pertained to items related to positive evaluations, negative evaluations, and sex-related evaluations.

#### Exploratory Bifactor Analyses (EBFA).

Although EFAs identified three correlated factors, this does not mean unequivocally that the three factors themselves are distinct enough from one another to merit being measured separately. It is possible that the items loading onto these factors might demonstrate more complex or idiosyncratic patterns of covariance than can be effectively captured through conventional EFA approaches. To address that question, an exploratory bifactor model was specified with a general factor and three specific factors. We present the top 10 loading items per factor in Table 3 (see Appendix C for all factor loadings).

**Table 3:** Study 1 EBFA Top 10 Item Factor Loadings and Bifactor Indices.

	Bifactor Model with 3 specific factors			
Item	General (Q)	SF1	SF2	SF3
My partner understands me.	**.89**	−.12	.04	.08
I am very happy about how we make decisions and resolve conflicts.	**.88**	−.06	.05	.09
I know I’m valued and appreciated by my partner.	**.88**	−.10	.04	.05
My relationship with my partner is enjoyable.	**.88**	−.21	.02	−.02
All things considered, I am very happy in my relationship with my partner.	**.88**	−.28	.00	−.02
My relationship with my partner is strong.	**.88**	−.24	−.02	.09
My partner makes me feel special.	**.88**	−.05	.11	−.07
My relationship with my partner is rewarding.	**.87**	−.23	.01	.01
My partner expresses gratitude towards me often.	**.87**	.03	.08	.00
During a discussion of a relationship issue or problem, my partner and I feel understood by each other.	**.86**	−.11	.03	.07
I want our relationship to last a very long time.	.68	**.55**	.02	.01
I want to grow old with my partner.	.71	**.54**	.09	.00
I want this relationship to stay strong no matter what rough times we may encounter.	.68	**.53**	.06	.00
I love my partner.	.69	**.52**	.04	.08
I am committed to maintaining my relationship with my partner.	.72	**.52**	.03	−.01
I would go out of my way to do something for my partner.	.45	**.48**	.05	.00
I would not feel very upset if my relationship with my partner were to end in the near future.	.64	**.47**	−.06	.02
My life would seem empty without my relationship to my partner.	.63	**.45**	.00	−.04
It is hard to imagine my life without my partner.	.58	**.44**	.00	−.01
I feel very attached to my partner.	.75	**.42**	−.03	.03
My partner and I often discuss or consider separation or ending our relationship.	.31	−.12	**.70**	.01
When we have problems, my partner pushes, shoves, slaps, hits, or kicks me.	.15	−.08	**.67**	−.01
I often consider ending my relationship with my partner.	.45	−.23	**.66**	−.04
When we have problems, I push, shove, slap, hit, or kick my partner.	.06	−.05	**.66**	−.01
When we have problems, my partner and I threaten one another with negative consequences.	.32	−.01	**.66**	.04
My relationship with my partner is miserable.	.49	−.16	**.66**	−.05
My partner makes unfair demands of my free time.	.38	.02	**.65**	.04
When we have problems, I call my partner names, swear at them, or attack their character.	.20	−.01	**.64**	.03
My partner is too flirtatious with other men/women.	.26	−.01	**.63**	.08
My partner thinks our relationship is in trouble.	.39	−.06	**.63**	.00
Sex is fun for my partner and I.	.69	.08	−.17	**.56**
My sex life with my partner is fulfilling.	.71	−.01	−.18	**.52**
My partner seems disinterested in sex.	.40	−.11	.31	**.51**
My sex life with my partner is very exciting.	.59	−.05	−.26	**.50**
My partner enjoys our sex life.	.65	−.01	−.19	**.49**
My partner is willing to try new things in bed.	.56	.00	−.22	**.48**
I am satisfied with our sexual relationship.	.72	.03	−.16	**.46**
I am able to tell my partner when I want sexual intercourse.	.61	.07	−.15	**.43**
My partner and I are sexually compatible.	.64	.15	−.13	**.42**
My partner is very sensitive to my sexual needs and desires.	.62	.00	−.18	**.38**
	Bifactor Indices			
	*ω*_H_	*ω*_HS_	*ω*_HS_	*ω*_HS_
	.92	.03	.05	.00
	ECV	ECV_SS_	ECV_SS_	ECV_SS_
	.73	.06	.18	.03

*Note.* Factor loadings in bold indicate the strongest loading items for each general and specific factor. SF = specific factor; *ω*_H_ = omega hierarchical; *ω*_HS_ = omega hierarchical subscale; ECV = Explained Common Variance; ECV_SS_ = Explained Common Variance of Specific Factor.

First, the item loadings indicated that the general factor, which we refer to as “Q”, was best reflected by items conveying positive global evaluations about one’s relationship and/or partner (i.e., relationship quality). Several of these items belong to measures of independent relationship constructs, however. For example:

• Perceived partner responsiveness: “My partner understands me.” (.89)
• Satisfaction: “All things considered, I am very happy in my relationship with my partner.” (.88)
• Perceived partner appreciation: “I know I’m valued and appreciated by my partner.” (.88)

**Fig 2:**
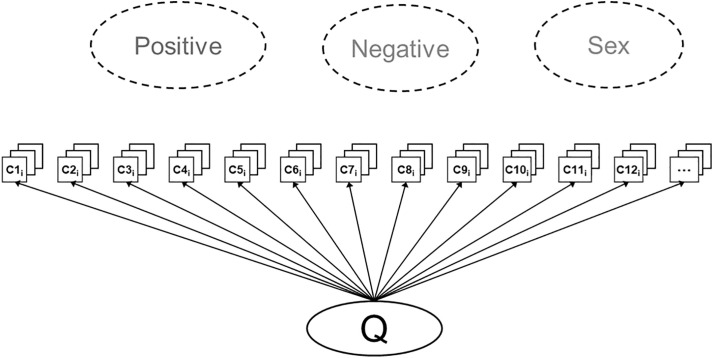
Bifactor model from Study 1 EBFA. Results indicate a strong general factor (Q) explaining the variance across all items. The three specific factors examined (depicted in dotted circles) did not receive sufficient support to warrant interpretation (i.e., did not account for meaningful variance once accounting for **Q)**.

In turn, the three specific factors aligned with the Positive, Negative, and Sex factors identified in the EFA. However, this Positive factor was more closely aligned with indicators of emotional attachment (e.g., “I want our relationship to last a very long time”), and almost no items loaded more strongly on this factor than on the general factor. Further, all items loaded positively on Q, regardless of whether they were positively or negatively-keyed, and stronger loading items were more evaluative and general in content (e.g., “I am satisfied with my partner”) rather than concrete and specific in content (e.g., “My partner and I have very few friends in common”). OmegaH (*ω*_H_) was.92, exceeding thresholds for interpreting a latent general factor, and OmegaHS (*ω*_HS_) for the three specific factor scores were.03 for emotional attachment,.05 for negativity, and.00 for sex, well below thresholds for interpreting meaningful variance being captured by the group factor scores over and above the influence of a general factor (e.g., *ω*_HS_ = .50; [60]). Lastly, ECV was 73%, indicating that nearly three-fourths of the common variance among the 206 items was explained by the general factor alone; ECV_SS_ for the specific factors were 6%, 18%, and 3%, respectively. Thus, despite representing 27 separate content domains across numerous relationship satisfaction scales, the 206 items administered here mostly reflected a single underlying dimension, with little unique variance remaining explained by the specific group factors (see Fig 2).

### Study 1 discussion

Study 1 results revealed a single general factor underlying all items, and no evidence for any other incrementally valid specific relationship factor after accounting for the general factor. This provided initial empirical evidence of construct and measurement redundancy: when self-reporting on diverse relationship satisfaction measures, responses appear to be governed by a single, global evaluation of the relationship rather than by drawing on several distinct relationship constructs. This response pattern is consistent with the process of sentiment override [41]; alternatively, it could reflect a generalized response bias [69]. Overall, Study 1 examined a comprehensive set of individual items with distinct meanings on their face, but found that such meanings did not cohere into separate constructs once the general evaluative stance was accounted for.

### Study 2: Replication and extension

Study 2 aimed to replicate and expand the scope of Study 1’s findings, while addressing limitations and alternative explanations. Specifically, we examined a wider range and number of items representing a more comprehensive set of relationship constructs. This larger item set included the same 206 items from Study 1 (to allow for a direct replication test), and 202 additional items to examine empirical overlap among measures of representative relationship constructs more broadly.

Study 2’s design and hypotheses were pre-registered. The methodological framework closely paralleled that of Study 1, with some modifications. One limitation of Study 1 was that some constructs were represented by a low number of (prototypical) items (e.g., only a few items were coded as indicators of trust from established trust measures). Thus, we included a more balanced number of item indicators per construct, and from prominent scales commonly used to measure said construct (see Table S6 in S1 File supplemental materials). This strengthened the content validity of constructs represented in the item pool, and ensured at least three items per construct [70]. To address concerns that the general factor observed in Study 1 represents a response bias driven by low effort responding [71], we recruited participants through a different recruitment platform (CloudResearch), which research suggests provides higher data quality [72]. We also adopted more stringent data screening measures (e.g., improved attention checks, participant attentiveness index; [73]). Given that the general Q-factor might reflect a generalized response bias not specific to romantic relationship judgments, Study 2 additionally included non-relationship items to capture general well-being (GWB) and generalized evaluative consistency bias (ECB: the tendency to systematically rate oneself more positively or negatively than is warranted across characteristics, regardless of content) [69]. ECB is related to other response biases such as halo [74–76], positivity or acquiescence bias [77], and socially desirable responding (e.g., [78]). Our ECB items were evaluative in nature but not directed towards one’s romantic relationship (e.g., “I have good athletic ability”). Thus, we tested whether Q is a construct that is specific to relationship evaluation by examining its associations with general response bias and general well-being factors.

#### Study 2 exploratory hypotheses.

Given the expanded scope of our design, we did not have firm hypotheses regarding whether we would find equally strong evidence for a general factor (Q) as in Study 1, or the number of specific factors that may emerge. However, we reasoned that our design provided a better chance for specific factors to emerge given the broader range of constructs included. For instance, models of evaluative processing in relationships suggest cognitive representations of a partner can be differentiated based on global evaluations and specific perceptions [79]. Study 2 included several prominent relationship constructs that were not represented in the satisfaction-focused items in Study 1 (e.g., partner-specific attachment, capitalization, self-disclosure), many of which reflect relationship appraisals at lower and more specific levels of abstraction. Hierarchical trait models would also suggest that these constructs are less inherently evaluative compared to more abstract, global appraisals [80]. Overall, Study 2 included an ostensibly more diverse item pool, thus increasing the likelihood that distinct item sets form specific factors separate from Q.

### Method

#### Participants and procedure.

In July 2022, an online panel sample of 2,371 US participants was recruited and compensated through CloudResearch Prime Panels. As in Study 1, participants were 18+ and currently in a romantic relationship, and we used quota sampling based on census data [52] to obtain a final sample with demographic characteristics approximating the US population on several demographic characteristics (i.e., age, gender, race/ethnicity, income). Written informed consent was obtained from all participants prior to their involvement in completing the questionnaires. All procedures were approved by Western University’s Research Ethics Board.

Study 2 consisted of 408 items from different relationship measures, plus additional general well-being and evaluative consistency bias items. Given the large number of variables, we implemented a planned missingness design [81] to reduce study length and respondent fatigue. Specifically, the survey used a Random Percentage design set at 50% missingness: this rate was selected based on research demonstrating negligible effects on model parameter estimation and data quality at our sample size [82]. All participants provided demographic information (e.g., age, gender, race/ethnicity, income) and responded to a common item set that measured general well-being and evaluative consistency bias. As part of the planned missingness design, each participant then responded to 204 relationship items randomly selected from the pool of 408 (i.e., 50%).

We excluded participants from the final sample if they indicated they were not in an established relationship (e.g., single, casually dating) and/or failed any attention checks included in the survey. We also screened and excluded participants for straightlining (i.e., responding identically to item blocks) and speeding responses (i.e., responding more than 40% faster than the median response time; [83]). Further, we computed a participant attentiveness index for every participant following the squared discrepancy procedure by Litman et al. [73] to identify and remove inattentive participants (see section 11 in supplemental materials). The final sample consisted of 1,439 respondents. The survey took approximately 24 minutes. As in Study 1, participants rated their agreement with each item from 1 = *Strongly disagree* to 5 = *Strongly agree*.

### Measures

#### Broad set of relationship-specific items.

A review of relationship measures in the field was conducted by members of the research team to select 408 prototypical items representing 34 of the most prominent relationship constructs. These constructs were selected based on research identifying the most frequently studied constructs in relationship science (Table 2 in [29]). Item indicators for each construct were selected from established measures based on their prevalence in the literature (i.e., selecting measures with the highest citation count), and based on the length of the measure (i.e., favoring shorter versus longer measures). Items were also selected based on their ability to represent a wider range of constructs beyond the 206 items retained from Study 1 (see section 5 in supplemental materials). Appendix A provides a complete list of items and their sources. Appendix B provides a list of the constructs and the number of indicators per construct in Study 2.

Overall, this selection process resulted in an item pool broadly representative of theory and practice in relationship science. Items reflected a comprehensive array of self-report measures used to capture central aspects of relationship functioning, including variables represented across major relationship theories (e.g., *interdependence theory*: satisfaction, quality of alternatives; *attachment theory*: partner-specific anxious and avoidant attachment; *investment model*: satisfaction, commitment, *communal/exchange theory*: communal strength; *triangular theory of love*: passion, intimacy; *vulnerability-stress-adaptation model*: conflict, *ideal standards model*: perceived partner traits) [14].

#### Evaluative consistency bias and general well-being items.

Study 1 identified a general factor which presumably reflects a global evaluation of relationship quality. To investigate how dissociable this general factor is from a (non-relationship specific) response tendency (e.g., halo bias; [75]), we included additional items designed to measure evaluative consistency bias (ECB). This response bias can be considered a type of method artifact characterized by a consistent pattern of responding to items, regardless of the actual content of those items [76]. ECB items consisted of self-evaluations that were unrelated to the romantic relationship (i.e., “*I am a physically attractive person*”*,* “*I have good athletic ability”*, “*I consider myself to be intelligent”, “My general trivia knowledge is excellent*”; [74]). General well-being (GWB) items captured non-relationship specific assessments of well-being (i.e., “*Overall, I am satisfied with my life*”, “*My mental health is very good*”, “*I am doing well in my professional life*”, “*I have a good social life*”).

### Analyses

Pre-registered exploratory analyses were conducted on two, separate, predefined subsets of items within the data. First, we performed EFA and EBFA on the original 206 items from Study 1, as a direct replication test. We repeated this procedure on the broader item pool by examining the 408 relationship-specific items representing 34 different constructs. Given the planned missingness design, multiple imputation was used to account for missing data as it provides unbiased estimates and standard errors when data are MCAR [84]. We used the *mifa* package in R to impute incomplete data for all items used in analyses [85] following the fully conditional specification approach [86] with predictive mean matching (PMM) method [87]. Missing values were imputed five times; resulting covariance matrices of the imputed data sets were combined into a single covariance matrix using Rubin’s method [88].

To assess whether the expected general Q-factor represents a relationship-specific construct, we examined correlations between Q and latent ECB and GWB factors following a latent variable approach similar to Anusic et al. [74]. Latent Q was specified with eight pure indicator items of the general factor (i.e., items that load appreciably onto Q but not any other potential specific factors) identified from EBFA results (see Table 5). We reasoned that if Q is a response pattern guided by global relationship-specific evaluations (consistent with sentiment override), then its representative items should reflect broad evaluations of one’s relationship or partner. In addition, Q should be moderately, but not substantially, correlated with ECB and GWB. However, if Q is correlated substantially with ECB or GWB, it would counter the notion that Q represents relationship-specific sentiment override.

**Table 4:** Study 2 EBFA Model Top Factor Loadings and Bifactor Indices.

	Bifactor Model (Q + 3 specific factors)			
Item	Q	SF1	SF2	SF3
My ideas and wishes are often ignored by partner.	**−.79**	.10	.06	.10
My partner respects me.	**.77**	.02	−.21	−.01
I know I’m valued and appreciated by my partner.	**.75**	.00	−.19	−.01
I have a warm and comfortable relationship with my partner.	**.76**	.01	−.13	.07
My partner feels affection for me.	**.78**	.05	−.12	.10
My partner makes sure I feel appreciated.	**.74**	.08	−.11	−.06
All things considered, I am very happy in my relationship with my partner.	**.75**	−.04	−.09	.08
My partner does not feel affection for me.	**−.75**	.01	.06	.01
My relationship with my partner is rewarding.	**.74**	−.01	−.09	.14
My relationship with my partner is enjoyable.	**.74**	−.01	−.03	.13
I would enjoy having authority over my partner.	−.17	**.58**	−0.09	0.00
I like to have power over my partner.	−.17	**.57**	−0.17	−0.03
I work to control my partner more than they control me.	−.20	**.53**	−0.17	−0.04
I have a strong drive to get power in my romantic relationship.	−.01	**.53**	−0.04	0.11
I try to have more influence than my partner.	−.21	**.51**	−0.19	−0.02
I like to tell my partner what they should do.	−.12	**.49**	−0.14	0.04
I think I have a great deal of power in my romantic relationship.	.33	**.40**	−0.14	−0.04
When I tell my partner about something good that has happened to me, my partner reminds me that most good things have their bad aspects as well.	−.28	**.37**	0.04	0.05
In our relationship, my partner often uses force (like hits, holds me down, or uses a weapon) to make me have sex.	−.20	**.37**	0.01	−0.08
In our relationship, I often use force (like hitting, holding down, or using a weapon) to make my partner have sex.	−.12	**.35**	−0.03	−0.12
My sex life with my partner is very exciting.	.58	0.20	**.46**	0.00
Sexual activity with my partner is fantastic.	.61	0.19	**.43**	0.05
I am happy with my sex life with my partner.	.64	0.13	**.42**	−0.02
My sex life with my partner is fulfilling.	.61	0.14	**.40**	0.06
Sex is fun for my partner and I.	.63	0.12	**.40**	0.02
Sexual activity with my partner is rewarding.	.60	0.10	**.37**	0.13
My relationship is sexually intense.	.36	0.23	**.36**	0.02
I am satisfied with our sexual relationship.	.59	0.13	**.36**	−0.06
My partner and I are sexually compatible.	.53	0.05	**.34**	0.11
My partner is sexy.	.50	0.05	**.33**	0.24
I love my partner.	.51	−0.11	0.04	**.39**
I want my relationship with my partner to last forever.	.54	−0.13	0.01	**.38**
I’m afraid my partner may abandon me.	−.42	0.23	0.24	**.36**
I want my partner physically, emotionally, mentally.	.52	−0.01	0.09	**.36**
I would do almost anything for my partner.	.45	−0.07	0.01	**.35**
It is hard to imagine my life without my partner.	.53	−0.08	0.03	**.35**
I want this relationship to stay strong no matter what rough times we may encounter.	.51	−0.11	−0.04	**.34**
I want our relationship to last a very long time.	.57	−0.12	−0.03	**.34**
I care about my partner.	.46	−0.10	−0.02	**.34**
I would be willing to give up a lot to benefit my partner.	.29	−0.03	0.01	**.33**
	Bifactor Indices			
	*ω*_H_	*ω*_HS_	*ω*_HS_	*ω*_HS_
	.69	.12	.00	.16
	ECV	ECV_SS_	ECV_SS_	ECV_SS_
	.82	.07	.06	.05

*Note.* Factor loadings in bold designate the strongest loading items for each general and specific factor. SF = specific factor; *ω*_H_ = omega hierarchical; *ω*_HS_ = omega hierarchical subscale; ECV = Explained Common Variance; ECV_SS_ = Explained Common Variance of Specific Factor.

**Table 5:** Item labels and standardized factor loadings for Study 2 measurement model.

Latent Variable	Indicator	Item	Loading
	Q1	All things considered, I am very happy in my relationship with my partner.	**.77**
	Q2	My partner is responsive to my needs.	**.74**
	Q3	I am satisfied with my partner.	**.75**
Q	Q4	My partner meets my needs.	**.74**
	Q5	Our relationship makes my partner very happy.	**.69**
	Q6	My relationship with my partner is close to ideal.	**.67**
	Q7	My partner is very loving and affectionate.	**.71**
	Q8	My partner often tells me s/he loves me.	**.70**
	Athletic	I have good athletic ability.	**.61**
ECB	Attractive	I am a physically attractive person.	**.60**
	Trivia	My general trivia knowledge is excellent.	**.42**
	Intelligent	I consider myself to be intelligent.	**.53**
	Overall Life	Overall, I am satisfied with my life.	**.73**
GWB	Mental Health	My mental health is very good.	**.66**
	Professional Life	I am doing well in my professional life.	**.61**
	Social Life	I have a good social life.	**.68**

*Note*. Variable information corresponds to the specified model in Fig 3.

### Study 2 results

#### Direct replication of study 1.

We replicated the results from Study 1; EBFA results showed robust support for a general Q-factor in the 206 items based on two-, three-, and four-factor EFA candidate solutions (see section 6 in supplemental materials). Examination of top-loading items from EBFA for general and specific factors yielded consistent findings, indicating that Q is best represented by evaluations of relationship satisfaction and perceptions of a partner’s regard (e.g., perceived partner responsiveness). Specific factors were only interpretable up to the first 3 specific factors and represented as follows: specific factor 1 = negative evaluations (i.e., conflict items), specific factor 2 = positive evaluations reflecting emotional attachment (i.e., love and commitment items), and specific factor 3 = satisfaction with sex.

#### Expanding to 408 relationship items.

Similar to Study 1, EFA metrics suggested a range of candidate factor solutions (i.e., 1, 2, 3, 4, 6, 11, 15, 16, and 17 factors). Following similar procedures, we again found that the best support for a three-factor model, despite the broader range of item content. The three-factor model was represented by a positive factor (capturing positive global partner evaluations), a negative factor (reflecting variables such as desire for relational power and intimate partner violence), and a sexual satisfaction factor (see S8 Table in S1 File for item loadings).

Based on EFA results, EBFA models were examined for two, three, and four specific factors. Results were consistent with Study 1 findings. Inspection of item loadings for the general factor again showed that Q was best represented by items conveying positive global evaluations about one’s relationship and/or partner. For example:

• Perceived Partner Affection: “My partner feels affection for me.” (.78)
• Perceived Partner Appreciation: “My partner respects me.” (.77)
• Satisfaction: “I have a warm and comfortable relationship with my partner.” (.76)

Examination of specific factors found that these were only interpretable up to the first three specific factors; thus, this solution was retained for subsequent analyses. These factors resembled the three factors identified in previous results. The first specific factor was characterized by negative evaluations, such as items tapping into desire for power over partner (e.g., “I would enjoy having authority over my partner.”, λ = .58) and intimate partner violence. The second specific factor was characterized almost exclusively by sexual satisfaction items, with items loading moderately on this factor (e.g., “I am happy with my sex life with my partner.”, λ = .42). The third specific factor was characterized by broader (positive) items representing emotional attachment (e.g., “I’m afraid my partner may abandon me”, λ = .36). Notably, however, these items had relatively low loadings (<.40). The top loading items in this EBFA model are presented in Table 4 (see Appendix D for all factor loadings, and section 7 in supplemental materials for additional details).

Results of bifactor indices converged with prior findings, showing that a general factor (Q) accounted for most of the variance across items, and minimal evidence to support the substantiveness of any unique specific factors. OmegaH (.69) and ECV (.82) values for Q remained high, while OmegaHS values for specific factor scores (.16,.12,.00) were well below cutoffs for interpretation of subdomains. ECV_SS_ indices (.07,.06,.05) were below cutoffs as well, indicating that only 5–7% of the common variance among subsets of items for each of the specific subdomains could be explained by a specific latent factor.

#### Probing the content of Q.

**Fig 3:**
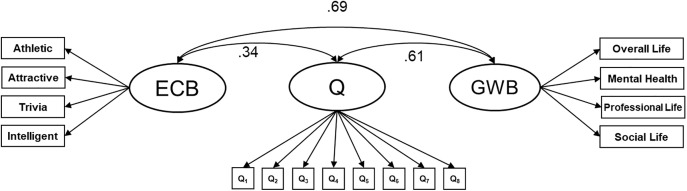
Study 2 model examining associations between Q and non-relationship specific items. ECB = Evaluative Consistency Bias; GWB = General Well-Being from Study 1 EBFA. Q was specified with eight pure indicator items of the general factor identified from Study 2 EBFA (see Table 5). Factor correlations between Q, ECB, and GWB are shown.

Based on our evaluation of the item content, Q represent global relationship evaluations, consistent with sentiment override. To probe this further, we specified a confirmatory model to examine intercorrelations between Q, ECB, and GWB (see Fig 3 for the specified model, and Table 5 for measurement model parameters). This model exhibited satisfactory fit, with items loading well onto their specific factors (CFI = .93, TLI = .92, SRMR = .06, RMSEA = .05). Inter-factor correlations revealed a moderate correlation between Q and ECB (.34), suggesting Q has similar features of a more general systematic response tendency, despite being conceptually distinct. Q was highly correlated with GWB (.61), indicating a robust link between subjective appraisals of general and relationship-specific well-being. Further, ECB was highly correlated with GWB (i.e.,.69), suggesting a stronger influence of a generalized response bias on appraisals of general well-being than relationship quality.

ECB = Evaluative Consistency Bias; GWB = General Well-Being from Study 1 EBFA. Q was specified with eight pure indicator items of the general factor identified from Study 2 EBFA (see Table 5). Factor correlations between Q, ECB, and GWB are shown.

#### Auxiliary analyses.

We conducted auxiliary analyses in which we modeled a latent method factor using CFA and BCFA to further probe construct-irrelevant substantive variance attributable to evaluative consistency bias. A series of competing confirmatory models were specified and compared, with similar results, suggesting Q has similar features of a more general systematic response tendency (ECB), but is not redundant with this factor (see S11 Table in S1 File). We also conducted auxiliary confirmatory analyses to evaluate the evidence in support of each of the model four pre-registered hypothesized model structures (i.e., Models A-D); see section 12 in supplemental materials. Further, at a reviewer’s request, we re-ran all analyses including cases previously excluded from data screening measures (see section 15 in supplementary materials). In short, results were largely consistent; however, factor recovery was notably poorer. EFA/EBFA solutions were less coherent and interpretable compared to the screened sample. This pattern is consistent with the impact of including lower-quality respondents in factor analytic models [89].

### Study 2 discussion

Study 2 replicated the results of Study 1 with an independent sample and within a larger and broader pool of items representing 34 prominent constructs. Results showed no meaningful variance captured by specific factors over and above the influence of a prominent general factor (representing global evaluations of the relationship). The content of these potential specific factors was consistent with Study 1, reflecting negative evaluations, sexual satisfaction, and emotional attachment. As in Study 1, bifactor indices showed weak evidence for specific relationship factors beyond Q, preventing interpretation of these factors. Importantly, findings did not support a ‘multidimensional model’ (Independence Model: Model A), as there was little evidence of any multidimensional solution that clearly represented the 408 items. Instead, EBFA showed that a global relationship quality factor (Q) explained over 80% of the covariance across items. Q was best represented by items capturing (positive) general relationship and partner evaluations rather than specific evaluations, consistent with a sentiment override account [41,90]. Further analyses to probe the content of Q suggested it was modestly linked with, yet distinct from, evaluative consistency bias and general well-being.

## General discussion

A central goal of relationship science is to explain and predict relationship quality. To this end, researchers typically administer various relationship self-report measures, each of which is presumed to capture meaningful yet distinct aspects of an individual’s relationship. However, the distinctions among these measures and the constructs they purport to capture have been left murky. Although scholars have developed numerous relationship constructs and self-report scales designed to capture unique and meaningful aspects of relationship functioning, these efforts have been spread out across disciplines and in an uncoordinated manner. As a result, there remains a lack of consensus regarding the conceptual and empirical boundaries of focal relationship constructs, and ambiguity over which phenomena should be treated as facets versus predictors of relationship quality. Fincham and Bradbury [38] highlighted these concerns nearly four decades ago, noting that the same content often appeared on self-report measures of satisfaction as well as self-report measures of variables like communication and conflict used to predict relationship satisfaction. To date, however, there have been few concerted efforts to explore the empirical relations between overlapping relationship variables and identify potential redundancies within an ever-growing brickyard of relationship measures.

In the current paper, we systematically tested the empirical separability of a broad range of relationship-specific constructs using standard data collection methods in relationship research (i.e., self-report). In two studies, we employed factor analytic methods, including EBFA, to assess the common and distinct sources of variance underlying a comprehensive set of self-report measures representing prominent relationship constructs in the field. We placed particular emphasis on measures related to relationship quality, a construct that has remained conceptually and methodologically elusive despite its broad interdisciplinary importance [16]. In Study 1, we selected measures representing a diverse array of constructs found within measures of relationship quality. In Study 2, we extended our focus and included additional measures of prominent constructs related to relationship quality and reflecting common theories and practices in the field. As part of our investigation, we generated competing hypotheses about how the content within the item pools would organize based on prior theory and prevailing assumptions regarding the independence of these measures that guide current practice. We incorporated pre-registered study designs and analyses to ensure our key findings were replicable and robust.

### Key findings: The influence of sentiment override in relationship self-reports

A central finding from our investigation is that items from commonly used relationship measures of ostensibly different constructs showed extensive empirical overlap. Thus, most measured relationship constructs appear to be “jangles” [3] of global appraisals of relationship quality. Exploratory bifactor models showed that a general relationship quality factor (Q) captured the vast majority of the variance (>70% across studies) among items from distinct relationship constructs and measures. In Study 1, despite ostensibly tapping into 27 different relationship constructs (e.g., appreciation, commitment, trust, communication, responsiveness), most of the common variance in the diverse relationship quality items (73%) could be attributed to a single, general factor (i.e., Q). Study 2 replicated these findings with a similar sample, using more stringent screening criteria to assess the generalizability and content of Q. Again, despite ostensibly tapping into 34 prominent constructs (e.g., intimacy, love, quality of alternatives, communal strength), the vast majority (82%) of the common variance in these items could be explained by Q, which was best indicated by general relationship appraisals. Accordingly, there was little validity evidence of additional unique subdomains beyond Q; although consistent patterns emerged with respect to the content of three domains (i.e., positive, negative, sex), these patterns were weak and did not receive sufficient empirical evidence to warrant interpretation. Still, while short of factor-level interpretation, the general patterns observed were broadly consistent with prior literature. For example, items suggesting a possible ‘negative’ specific factor aligns with work describing positive and negative dimensions of relationship functioning as independent rather than opposite poles of a single continuum [91–92]. Items suggesting a unique “sex” factor is consistent with prior theory that conceptualizes sexual evaluations as related to, yet distinct from, other relationship evaluations [93], as well as the field-level treatment of relationship functioning and sexuality as distinct research foci (e.g., dedicated conferences and journals for close-relationships and sexuality research). Items suggesting a possible “emotional attachment” factor likewise coincide with theoretical perspectives on human bonding. For example, attachment theory treats attachment processes as a core motivational system and attachment bonds as emotionally significant in ways that may not be fully captured by global appraisals of relationship quality [14]. Aside from these broad themes, however, these data revealed none of the finer theoretical distinctions that relationship scientists have drawn between different ways of evaluating a relationship; there was insufficient justification to consider three factors separately, much less the dozens of constructs combined within those factors.

With respect to our hypothesized models, we interpret these findings as strong evidence against the Independence Model, which reflects prevailing assumptions in the field that existing self-report scales of purportedly distinct constructs are empirically distinct. Rather, our results suggest that each of these scales—and perhaps most or even all self-report scales intended to capture relationship-specific evaluations—may have more in common than previously thought as they primarily tap into a single, overarching construct. Subsequent analyses suggested that the general factor (Q) is driven, in part, by a response bias that appears to be most consistent with the phenomenon of *sentiment override* [41,94]. That is, individuals appear to draw on their global evaluations of one’s partner or relationship even when responding to items meant to capture specific relationship features or events. Inspection of item loadings revealed that Q was strongly represented by items that are highly evaluative and reflect general positive appraisals of the relationship or a partner’s regard (e.g., “My relationship with my partner is enjoyable”; “My partner feels affection for me”). Thus, when a researcher attempts to use commitment, love, investment, or trust to predict relationship quality, they may be inadvertently using a person’s global feelings about their relationship to predict their global feelings about their relationship (i.e., attempting to predict relationship quality with itself).

To our knowledge, our findings are among the first in the literature to lend direct empirical support for relationship sentiment override. As sentiment override was originally articulated in the context of observational research assessing partners’ communication and response behaviors during marital interactions [90,94], its significance has become less pronounced within the content of contemporary self-report practices in the field. However, our findings indicate that it warrants far more scholarly attention than it has received to date. For example, if many established associations in relationship science are in fact reflections of sentiment override, then it matters a great deal whether this occurs as a result of demand characteristics (i.e., partners provide consistent reports across instruments because they believe that researchers expect consistency) or whether it truly reflects a lack of specificity in the way partners evaluate their relationships. More critically, the strong presence of sentiment override identified in our examination of representative relationship variables raises the concern that researchers may be at risk of drawing meaningful associations between different self-report measures that are exaggerated or spurious. This presents significant impediments for theoretical and empirical advancements in the field.

### Theoretical implications: Testing theories in light of measurement redundancy

The current findings have significant implications for testing and developing theories in the relationships literature. First, it is important to note that these implications hinge on the distinction between constructs and measures. Indeed, most theoretical formulations in psychology are about constructs, not measures. For example, the investment model [95] posits that investment—the time and resources that a person has placed into a relationship—shapes a person’s feelings of commitment, or their intention to remain in the relationship long-term. This theoretical model requires that investment is a conceptually distinct phenomenon from commitment. It does not maintain, however, that existing *measures* of those constructs are empirically distinct. Our finding that investment and commitment items load onto the same general relationship quality factor suggests that existing investment measures may not be distinguishable from commitment measures, and further that neither one of these may be distinguishable from satisfaction measures. This does not necessarily mean that the constructs of investment and commitment do not exist in principle. Rather, it may indicate that we have not yet managed to distinguish between them with self-report scales.

Nevertheless, a hallmark of scientific theories is their ability to be falsified through empirical testing and capacity to inform real-world phenomena. Although the veracity of a theory does not rest on whether its features can be captured empirically, its verifiability does. Our findings do not directly refute the ‘nomological validity’ of relationship science’s constructs—the extent to which a construct’s measure behaves as it should with other constructs in theoretically predicted ways [96]. Yet, to study links between theoretically meaningful constructs, it is imperative that their measures are empirically separable. If researchers are to effectively study how conflict shapes relationship quality, for example, it is necessary to ensure that measures of conflict and relationship quality capture separate constructs.

We had hoped our examination would produce (or reaffirm) a handful of differentiable “big few” constructs that could be used as building blocks by future relationship theorists to better explain, predict, and improve relationship quality. However, this goal was not realized, at least not as intended. Instead, a takeaway from the current research is that sentiment override is a major looming concern in relationship research, influencing participant responding whenever research calls upon partners to evaluate any aspect of each other and their relationship. The fact that individuals did not appear to discriminate between different relationship-specific constructs through self-report is not a direct threat to existing relationship theories per se; however, it does pose a major obstacle for researchers wishing to test said theories through sole reliance on self-report methods. Our data revealed none of the finer theoretical distinctions that relationship researchers have drawn between different ways of evaluating a relationship, indicating that until better tools and methodological approaches are developed and adopted, researchers will be compromised in their ability to test theoretical models comprised of such constructs.

### Practical implications: Improving measurement practices in relationship research

The current research provides compelling reasons for scholars to broaden their perspectives when considering factors contributing to success or failure in intimate relationships. Our findings indicate that most measures of evaluative relationship constructs are likely capturing global appraisals of relationship quality. By ignoring this concern, researchers run the risk of proposing new constructs and measures which may already exist in the literature. Notably, our focus here was on variables and research practices in relationship science. However, issues of measurement proliferation and construct redundancy are relevant across psychological subdisciplines [97]. Several fields have wrestled with issues of empirically overlapping variables and disentangling potentially spurious sources of variance (e.g., positive manifold: [98]; ‘crud’ factor: [99]; method biases: [77]). Yet, these issues have yet to receive sufficient attention in the relationship literature, despite concerns of conceptual clarity surrounding focal constructs like relationship quality, and a methodological reliance on self-reports to study relationship phenomena [17]. We outline several key considerations for addressing issues of construct redundancy and improving empirical research practices in the field.

First, our findings reveal that existing methods may fail to differentiate between relationship constructs as they are typically measured. The strong presence of sentiment override evidenced in our studies poses a substantial threat for researchers relying on self-report data to draw inferences about the predictive and incremental validity of relationship constructs. This highlights a greater need for more rigorous standards and “riskier” tests of discriminant validity to be adopted, in which researchers are incentivized to disconfirm the uniqueness of new or established measures. Indeed, the importance of shared method variance has been widely known among researchers for establishing convergent and discriminant validity in questionnaires [100]; yet, relationship assessment tools have frequently been developed without the necessary validity evidence to support their use (see [16]).

Our approach highlights how bifactor techniques (including EBFA) may be a useful tool for overcoming constraints in traditional factor analytic approaches, enabling stronger tests of discriminant validity particularly when general factors are relevant. For example, our analyses of hundreds of items spanning a wide gamut of relationship instruments indicates that researchers can similarly identify, specify, and/or account for the influence of Q in latent variable models. It is important to note that we do not intend these data to serve as a novel self-report measure of relationship quality given that the very methods we used are what we critique as vulnerable to sentiment override and shared method variance. Within the context of self-report, however, our results suggest that researchers should explicitly model and account for Q when claiming to measure a construct that is theoretically or empirically distinct from global relationship evaluations. Future research can thus employ bifactor techniques (EBFA/CBFA) to evaluate a general Q-factor alongside items from a New Relationship Construct to ascertain whether items load meaningfully and discriminately onto a separate New Relationship Construct specific factor. If bifactor indices suggest no meaningful variance is explained above and beyond the influence of Q, then the new construct cannot be interpreted as capturing something substantive beyond (or alongside) global evaluations of relationship quality (i.e., Q).

Still, while establishing higher standards for discriminant validity is a step in the right direction, construct validation is an ongoing process that requires well-defined constructs that are separate from the measures being validated [101]. Related to this, most of the relationship variables encompassed in our investigation were evaluative in nature and involve tapping into individuals’ subjective judgments. In relationship science and similar research areas, researchers will be better positioned to identify meaningful predictors of relationship functioning and develop stronger theoretical and empirical tests of construct validity of their constructs and central principles by looking beyond purely evaluative relationship measures (e.g., frequency-based reports of discrete relationship behaviors or events to capture constructs related to conflict, communication, sexual activity, etc.). In turn, self-reports of one’s own personality, family background, and experiences of stress, are less conceptually proximal to evaluations of the relationship, and may thus be good candidates for predicting relationship quality in research relying on self-reports. Self-reports of more concrete variables (e.g., parental divorce, socioeconomic status, employment status) are more likely to avoid this problem as well (e.g., [102]). Explicitly linking proximal, medial, and distal levels of assessment (e.g., [103,104]) also represents a promising direction for pushing theory forward in relationship science.

Further, the findings highlight the limitations of obtaining self-reports from individuals, the most common method of data collection in relationship science. To be clear, this does not mean that self-report methods inherently lack value or should be outright abandoned. Yet, a clear implication of this work is that relationship science would greatly benefit from strategies that expand beyond self-report assessments as the primary method for conceptualizing and studying relationship phenomena. This is not to suggest that non self-report methods provide a simple solution, as recent work indicates that these approaches (e.g., experimental manipulations, behavioural coding schemes) may also face construct validity challenges [17]. In light of these considerations, our findings set the stage for future research to further probe the validity and reliability of relationship self-report measures across methodological contexts and conditions (e.g., dyadic, longitudinal, daily experience, qualitative designs). For example, further empirical work can examine the extent to which alternative methods to traditional self-report designs (e.g., observational, dyadic, longitudinal, daily experience, qualitative, third-party informant designs) are similarly affected by the effects of sentiment override, a point which we elaborate on below. In turn, our results suggest that certain analytic approaches may require more serious consideration, including the use of multitrait-multimethod examinations (MTMM) [100], statistical frameworks that decompose multiple sources of shared variance (e.g., the Social Relations Model; [105]), and latent profile or couple-centered approaches which aim to capture relationship features in ways that are individualized or tailored to different people [106,107].

### Limitations and future directions: Towards a more rigorous relationship science

The current research has several limitations that we view as important directions for future research. First, the current samples were designed to be representative of the broader population to which we would like to generalize (i.e., individuals in romantic relationships in the United States). However, our data is limited in terms of evaluating and contrasting the distinct experience of individuals across various populations, cultures, and contexts. Future research should aim to replicate the current results in more diverse samples, particularly in light of the fact that much of the relationship literature has historically concentrated on a narrow segment of the population (often White, college-educated, and middle-class) [18].

Second, the current studies were designed to capture a comprehensive set of relationship constructs representing measures of relationship quality (Study 1) and the field of relationship science more broadly (Study 2). As a result, some features of our study design limited the extent to which it fully mirrors a traditional relationship questionnaire (e.g., a standard intake survey). Our surveys included a high number and proportion of relationship measures relative to non-relationship measures (e.g., demographic measures, personality measures). We also did not administer complete instruments in their original form, since our item selection process focused on maximizing the breadth of item content across measures. These factors may have biased support for a general factor (Q), as responding to a large pool of similar-looking items could have promoted a relationship-response mindset among participants. Similarly, we cannot rule out that our inability to recover distinct, meaningful domains is also due to these specific methodological features. Thus, future studies could stress-test the current results by accounting for these factors within a more tightly-controlled and representative survey design. It is possible that some structural variations may emerge (e.g., increased evidence for select specific factors beyond Q). However, unless the pattern of results diverged substantially to support the Independence Model, our central conclusions regarding the influence of sentiment override would still hold.

Related to this, future studies could also assess whether the strength and stability of Q varies based on artifactual features of self-report methods more generally [77] or whether Q remains robust when key survey design features (e.g., survey length, the proportion and type of relationship items, temporal spacing of measures) are varied beyond the typical relationship-survey format we sought to approximate here. For example, support for sentiment override would consist of Q remaining relatively robust even upon implementing procedural techniques to prevent careless responding among survey respondents [71,108]. Such efforts would inform whether sentiment override poses a significant and pervasive threat, influencing responses whenever partners are asked to evaluate aspects of their relationship, or is contextually sensitive to varying features of the study design that could potentially be mitigated by researchers. Overall, examining the methodological contexts in which the general factor Q may be stronger or weaker would help determine whether scholars may need to revise their measures, methodological procedures, and/or theoretical models.

A key feature of our investigation was to employ the most widely used research design in the field, namely self-report measures. Specifically, we focused on a standard cross-sectional questionnaire design, limiting the generalizability of our findings beyond this context. It is possible that distinctions among constructs that could not be observed at a single measurement occasion might be observed in longitudinal or daily diary data capable of comparing how different items fluctuate or covary with each other across time [109]. For example, a strong test of the boundaries and pervasiveness of sentiment override would involve examining diary data studies with frequent assessments. Examining whether a general factor Q emerged in this context as well would be strong evidence of the pervasiveness of sentiment override in the literature.

Lastly, the current research is limited in its ability to speak to the substantive nature of Q. Our primary aim was not to assess sentiment override directly, but to characterize the variance structure under standard self-report conditions. Our interpretation of sentiment override is based on the strength and stability of Q (represented by global relational appraisals) in influencing participants’ evaluations across broad and specific aspects of their relationship. Our use of EBFA was relevant for assessing a sentiment override account as bifactor models partition covariance into general versus specific factors, providing direct estimates of the relative contributions of global versus domain-specific relationship judgements. Indeed, our finding that individuals do not appear to distinguish between different relationship-specific constructs converged across studies, and subsequent analyses using measures of general evaluative consistency bias, suggest that Q shares features of a more systematic response bias, but was specific to the domain of relationship evaluation. Stronger, dedicated examinations to substantiate this effect in other methodological contexts would be valuable. Such tests, by our estimation, could involve evaluating the strength of Q according to several other methodological and statistical techniques than those implemented in our studies. We briefly outline a few here. In principle, sentiment override is considered to be a fundamental mechanism governing the process of relationship evaluation, and not simply a methodological artifact. Thus, the general Q-factor should emerge rather consistently across different methods used to study relationship phenomena. Although self-report methods have predominated the field, it is important to note that relationship research regularly adopts multi-method approaches. Thus, multitrait–multimethod approaches [100] may be particularly useful in future studies to further establish the substantive nature of Q, as estimating the shared variance between evaluative relationship constructs across different methods (or rating sources) would be less susceptible to effects of method bias and/or artifacts than when using the same method [77].

A related question for future research is whether sentiment override is partner-specific or may generalize across different relationships. Examining partners in consensually non-monogamous (CNM) relationships may be particularly useful for probing this distinction, as it represents a context where need fulfillment can be spread across partners and overall relationship satisfaction may therefore be less tied to partner-specific evaluations [110]. Future work might consider adopting social relations model approaches (in which participants evaluate multiple close others on the same items) to cleanly separate participants’ overall evaluative tendencies (actor effects) from their tendency to apply a consistent evaluative lens to a given relationship (relationship effects; [111]).

## Conclusions

The current work represents a novel investigation of the empirical distinguishability between measures of relationship science’s most prominent constructs. We find that participants’ responses to diverse relationship inventories are principally guided by a global evaluation of the relationship. This is consistent with the phenomenon of sentiment override, and suggests that a notable amount of research in relationship science may be assessing constructs that are not empirically distinct from the outcomes they are being used to explain. To advance a deeper understanding of relationships, scholars will need to address this fundamental methodological constraint. It is clear that people can self-report how positively they feel about their relationship; what is not clear is whether they can self-report anything else about it.

## Supporting information

S1 File Supplemental Materials Document. (PDF)

S2 File Appendix A. (PDF)

S3 File Appendix B. (PDF)

S4 File Appendix C. (PDF)

S5 File Appendix D. (PDF)

## References

[1] Forscher BK. Chaos in the Brickyard. Science. 1963;142(3590):339. doi: 10.1126/science.142.3590.339 17799464

[2] Anvari F, Alsalti T, Oehler LA, Marion Z, Hussey I, Elson M, et al. A Fragmented Field: Construct and Measure Proliferation in Psychology. Advances in Methods and Practices in Psychological Science. 2025;8(3). doi: 10.1177/25152459251360642

[3] Hodson G. Construct jangle or construct mangle? Thinking straight about (nonredundant) psychological constructs. J Theor Soc Psychol. 2021;5(4):576–90.

[4] Gonzalez O, MacKinnon DP, Muniz FB. Extrinsic convergent validity evidence to prevent jingle and jangle fallacies. Multivariate Behav Res. 2021;56(1):3–19. doi: 10.1080/00273171.2019.1707061 31958017 PMC7369230

[5] Le H, Schmidt FL, Harter JK, Lauver KJ. The problem of empirical redundancy of constructs in organizational research: An empirical investigation. Organ Behav Hum Decis Process. 2010;112(2):112–25.

[6] Murphy BA, Hall JA, Duong F. It looks like construct validity, but look again: Comment on Clutterbuck *et al*. (2021) and recommendations for test developers in the broad “empathy” domain. Psychol Assess. 2022;34(4):397–404. doi: 10.1037/pas0001063 35377686

[7] Watson D, Clark LA. Negative affectivity: the disposition to experience aversive emotional states. Psychol Bull. 1984;96(3):465–90. doi: 10.1037/0033-2909.96.3.465 6393179

[8] Flake JK, Pek J, Hehman E. Construct validation in social and personality research: Current practice and recommendations. Soc Psychol Personal Sci. 2017;8(4).

[9] Robles TF, Slatcher RB, Trombello JM, McGinn MM. Marital quality and health: a meta-analytic review. Psychol Bull. 2014;140(1):140–87. doi: 10.1037/a0031859 23527470 PMC3872512

[10] Kamp Dush CM, Taylor MG, Kroeger RA. Marital Happiness and Psychological Well-Being Across the Life Course. Fam Relat. 2008;57(2):211–26. doi: 10.1111/j.1741-3729.2008.00495.x 23667284 PMC3650717

[11] Holt-Lunstad J, Birmingham W, Jones BQ. Is there something unique about marriage? The relative impact of marital status, relationship quality, and network social support on ambulatory blood pressure and mental health. Ann Behav Med. 2008;35(2):239–44. doi: 10.1007/s12160-008-9018-y 18347896

[12] Kiecolt-Glaser JK, Fisher LD, Ogrocki P, Stout JC, Speicher CE, Glaser R. Marital quality, marital disruption, and immune function. Psychosom Med. 1987;49(1):13–34. doi: 10.1097/00006842-198701000-00002 3029796

[13] Carr D, Freedman VA, Cornman JC, Schwarz N. Happy marriage, happy life? marital quality and subjective well-being in later life. J Marriage Fam. 2014;76(5):930–48. doi: 10.1111/jomf.12133 25221351 PMC4158846

[14] Finkel EJ, Simpson JA, Eastwick PW. The psychology of close relationships: fourteen core principles. Annu Rev Psychol. 2017;68:383–411. doi: 10.1146/annurev-psych-010416-044038 27618945

[15] Reis HT. Steps toward the ripening of relationship science. Pers Relatsh. 2007;14(1):1–23.

[16] CORE Lab. A novel, network-based approach to assessing romantic-relationship quality. Perspect Psychol Sci. 2024. doi: 10.1177/17456916231215248 38386418

[17] Joel S, Eastwick PW, Khera D. A Credibility revolution for relationship science: where can we step up our game? Social & Personality Psych. 2025;19(2). doi: 10.1111/spc3.70042

[18] Williamson HC, Bornstein JX, Cantu V, Ciftci O, Farnish KA, Schouweiler MT. How diverse are the samples used to study intimate relationships? A systematic review. J Soc Pers Relat. 2022;39(4):1087–109. doi: 10.1177/02654075211053849 35655791 PMC9159543

[19] Fletcher GJ, Simpson JA, Thomas G. The measurement of perceived relationship quality components: A confirmatory factor analytic approach. Pers Soc Psychol Bull. 2000;26(3):340–54.

[20] Bainbridge TF, Ludeke SG, Smillie LD. Evaluating the Big Five as an organizing framework for commonly used psychological trait scales. J Pers Soc Psychol. 2022;122(4):749–77. doi: 10.1037/pspp0000395 35025595

[21] Costa PTJr, McCrae RR. Domains and facets: Hierarchical personality assessment using the revised NEO personality inventory. J Pers Assess. 1995;64(1):21–50. 16367732 10.1207/s15327752jpa6401_2

[22] Lawson KM, Robins RW. Sibling constructs: what are they, why do they matter, and how should you handle them? Pers Soc Psychol Rev. 2021;25(4):344–66. doi: 10.1177/10888683211047101 34663112

[23] Thorndike EL. An introduction to the theory of mental and social measurements. New York: Columbia University Press; 1904.

[24] Kelley TL. Interpretation of educational measurements. New York: World Book Company; 1927.

[25] Fiske DW. Consistency of the factorial structures of personality ratings from different sources. J Abnorm Psychol. 1949;44(3):329–44. doi: 10.1037/h0057198 18146776

[26] Goldberg LR. An alternative “description of personality”: the big-five factor structure. J Pers Soc Psychol. 1990;59(6):1216–29. doi: 10.1037//0022-3514.59.6.1216 2283588

[27] Goode WJ, Hopkins E, McClure HM. Social systems and family patterns; a propositional inventory. Indianapolis: Bobbs-Merrill Co; 1971.

[28] Kelley HH, Berscheid E, Christensen A, Harvey JH, Huston TL, Levinger G. Close relationships. New York: Freeman; 1983.

[29] Joel S, Eastwick PW, Allison CJ, Arriaga XB, Baker ZG, Bar-Kalifa E, et al. Machine learning uncovers the most robust self-report predictors of relationship quality across 43 longitudinal couples studies. Proc Natl Acad Sci U S A. 2020;117(32):19061–71. doi: 10.1073/pnas.1917036117 32719123 PMC7431040

[30] Fincham FD, Rogge R. Understanding relationship quality: theoretical challenges and new tools for assessment. J Fam Theory Rev. 2010;2(4):227–42.

[31] Spanier GB. Measuring dyadic adjustment: New scales for assessing the quality of marriage and similar dyads. J Marriage Fam. 1976;38(1):15–28.

[32] Hendrick SS. A generic measure of relationship satisfaction. Journal of Marriage and the Family. 1988;50(1):93. doi: 10.2307/352430

[33] Karney BR, Bradbury TN. The longitudinal course of marital quality and stability: a review of theory, method, and research. Psychol Bull. 1995;118(1):3–34. doi: 10.1037/0033-2909.118.1.3 7644604

[34] Bradbury TN, Bodenmann G. Interventions for couples. Annu Rev Clin Psychol. 2020;16:99–123. doi: 10.1146/annurev-clinpsy-071519-020546 32031866

[35] Schumm WR, Paff-Bergen LA, Hatch RC, Obiorah FC, Copeland JM, Meens LD, et al. Concurrent and discriminant validity of the kansas marital satisfaction scale. Journal of Marriage and the Family. 1986;48(2):381. doi: 10.2307/352405

[36] Norton R. Measuring marital quality: a critical look at the dependent variable. Journal of Marriage and the Family. 1983;45(1):141. doi: 10.2307/351302

[37] Funk JL, Rogge RD. Testing the ruler with item response theory: increasing precision of measurement for relationship satisfaction with the Couples Satisfaction Index. J Fam Psychol. 2007;21(4):572–83. doi: 10.1037/0893-3200.21.4.572 18179329

[38] Fincham FD, Bradbury TN. The assessment of marital quality: a reevaluation. Journal of Marriage and the Family. 1987;49(4):797. doi: 10.2307/351973

[39] Jacobson NS, Moore D. Spouses as observers of the events in their relationship. J Consult Clin Psychol. 1981;49(2):269–77. doi: 10.1037//0022-006x.49.2.269 7217493

[40] Joel S, Maxwell JA, Khera D, Peetz J, Baucom BRW, MacDonald G. Expect and you shall perceive: People who expect better in turn perceive better behaviors from their romantic partners. J Pers Soc Psychol. 2023;124(6):1230–55. doi: 10.1037/pspi0000411 36442024

[41] Weiss RL. Strategic behavioral marital therapy: Toward a model for assessment and intervention. In: Vincent JP, editor. Adv Fam Interv Assess Theory. 1980. p. 229–71.

[42] Morin AJ, Myers ND, Lee S. Modern factor analytic techniques: Bifactor models, exploratory structural equation modeling (ESEM), and bifactor-ESEM. In: Tenenbaum G, Eklund RC, editors. Handbook of sport psychology. Hoboken: Wiley; 2020. p. 1044–73.

[43] Reise SP, Mansolf M, Haviland MG. Bifactor measurement models. In: Hoyle RH, editor. Handbook of structural equation modeling. New York: Guilford Press; 2023. p. 329–48.

[44] Rodriguez A, Reise SP, Haviland MG. Evaluating bifactor models: Calculating and interpreting statistical indices. Psychol Methods. 2016;21(2):137–50. doi: 10.1037/met0000045 26523435

[45] Rodriguez A, Reise SP, Haviland MG. Applying bifactor statistical indices in the evaluation of psychological measures. J Pers Assess. 2016;98(3):223–37. doi: 10.1080/00223891.2015.1089249 26514921

[46] Markon KE. Bifactor and Hierarchical Models: Specification, Inference, and Interpretation. Annu Rev Clin Psychol. 2019;15:51–69. doi: 10.1146/annurev-clinpsy-050718-095522 30649927

[47] Marsh HW, Morin AJS, Parker PD, Kaur G. Exploratory structural equation modeling: an integration of the best features of exploratory and confirmatory factor analysis. Annu Rev Clin Psychol. 2014;10:85–110. doi: 10.1146/annurev-clinpsy-032813-153700 24313568

[48] Wang YA, Eastwick PW. Solutions to the problems of incremental validity testing in relationship science. Pers Relatsh. 2020;27(1):156–75.

[49] Bradbury TN, Fincham FD, Beach SRH. Research on the nature and determinants of marital satisfaction: a decade in review. J of Marriage and Family. 2000;62(4):964–80. doi: 10.1111/j.1741-3737.2000.00964.x

[50] Fabrigar LR, Wegener DT. Exploratory factor analysis. New York: Oxford University Press; 2011.

[51] Loehlin JC, Beaujean AA. Latent variable models: An introduction to factor, path, and structural equation analysis. 5th ed. New York: Routledge; 2017.

[52] United States Census Bureau. 2020. https://data.census.gov/cedsci/table?q=United%20States&tid=ACSDP1Y2018.DP05&hidePreview=false

[53] Revelle WR. Psych: Procedures for personality and psychological research. 2021.

[54] R Core Team. R: A language and environment for statistical computing. Vienna: R Foundation for Statistical Computing; 2022. http://www.r-project.org/

[55] Costello AB, Osborne J. Best practices in exploratory factor analysis: four recommendations for getting the most from your analysis. Pract Assess Res Eval. 2005;10(1):7.

[56] Reise SP. Invited paper: the rediscovery of bifactor measurement models. Multivariate Behav Res. 2012;47(5):667–96. doi: 10.1080/00273171.2012.715555 24049214 PMC3773879

[57] Dueber DM, Toland MD. A bifactor approach to subscore assessment. Psychol Methods. 2023;28(1):222–41. doi: 10.1037/met0000459 34941326

[58] Reise SP, Bonifay WE, Haviland MG. Scoring and modeling psychological measures in the presence of multidimensionality. J Pers Assess. 2013;95(2):129–40. doi: 10.1080/00223891.2012.725437 23030794

[59] Gignac GE, Kretzschmar A. Evaluating dimensional distinctness with correlated-factor models: Limitations and suggestions. Intelligence. 2017;62:138–47. doi: 10.1016/j.intell.2017.04.001

[60] Reise SP, Scheines R, Widaman KF, Haviland MG. Multidimensionality and structural coefficient bias in structural equation modeling: A bifactor perspective. Educ Psychol Meas. 2013;73(1):5–26.

[61] Bader M, Jobst LJ, Moshagen M. Sample size requirements for bifactor models. Structural Equation Modeling: A Multidisciplinary Journal. 2022;29(5):772–83. doi: 10.1080/10705511.2021.2019587

[62] Abad FJ, Garcia-Garzon E, Garrido LE, Barrada JR. Iteration of partially specified target matrices: application to the bi-factor case. Multivariate Behav Res. 2017;52(4):416–29. doi: 10.1080/00273171.2017.1301244 28375697

[63] Garcia-Garzon E, Abad FJ, Garrido LE. On omega hierarchical estimation: a comparison of exploratory bi-factor analysis algorithms. Multivariate Behav Res. 2021;56(1):101–19. doi: 10.1080/00273171.2020.1736977 32449372

[64] Lorenzo-Seva U, Ferrando PJ. A general approach for fitting pure exploratory bifactor models. Multivariate Behav Res. 2019;54(1):15–30. doi: 10.1080/00273171.2018.1484339 30160535

[65] Mansolf M, Reise SP. Exploratory bifactor analysis: the schmid-leiman orthogonalization and jennrich-bentler analytic rotations. Multivariate Behav Res. 2016;51(5):698–717. doi: 10.1080/00273171.2016.1215898 27612521 PMC5425103

[66] Zhang B, Sun T, Cao M, Drasgow F. Using bifactor models to examine the predictive validity of hierarchical constructs: pros, cons, and solutions. Organizational Research Methods. 2020;24(3):530–71. doi: 10.1177/1094428120915522

[67] Garcia-Garzon E, Abad FJ, Garrido LE. Improving bi-factor exploratory modelling: empirical target rotation based on loading differences. Methodology. 2019;15:45–55.

[68] Dueber D. Bifactor indices calculator: Bifactoe indicates calculator. R package version 0.2.0. 2020. https://CRAN.R-project.org/package=BifactorIndicesCalaculator

[69] Watts AL, Makol BA, Palumbo IM, De Los Reyes A, Olino TM, Latzman RD, et al. How robust is the p factor? Using multitrait-multimethod modeling to inform the meaning of general factors of youth psychopathology. Clin Psychol Sci. 2022;10(4):640–61. doi: 10.1177/21677026211055170 36090949 PMC9454373

[70] Marsh HW, Hau KT, Balla JR, Grayson D. Is more ever too much? The number of indicators per factor in confirmatory factor analysis. Multivariate Behav Res. 1998;33(2):181–220. doi: 10.1207/s15327906mbr3302_1 26771883

[71] Stosic MD, Murphy BA, Duong F, Fultz AA, Harvey SE, Bernieri F. Careless responding: Why many findings are spurious or spuriously inflated. Adv Methods Pract Psychol Sci. 2024;7(1):25152459241231581.

[72] Peer E, Rothschild D, Gordon A, Evernden Z, Damer E. Data quality of platforms and panels for online behavioral research. Behav Res Methods. 2022;54(4):1643–62. doi: 10.3758/s13428-021-01694-3 34590289 PMC8480459

[73] Litman L, Robinson J, Rosenzweig C. The relationship between motivation, monetary compensation, and data quality among US- and India-based workers on Mechanical Turk. Behav Res Methods. 2015;47(2):519–28. doi: 10.3758/s13428-014-0483-x 24907001

[74] Anusic I, Schimmack U, Pinkus RT, Lockwood P. The nature and structure of correlations among Big Five ratings: the halo-alpha-beta model. J Pers Soc Psychol. 2009;97(6):1142–56. doi: 10.1037/a0017159 19968424

[75] Cooper WH. Ubiquitous halo. Psychological Bulletin. 1981;90(2):218.

[76] Laham SM, Forgas JP. Halo effects. Cognitive Illusions. Routledge; 2022. p. 259–71. doi: 10.4324/9781003154730-19

[77] Podsakoff PM, MacKenzie SB, Podsakoff NP. Sources of method bias in social science research and recommendations on how to control it. Annu Rev Psychol. 2012;63:539–69. doi: 10.1146/annurev-psych-120710-100452 21838546

[78] Van de Mortel TF. Faking it: Social desirability response bias in self-report research. Aust J Adv Nurs. 2008;25(4):40–8.

[79] Neff LA, Karney BR. To know you is to love you: the implications of global adoration and specific accuracy for marital relationships. J Pers Soc Psychol. 2005;88(3):480–97. doi: 10.1037/0022-3514.88.3.480 15740441

[80] Judge TA, Rodell JB, Klinger RL, Simon LS, Crawford ER. Hierarchical representations of the five-factor model of personality in predicting job performance: integrating three organizing frameworks with two theoretical perspectives. J Appl Psychol. 2013;98(6):875–925. doi: 10.1037/a0033901 24016206

[81] Graham JW, Taylor BJ, Olchowski AE, Cumsille PE. Planned missing data designs in psychological research. Psychol Methods. 2006;11(4):323–43. doi: 10.1037/1082-989X.11.4.323 17154750

[82] Zhang C, Yu MC. Planned missingness: how to and how much? Organizational Research Methods. 2022;25(4):623–41.

[83] Greszki R, Meyer M, Schoen H. The impact of speeding on data quality in nonprobability and freshly recruited probability-based online panels. In: Callegaro M, Baker R, Bethlehem J, Göritz AS, Krosnick JA, Lavrakas PJ, editors. Online panel research: A data quality perspective. London: Wiley;2014. p. 238–62.

[84] Baraldi AN, Enders CK. An introduction to modern missing data analyses. J Sch Psychol. 2010;48(1):5–37. doi: 10.1016/j.jsp.2009.10.001 20006986

[85] Nassiri V, Lovik A, Molenberghs G, Verbeke G. On using multiple imputation for exploratory factor analysis of incomplete data. Behav Res Methods. 2018;50(2):501–17. doi: 10.3758/s13428-017-1013-4 29392587

[86] van Buuren S. Multiple imputation of discrete and continuous data by fully conditional specification. Stat Methods Med Res. 2007;16(3):219–42. doi: 10.1177/0962280206074463 17621469

[87] Little RJA. Missing-data adjustments in large surveys. Journal of Business & Economic Statistics. 1988;6(3):287–96. doi: 10.1080/07350015.1988.10509663

[88] Rubin DB. Multiple imputation for nonresponse in surveys. New York: Wiley; 1987.

[89] Kam CCS. Careless responding threatens factorial analytic results and construct validity of personality measure. Front Psychol. 2019;10:1258. doi: 10.3389/fpsyg.2019.01258 31258500 PMC6587366

[90] Hawkins MW, Carrère S, Gottman JM. Marital Sentiment Override: Does It Influence Couples’ Perceptions?. J of Marriage and Family. 2002;64(1):193–201. doi: 10.1111/j.1741-3737.2002.00193.x

[91] Fincham FD, Linfield KJ. A new look at marital quality: Can spouses feel positive and negative about their marriage?. J Fam Psychol. 1997;11(4):489–502.

[92] Rogge RD, Fincham FD, Crasta D, Maniaci MR. Positive and negative evaluation of relationships: Development and validation of the Positive-Negative Relationship Quality (PN-RQ) scale. Psychol Assess. 2017;29(8):1028–43. doi: 10.1037/pas0000392 27736125

[93] Lawrance KA, Byers ES. Sexual satisfaction in long-term heterosexual relationships: The Interpersonal Exchange Model of Sexual Satisfaction. Pers Relatsh. 1995;2(4):267–85.

[94] Fincham FD, Garnier PC, Gano-Phillips S, Osborne LN. Preinteraction expectations, marital satisfaction, and accessibility: A new look at sentiment override. J Fam Psychol. 1995;9(1):3–14.

[95] Rusbult CE. Commitment and satisfaction in romantic associations: A test of the investment model. J Exp Soc Psychol. 1980;16(2):172–86.

[96] Cronbach LJ, Meehl PE. Construct validity in psychological tests. Psychol Bull. 1955;52(4):281–302. doi: 10.1037/h0040957 13245896

[97] Elson M, Hussey I, Alsalti T, Arslan RC. Psychological measures aren’t toothbrushes. Commun Psychol. 2023;1(1):25. doi: 10.1038/s44271-023-00026-9 39242966 PMC11332227

[98] van der Maas HLJ, Dolan CV, Grasman RPPP, Wicherts JM, Huizenga HM, Raijmakers MEJ. A dynamical model of general intelligence: the positive manifold of intelligence by mutualism. Psychol Rev. 2006;113(4):842–61. doi: 10.1037/0033-295X.113.4.842 17014305

[99] Orben A, Lakens D. Crud (Re)Defined. Adv Methods Pract Psychol Sci. 2020;3(2):238–47. doi: 10.1177/2515245920917961 33103054 PMC7116247

[100] Campbell DT, Fiske DW. Convergent and discriminant validation by the multitrait-multimethod matrix. Psychol Bull. 1959;56(2):81–105. doi: 10.1037/h0046016 13634291

[101] Schimmack U. The validation crisis in psychology. MP. 2021;5. doi: 10.15626/mp.2019.1645

[102] Tan JJX, Kraus MW, Carpenter NC, Adler NE. The association between objective and subjective socioeconomic status and subjective well-being: A meta-analytic review. Psychol Bull. 2020;146(11):970–1020. doi: 10.1037/bul0000258 33090862

[103] Eastwick PW, Finkel EJ, Simpson JA. Relationship trajectories: A meta-theoretical framework and theoretical applications. Psychol Inq. 2019;30(1):1–28.

[104] McNulty JK, Meltzer AL, Neff LA, Karney BR. How both partners’ individual differences, stress, and behavior predict change in relationship satisfaction: Extending the VSA model. Proc Natl Acad Sci U S A. 2021;118(27):e2101402118. doi: 10.1073/pnas.2101402118 34183417 PMC8271655

[105] Kenny DA, La Voie L. The social relations model. In: Berkowitz L, editor. Adv Exp Soc Psychol. 1984.

[106] Hagenaars JA, McCutcheon AL. Applied latent class analysis. Cambridge University Press; 2002.

[107] Eastwick PW, Finkel EJ, Joel S. Mate evaluation theory. Psychol Rev. 2023;130(1):211–41. doi: 10.1037/rev0000360 35389716

[108] Ward MK, Meade AW. Dealing with careless responding in survey data: prevention, identification, and recommended best practices. Annu Rev Psychol. 2023;74:577–96. doi: 10.1146/annurev-psych-040422-045007 35973734

[109] Courvoisier DS, Nussbeck FW, Eid M, Geiser C, Cole DA. Analyzing the convergent and discriminant validity of states and traits: development and applications of multimethod latent state-trait models. Psychol Assess. 2008;20(3):270–80. doi: 10.1037/a0012812 18778163

[110] Larva MA, Mogilski JK, Blumenstock SM. Nurturance, eroticism, and relationship satisfaction among people in monogamous and consensually non-monogamous relationships. The Journal of Sex Research. 2024;:1–3. 10.1080/00224499.2024.2435619 39692749

[111] Lakey B, Hubbard SA, Woods WC, Brummans J, Obreiter A, Fles E, et al. Supportive people evoke positive affect, but do not reduce negative affect, while supportive groups result from favorable dyadic, not group effects. Anxiety Stress Coping. 2022;35(3):323–38. doi: 10.1080/10615806.2021.1965995 34586940

