# Supplementary material for: Predicting relationship quality with itself? A single general factor captures most of the variance across 34 common relationship measures
Source: PLoS One. 2026 Apr 1;21(4):e0342451. doi: 10.1371/journal.pone.0342451 (PMC13042769; doi:10.1371/journal.pone.0342451)
Supplement: S1 File — (PDF) [file pone.0342451.s001.pdf]

Supplemental Materials

Supplemental materials (data, analysis scripts, output, summary tables, etc.) can be found on OSF: https://osf.io/e452p

Table of Contents

1. Participants 2
   1.1. Table S1. *Demographics of the Study 1 Sample* 2
   1.2 Table S2. *Demographics of the Study 2 Sample (N = 1439)* 3
   1.3 Study 1 Participant Recruitment Additional Details 4
2. Supplemental Information on Bifactor Analysis and Challenges of Interpreting General Factors 5
3. Study 1 and Study 2 Main Analyses 7
   3.1 Table S3. *Overview of EFA and EBFA Models Across Studies* 7
   3.2 Study 1 EFA Additional Details 8
4. Study 1 Review of Satisfaction/Relationship Quality Measures for Item Selection 8
   4.1 Table S4. *Sources, constructs, and number of retained items from 29 original instruments* 9
   4.2 Appendix S1: *Complete list of 206 items* 11
5. Study 2 Review of Satisfaction/Relationship Quality Measures for Item Selection 16
   5.1 Table S5. *Study 2 focal measures selection* 19
   5.2 Table S6. *Study 2 item selection information (constructs, sources, and citation count, and number of items included)* 22
6. Additional Information for Results of Study 2 Replication Analyses 25
   6.1 Table S7. *Study 2 EBFA 206-item Re-analysis: Top 10 Factor Loadings and Bifactor Indices* 27
7. Additional Information for Results of Study 2 Expansion Analyses 29
   7.1 Table S8. *Top Loading Items for the 3-factor EFA Model in Study 2 (408 items)* 30

8. EFA results by estimation method 31

9. EBFA results by rotation method..31

10. Item loading matrices for other EFA and EBFA solutions examined 34

11. Study 2 Data Screening: Participant Attentiveness Index 34

11.1 Table S9. *Item Set X* 35

12. Auxiliary CFA/CBFA tests of pre-registered factor models 35

12.1 Table S10. *Study 2 Bifactor indices for CBFA model with 3 specific factors (using reduced 102-item pool)* 38

13. Clarification of Preregistered vs Implemented Evaluative Bias Analyses 38

14. Auxiliary CFA/CBFA tests to Probe Content of Q 40

14.1 Table S11. *Confirmatory factor loading patterns from models testing Q as general evaluative consistency bias* 42

15. Auxiliary Analyses with Full Sample of Excluded Participants (Studies 1 and 2) 43

16. References for Study 1 measures listed in Table 1 and Appendix A of manuscript 44

1. Participants

The demographic characteristics for Studies 1 and 2 are presented below, along with census data targets used for recruitment.

1.1. Table S1. ***Demographics of the Study 1 Sample***

			Census Targets[1]
Variable	*N*	%	%
Age			
18-24 years	126	6.3	6.3
25-34 years	334	16.7	17.6
35-44 years	424	21.2	22.5
45-54 years	313	15.7	15.6
55-64 years	506	25.3	24.6
65+ years	297	14.9	13.4
Gender			
Male	947	47.4	47.3
Female	1053	52.7	52.7
Race/Ethnicity			
White/Caucasian	1368	68.4	66.4

Hispanic/Latino	208	10.4	11.8
Mixed	165	8.3	8.3
Black/African American	142	7.1	7.6
Asian or Pacific Islander	95	4.8	4.7
Other	16	.8	.84
American Indian or Aleut Eskimo	6	.3	.31
Annual Household Income			
Less than $30,000	261	13.1	12.6
$30,000-$49,999	290	14.5	13.5
$50,000-$74,999	395	19.8	19.6
$75,000-$99,999	343	17.2	18
$100,000-$149,999	354	17.7	18.7
$150,000 or greater	357	17.8	17.6
Education			
Less than High School	23	1.2	--
High School Diploma	315	15.8	--
Some College	480	24	--
College Degree	756	37.8	--
Post Graduate Degree	422	21.1	--

[1]Age and gender were derived from the 2010 United States decennial Census while income and race/ethnicity were derived from the 2019 American Community Survey.

1.2 Table S2. ***Demographics of the Study 2 Sample (N = 1439)***

			Census Targets[1]
Variable	*N*	%	%
Age			
18-24 years	73	5.07	6.3
25-34 years	264	18.35	17.6
35-44 years	307	21.33	22.5
45-54 years	252	17.51	15.6
55-64 years	268	18.62	24.6
65+ years	264	18.35	13.4
NA	11	.76	
Gender			
Male	573	39.82	47.3
Female	852	59.21	52.7
Race/Ethnicity			
White/Caucasian	895	62.20	59.71
Hispanic/Latino	233	16.19	18.62
Black/African American	167	11.61	12.58
Asian or Pacific Islander	57	3.96	6.06
Mixed	47	3.27	2.29
American Indian or Aleut Eskimo	18	1.25	.74
Other	17	1.18	
NA	5	.35	

Annual Household Income			
Less than $49,999	438	30.44	36.1
$50,000-$74,999	335	23.28	16.2
$75,000-$99,999	251	17.33	12.3
$100,000-$149,999	237	16.47	15.8
$150,000 or greater	167	11.61	19.7
NA	11		

[1]Age and gender for Study 2 were derived from the 2010 United States decennial Census while income and race/ethnicity were derived from 2020 US Census.

1.3 Study 1 Participant Recruitment Additional Details

Panel invitations and data cleaning was managed by Dynata for Study 1. However, we can report our recruitment targets based on our inclusion criteria (e.g., relationship status) and the actual number of respondents who entered the survey. Based on an initial estimated incidence rate of 62% (i.e., the proportion of the general population in a committed relationship), we projected that approximately n = 4,838 respondents would need to click into the survey to obtain 3,000 qualified completes (the recruitment target). As data collection progressed, the observed incidence rate dropped to 51% as we tried to collect more niche demographic groups to reach our census targets. Overall, 5,851 respondents ultimately clicked into the survey to yield 3,001 qualified participants, who we used as the starting point for our Study 1 screening and analyses.

2. Supplemental Information on Bifactor Analysis and Challenges of Interpreting General Factors

Once a comprehensive collection of self-report relationship questionnaire items is compiled, it is reasonable to presume that each item may be subjected to at least two sources of substantive variance: (a) global assessments of relationship quality driven by sentiment override and (b) residual variance that pulls some subsets of items together based on their more specific contents. In such cases, bifactor models are ideal as they estimate both general and specific sources of covariance, unlike the more common exploratory factor analysis (EFA) and confirmatory factor analysis (CFA). In contrast to traditional factor analytic techniques (EFA and CFA), bifactor modeling quantifies the extent to which each item is influenced by both the general factor and potentially by one or more specific factors, an approach that is particularly advantageous when trying to understand the structure of multiple overlapping constructs (Markon, 2019; Reise et al., 2023).

Employing exploratory bifactor analyses served as an optimal approach for uncovering these patterns in initial investigations, whereby results could then be used for corroboratory tests in subsequent investigations. In particular, a variety of bifactor psychometric indices can inform of the unique covariance accounted for by the specific factors versus the general factor; such indices can help scholars adjudicate whether assessing specific subdomains within multidimensional item pools provides meaningful value, and/or whether aggregating subscales together produces adequate unidimensional scores (Rodriguez et al., 2016a). For example, Rodriguez et al. (2016b) investigated bifactor models across a wide range of ostensibly multidimensional psychopathology questionnaires, and demonstrated that standard model fit indices do not necessarily reflect the quality of a measurement model. Although

multidimensional representations of these questionnaires attained “good model fit,” for a large majority of them, the individual subscales typically provided very little meaningful reliable variance above and beyond their respective general factors.

Findings from such an approach would have implications for evaluating existing theoretical frameworks in relationship science. For example, the Perceived Relationship Quality Components model (PRQC; Fletcher et al., 2000) remains one of the few direct attempts in the field to empirically delineate the boundaries of relationship quality. The PRQC model describes relationship quality with a higher-order factor model, in which a single, general relationship quality factor is reflected by six *quasi-independent* relationship constructs (satisfaction, commitment, intimacy, trust, passion, and love). Using methods available at the time (i.e., CFA), this model was shown to fit a dataset well, but does not offer clear guidance about whether overlapping constructs such as love, trust, and commitment should be treated as predictors of relationship quality or facets of it, nor help in determining whether other (particularly newer) relationship constructs are separate from relationship quality. Because standard CFA approaches do not partition the unique covariance accounted for by specific factors from that of a potential general factor, they leave interpretations regarding the incremental validity of any constructs unclear (Wang & Eastwick, 2020). To the extent that a general factor explains the vast majority of the variance among constructs considered theoretically distinct, then there is little empirical justification for considering those constructs separately.

One caveat in bifactor modeling is worth mentioning at the outset: the challenges of interpreting general factors. Identifying a general factor that accounts for substantial variance in a broad collection of indicators does not speak to what that general factor “means” substantively (i.e., the results do not by themselves specify what the indicators have in common that is

producing this general factor). Moreover, the general factor will reflect not only construct-relevant substantive variance across a collection of indicators, but also construct-irrelevant substantive variance and/or method variance that cuts across the item pool (e.g., social desirability bias or a broader evaluative consistency bias not specific to relationship perceptions; acquiescent or extreme responding; see Watts et al., 2020). For these reasons, substantive claims about general factors continue to be debated in many fields, such as with the "*p*-factor" of psychopathology (e.g., Caspi et al., 2014), the "General Factor of Personality" (e.g., Musek, 2007), and the "Dark Factor of Personality" (e.g., Moshagen, 2018).

3. Study 1 and Study 2 Main Analyses

3.1 Table S3. *Overview of EFA and EBFA Models Across Studies*

Study (Item Set)	Analysis	Primary Extraction (Secondary)	Factor solutions examined	Primary Aim	Key Finding
Study 1 (206 satisfaction items)	EFA	Promax, ML (minres; PA)	1, 2, 3, 4, 12, 13, 17, 20	Identify underlying dimensionality of full item pool	3-factor solution (Positive, Negative, Sex) most interpretable; higher factor-models over-extracted / uninterpretable.
Study 1 (206 satisfaction items)	EBFA	SLiD (Sli; SL; bi-geomin, bi-quartimin; DSL; DBF)	Q + 1SF<br>Q + 2SF<br>Q + 3SF	Assess strength of general factor (Q) vs. specific factors	Strong Q (e.g., SliD: ωH ≈ .92, ECV ≈ .73); SF (Positive, Negative, Sex) show limited incremental variance (low ωHS / ECV_{SS})
Study 2 direct replication (206 satisfaction items)	EFA	Promax, ML (minres; PA)	1, 2, 3, 4, 5, 11, 20, 32	Replicate Study 1 dimensionality with new sample	Same 3-factor structure recovered; higher-factor models again over-extracted / uninterpretable.
Study 2 direct replication (206 satisfaction items)	EBFA	SliD (Sli; SL; bi-geomin, bi-quartimin; DSL; DBF)	Q + 2SF<br>Q + 3SF<br>Q + 4SF	Replicate Q and SF pattern from Study 1	Convergent pattern with Study 1: dominant Q factor; SFs show limited incremental variance.
Study 2 (408 relationship items)	EFA	Promax, ML (minres; PA)	1, 2, 3, 4, 6, 11, 15, 16, 17	Expand analysis to broader relationship item pool	3-factor solution (Positive, Negative/power, Sex) again best; higher-factor models over-extracted / uninterpretable.
Study 2 (408	EBFA	SliD (Sli; SL; bi-geomin, bi-	Q + 2SF<br>Q + 3SF<br>Q + 4SF	Assess whether broader content	Q remains dominant (e.g., SliD: ωH ≈ .69, ECV ≈ .82); SF (Negative/power, Sex,

relationship items)	quartimin; DSL; DBF)	strengthens specific factors	Emotional attachment) show limited incremental variance (low ωHS / ECV_{SS})

Notes. ML = maximum likelihood, minres = minimum residual, PA = principal axis, SliD = empirical iterative empirical target rotation based on an initial SL solution, Sli = iterative empirical target rotation based on an initial SL solution, SL = Schmid-Leiman, DSL = direct Schmid-Leiman, DBF = direct bifactor. Q refers to the general factor identified in analyses. SF = specific factors. The EBFA models specified were based on EFA results.

3.2 Study 1 EFA Additional Details

As noted in the manuscript, results suggested different potential factor solutions across selection criteria. Specifically, scree plots suggested 2 factors (with eigenvalues indicating a very large first factor compared to subsequent extracted factors), hierarchical cluster analysis suggested 3 factors, BIC suggested 12 factors, parallel analyses suggested 13 factors, MAP suggested 17 factors, and SABIC suggested 20 factors. We applied our evaluation criteria (i.e., primary loading > .30 and cross-loadings < .30) to interpret the viability of each factor solution.

4. Study 1 Review of Satisfaction/Relationship Quality Measures for Item Selection

A review of research on relationship satisfaction and its proximal correlates was conducted by the identified 29 different satisfaction measures that have been commonly administered by relationship scientists in multiple studies. All of the measures ask respondents to make an evaluative rating on some aspect of their intimate relationships, and the constructs range from relationship satisfaction itself to constructs presumed to account for variance in satisfaction across individuals (e.g., trust, partner responsiveness, conflict). Many of these instruments contained multiple subscales measuring separate constructs; together they comprised 512 items representing 27 different content areas.

The item set contained numerous items that were identical or almost identical in content. An expert panel, consisting of five of the authors (specifically the 3rd, 5th, 6th, 7th and 9th author) consolidated these items. Members of the panel were qualified to assess items given their considerable expertise on the topic of marital satisfaction and stability, and on research and

theory in the field of relationship science. Each item was reviewed, eliminating those that duplicated or nearly duplicated other items, revising the wording where necessary to ensure that all items referred to intimate relationships generally rather than marriages (e.g., changing "My spouse cares for me" to "My partner cares for me"), revising the wording from questions to statements where necessary (e.g., "How often does your partner express gratitude toward you?" to "My partner expresses gratitude toward me often."), separating double-barreled items into separate statements (e.g., splitting "I am not happy about our communication and feel my partner does not understand me" into "I am not happy about our communication" and "I feel my partner does not understand me"), and eliminating technical language and jargon (e.g., dropping "I am oriented toward the long-term future of my relationship"). By the end of this review, 206 unique items had been retained, including multiple items assessing each of the original 27 content domains. Table S1 presents the scales, sources, constructs, and number of retained items from each of the original instruments, as well as how often the scale has been cited according to Google Scholar. Appendix S1 presents the complete list of 206 items and their sources. For each item, participants were asked to rate their agreement with each statement on a 6-point scale from 0 (Not at all Agree) to 5 (Completely Agree).

4.1 Table S4. *Sources, constructs, and number of retained items from 29 original instruments*

Scale	Source	Construct/Subscale	# of Items Used	# of Citations
Appreciation in Relationships (AIR)	Gordon et al. (2012)	Appreciation by the Partner, Appreciation for the Partner	9	312
Barrett-Lennard Relationship Inventory (OS-40)	Barrett-Lennard (1981)	Appreciation by the Partner, Expressing Affection, Perceived Partner Regard, Support, Trust	9	911
Commitment Inventory	Stanley & Markman (1992)	Own Commitment	2	1,055
Communal Strength Scale	Mills et al. (2004)	Communal Strength	4	315
Communication Patterns Questionnaire (CPQ)	Christensen & Sullaway (1984)	Being Understood, Communication	12	455

Couples Satisfaction Index (CSI-16)	Funke & Rogge (2007)	Global Positive Sentiment, Perceived Partner Satisfaction	4	1,702
Dyadic Adjustment Scale (DAS)	Spanier (1976)	Divorce Proneness, Global Negative Sentiment, Perceived Similarity, Shared Activities	7	10,915
Dyadic Trust Scale	Larzelere & Huston (1980)	Critique of the Partner, Trust	4	2,005
ENRICH Marital Satisfaction Scale (EMS)	Fowers & Olson (1993)	Communication, Idealistic Distortion, Perceived Similarity	4	665
Frequency and Acceptability of Partner Behavior	Doss & Christensen (2006)	Critique of the Partner, Expressing Affection, Support, Trust	5	33
Friendship Network Satisfaction Scale (adapted)	Kaufman et al. (2021)	Being Understood, Communication, Own Commitment, Shared Activities, Support	9	0
Braiker-Kelley Partnership Questionnaire	Braiker & Kelley (1979)	Communication, Conflict, Global Negative Sentiment, Own Commitment, Perceived Partner Commitment	7	1,194
"Saying 'Thank You': Partners' expressions of gratitude..."	Park et al. (2019)	Appreciation by the Partner	1	38
Index of Sexual Satisfaction (ISS)	Hudson, Harrison, & Crosscup (1981)	Sex	2	468
Investment Model Scale	Rusbult, Martz, & Agnew (1998)	Global Positive Sentiment, Investment, Own Commitment, Perceived Partner Commitment	11	2,661
Kansas Marital Satisfaction Scale (KMS)	Schumm et al. (1986)	Global Positive Sentiment	1	1,143
Marital Instability Scale	Booth, Johnson, & Edwards (1983)	Divorce Proneness	4	530
Marital Problems Scale	Amato & Rogers (1997)	Critique of the Partner	7	1,149
Marital Satisfaction Inventory (MSI)	Snyder (1979)	Communication, Conflict, Future Expectations, Global Negative Sentiment, Perceived Similarity	5	686
Marital Satisfaction Scale (MSS)	Blum & Mehrabian (1999)	Communication, Conflict, Critique of the Partner, Expressing Affection, Global Negative Sentiment, Global Positive Sentiment, Perceived Partner Satisfaction, Perceived Similarity, Sex, Shared Activities, Trust	21	309
Marital Satisfaction Scale (MSS)	Roach, Frazier, & Bowden (1981)	Admiration for the Partner, Communication, Critique of the Partner, Future Expectations, Global Negative Sentiment, Global Positive Sentiment, Perceived Partner Regard, Role Fulfillment, Shared Activities, Trust	21	472
Perceived Partner Responsiveness Scale (PPR)	Reis et al. (2017)	Being Understood, Empathy, Shared Activities	5	67
Perceived Relationship Quality Component (PRQC)	Fletcher, Simpson, & Thomas (2000)	Admiration for the Partner, Global Positive Sentiment, Perceived Partner Satisfaction, Perceived Partner Commitment, Sex, Trust	6	1,069
Personal Assessment of Intimacy in Relationships (PAIR)	Schaefer & Olson (1981)	Communication, Critique of the Partner, Empathy, Global Negative Sentiment, Idealistic Distortion, Perceived Similarity, Sex, Shared Activities, Socializing with Friends	18	1,246

Positive and Negative Semantic Differential (PN-SMD)	Mattson et al. (2012)	Global Negative Sentiment, Global Positive Sentiment	4	109
Quality of Marriage Index (QMI)	Norton (1983)	Global Positive Sentiment	3	2,332
Quality of Sex Inventory (QSI)	Shaw & Rogge (2016)	Sexual Satisfaction	6	41
Relationship Assessment Scale (RAS)	Hendrick (1988)	Global Negative Sentiment, Global Positive Sentiment	2	2,779
Relationship Satisfaction Scale (RS)	Roysamb, Vitterso, & Tambs (2014)	Being Understood, Conflict, Divorce Proneness, Global Positive Sentiment, Perceived Similarity	5	50

4.2 Appendix S1: *Complete list of 206 items*

Dimension	Item	Scale	Source
Admiration for the Partner	I adore my partner.	Perceived Relationship Quality Component (PRQC)	Fletcher, Simpson, & Thomas (2000)
	My partner inspires me to do my best work.	Marital Satisfaction Scale (MSS)	Roach, Frazier, & Bowden (1981)
	My partner is willing to make helpful improvements in our relationship.	Marital Satisfaction Scale (MSS)	Roach, Frazier, & Bowden (1981)
	My partner is one of the best people I know.	Marital Satisfaction Scale (MSS)	Roach, Frazier, & Bowden (1981)
	I feel very lucky to have my partner in my life.	--	New
Appreciation by the Partner	I know I’m valued and appreciated by my partner.	Barrett-Lennard Relationship Inventory (OS-40)	Barrett-Lennard (1981)
	My partner makes sure I feel appreciated.	Appreciation in Relationships (AIR)	Gordon et al. (2012)
	My partner often tells me the things that s/he really likes about me.	Appreciation in Relationships (AIR)	Gordon et al. (2012)
	At times my partner takes me for granted. (-)	Appreciation in Relationships (AIR)	Gordon et al. (2012)
	My partner doesn’t notice when I do nice things for her/him.(-)	Appreciation in Relationships (AIR)	Gordon et al. (2012)
	My partner makes me feel special.	Appreciation in Relationships (AIR)	Gordon et al. (2012)
	My partner expresses gratitude towards me often.	Single item from "Saying 'thank you': Partners' expressions of gratitude..."	Park et al. (2019)
Appreciation for the Partner	I tell my partner often that s/he is the best.	Appreciation in Relationships (AIR)	Gordon et al. (2012)
	At times I take my partner for granted. (-)	Appreciation in Relationships (AIR)	Gordon et al. (2012)
	I appreciate my partner.	Appreciation in Relationships (AIR)	Gordon et al. (2012)
	I make sure my partner feels appreciated.	Appreciation in Relationships (AIR)	Gordon et al. (2012)
Being Understood	My partner and I feel understood by each other.	Communication Patterns Questionnaire (CPQ)	Christensen & Sullaway (1984)
	My partner understands me.	Perceived Partner Responsiveness Scale (PPR)	Reis et al. (2017)
	My partner knows me well.	Perceived Partner Responsiveness Scale (PPR)	Reis et al. (2017)
	My partner is generally understanding.	Relationship Satisfaction Scale (RS)	Roysamb, Vitterso, & Tambs (2014)
Communication	My partner and I do not communicate well with each other. (-)	Marital Satisfaction Scale (MSS)	Blum & Mehrabian (1999)
	My partner and I settle our disagreements with mutual give and take.	Marital Satisfaction Scale (MSS)	Blum & Mehrabian (1999)
	I tell my partner what I want or need from the relationship.	Single item from “Conflict in the development of close relationships”	Braiker & Kelley (1979)
	My partner and I talk about the quality of our relationship often.	Single item from “Conflict in the development of close relationships”	Braiker & Kelley (1979)

	My partner and I avoid discussing our problems. (-)	Communication Patterns Questionnaire (CPQ)	Christensen & Sullaway (1984)
	My partner and I blame, accuse, and criticize one another. (-)	Communication Patterns Questionnaire (CPQ)	Christensen & Sullaway (1984)
	My partner and I express our feelings to each other.	Communication Patterns Questionnaire (CPQ)	Christensen & Sullaway (1984)
	When we have problems, my partner and I suggest possible solutions and compromises.	Communication Patterns Questionnaire (CPQ)	Christensen & Sullaway (1984)
	When we have problems, my partner and I threaten one another with negative consequences. (-)	Communication Patterns Questionnaire (CPQ)	Christensen & Sullaway (1984)
	When we have problems, my partner and I try to be especially nice to each other.	Communication Patterns Questionnaire (CPQ)	Christensen & Sullaway (1984)
	My partner and I try to discuss our problems.	Communication Patterns Questionnaire (CPQ)	Christensen & Sullaway (1984)
	When we have problems, I call my partner names, swear at my partner, or attack my partner's character. (-)	Communication Patterns Questionnaire (CPQ)	Christensen & Sullaway (1984)
	When we have problems, I push, shove, slap, hit, or kick my partner. (-)	Communication Patterns Questionnaire (CPQ)	Christensen & Sullaway (1984)
	When we have problems, my partner calls me names, swears at me, or attacks my character. (-)	Communication Patterns Questionnaire (CPQ)	Christensen & Sullaway (1984)
	When we have problems, my partner pushes, shoves, slaps, hits, or kicks me. (-)	Communication Patterns Questionnaire (CPQ)	Christensen & Sullaway (1984)
	I am very happy about how we make decisions and resolve conflicts.	ENRICH Marital Satisfaction Scale (EMS)	Fowers & Olson (1993)
	I have meaningful conversations with my partner.	Friendship Network Satisfaction Scale (adapted)	Kaufman et al. (2021)
	When I have a problem, I can talk to my partner about it.	Friendship Network Satisfaction Scale (adapted)	Kaufman et al. (2021)
	My partner gives me sufficient opportunity to express my opinions.	Marital Satisfaction Scale (MSS)	Roach, Frazier, & Bowden (1981)
	I feel it is useless to discuss some things with my partner. (-)	Personal Assessment of Intimacy in Relationships (PAIR)	Schaefer & Olson (1981)
	My partner helps me clarify my thoughts.	Personal Assessment of Intimacy in Relationships (PAIR)	Schaefer & Olson (1981)
	My partner and I seem able to go for days sometimes without settling our differences. (-)	Marital Satisfaction Inventory (MSI)	Snyder (1979)
Conflict	My partner and I often argue about finances. (-)	Marital Satisfaction Scale (MSS)	Blum & Mehrabian (1999)
	My partner and I argue with each other often. (-)	Single item from “Conflict in the development of close relationships”	Braiker & Kelley (1979)
	My partner and I have problems in our relationship. (-)	Relationship Satisfaction scale (RS)	Roysamb, Vitterso, & Tambs (2014)
	Minor disagreements with my partner often end up in big arguments. (-)	Marital Satisfaction Inventory (MSI)	Snyder (1979)
Critique of the Partner	My partner gets angry easily. (-)	Marital Problems Scale	Amato & Rogers (1997)
	My partner has feelings that are easily hurt. (-)	Marital Problems Scale	Amato & Rogers (1997)
	My partner is jealous. (-)	Marital Problems Scale	Amato & Rogers (1997)
	My partner is domineering. (-)	Marital Problems Scale	Amato & Rogers (1997)
	My partner is critical. (-)	Marital Problems Scale	Amato & Rogers (1997)
	My partner is moody. (-)	Marital Problems Scale	Amato & Rogers (1997)
	My partner drinks or uses drugs. (-)	Marital Problems Scale	Amato & Rogers (1997)
	I don't approve of the way my partner relates to my family. (-)	Marital Satisfaction Scale (MSS)	Blum & Mehrabian (1999)
	My partner's habits annoy me. (-)	Marital Satisfaction Scale (MSS)	Blum & Mehrabian (1999)
	My partner is too flirtatious with other men/women. (-)	Frequency and Acceptability of Partner Behavior (adapted)	Doss & Christensen (2006)
	I feel that my partner does not show me enough consideration. (-)	Dyadic Trust Scale	Larzelere & Huston (1980)
	My partner is primarily interested in his/her own welfare. (-)	Dyadic Trust Scale	Larzelere & Huston (1980)
	My partner treats me fairly and justly. (-)	Dyadic Trust Scale	Larzelere & Huston (1980)
	My partner gets me badly flustered and jittery. (-)	Marital Satisfaction Scale (MSS)	Roach, Frazier, & Bowden (1981)
	My partner makes unfair demands on my free time. (-)	Marital Satisfaction Scale (MSS)	Roach, Frazier, & Bowden (1981)
	My partner frequently tries to change my ideas. (-)	Personal Assessment of Intimacy in Relationships (PAIR)	Schaefer & Olson (1981)

Divorce Proneness	I would enjoy living apart from my partner. (-)	Marital Instability Scale	Booth, Johnson, & Edwards (1983)
	My spouse thinks our relationship is in trouble. (-)	Marital Instability Scale	Booth, Johnson, & Edwards (1983)
	My spouse has seriously suggested the idea of ending the relationship. (-)	Marital Instability Scale	Booth, Johnson, & Edwards (1983)
	I have discussed ending the relationship with friends and family members. (-)	Marital Instability Scale	Booth, Johnson, & Edwards (1983)
	I often consider ending our relationship. (-)	Relationship Satisfaction scale (RS)	Roysamb, Vitterso, & Tambs (2014)
	My partner and I often discuss or consider divorce, separation, or terminating our relationship. (-)	Dyadic Adjustment Scale (DAS)	Spanier (1976)
Empathy	My partner usually is responsive to my needs.	Perceived Partner Responsiveness Scale (PPR)	Reis et al. (2017)
	My partner usually seems interested in what I am thinking and feeling.	Perceived Partner Responsiveness Scale (PPR)	Reis et al. (2017)
	My partner listens to me when I need someone to talk to.	Personal Assessment of Intimacy in Relationships (PAIR)	Schaefer & Olson (1981)
Expressing Affection	My partner is friendly and warm toward me.	Barrett-Lennard Relationship Inventory (OS-40)	Barrett-Lennard (1981)
	I don't get the love and affection I want from my partner.	Marital Satisfaction Scale (MSS)	Blum & Mehrabian (1999)
	My partner and I kiss daily.	Marital Satisfaction Scale (MSS)	Blum & Mehrabian (1999)
	My partner is very loving and affectionate.	Marital Satisfaction Scale (MSS)	Blum & Mehrabian (1999)
	My partner often compliments me.	Frequency and Acceptability of Partner Behavior (adapted)	Doss & Christensen (2006)
	My partner often tells me s/he loves me.	Frequency and Acceptability of Partner Behavior (adapted)	Doss & Christensen (2006)
Future Expectations	The future of my relationship looks promising to me.	Marital Satisfaction Scale (MSS)	Roach, Frazier, & Bowden (1981)
	The future of our relationship is too uncertain to make serious plans. (-)	Marital Satisfaction Inventory (MSI)	Snyder (1979)
Global Negative Sentiment	My relationship is not as good as most marriages. (-)	Marital Satisfaction Scale (MSS)	Blum & Mehrabian (1999)
	I often feel angry or resentful toward my partner. (-)	Single item from “Conflict in the development of close relationships”	Braiker & Kelley (1979)
	I often wish I hadn't gotten into this relationship. (-)	Relationship Assessment Scale (RAS)	Hendrick (1988)
	My relationship is boring. (-)	Positive and Negative Semantic Differential (PN-SMD)	Mattson et al. (2012)
	My relationship is empty. (-)	Positive and Negative Semantic Differential (PN-SMD)	Mattson et al. (2012)
	My relationship is miserable. (-)	Positive and Negative Semantic Differential (PN-SMD)	Mattson et al. (2012)
	I become upset, angry, or irritable because of things that occur in my relationship. (-)	Marital Satisfaction Scale (MSS)	Roach, Frazier, & Bowden (1981)
	I get discouraged trying to make my relationship work out. (-)	Marital Satisfaction Scale (MSS)	Roach, Frazier, & Bowden (1981)
	I worry a lot about my relationship. (-)	Marital Satisfaction Scale (MSS)	Roach, Frazier, & Bowden (1981)
	My relationship is definitely unhappy. (-)	Marital Satisfaction Scale (MSS)	Roach, Frazier, & Bowden (1981)
	I feel neglected at times by my partner. (-)	Personal Assessment of Intimacy in Relationships (PAIR)	Schaefer & Olson (1981)
	I often feel distant from my partner. (-)	Personal Assessment of Intimacy in Relationships (PAIR)	Schaefer & Olson (1981)
	My relationship has been disappointing in several way.s (-)	Marital Satisfaction Inventory (MSI)	Snyder (1979)
	My partner and I often get on each other's nerves. (-)	Dyadic Adjustment Scale (DAS)	Spanier (1976)
Global Positive Sentiment	My partner and I have a better relationship than most couples I know.	Marital Satisfaction Scale (MSS)	Blum & Mehrabian (1999)
	I love my partner very much.	Perceived Relationship Quality Component (PRQC)	Fletcher, Simpson, & Thomas (2000)
	I have a warm and comfortable relationship with my partner.	Couples Satisfaction Index (CSI-16)	Funke & Rogge (2007)
	Our relationship is strong.	Couples Satisfaction Index (CSI-16)	Funke & Rogge (2007)
	My relationship with my partner is rewarding.	Couples Satisfaction Index (CSI-16)	Funke & Rogge (2007)
	My partner meets my needs.	Relationship Assessment Scale (RAS)	Hendrick (1988)
	My relationship is enjoyable.	Positive and Negative Semantic Differential (PN-SMD)	Mattson et al. (2012)

	All things considered, I am very happy in my relationship.	Quality of Marriage Index (QMI)	Norton (1983)
	I really feel like part of a team with my partner.	Quality of Marriage Index (QMI)	Norton (1983)
	My relationship with my partner is very stable.	Quality of Marriage Index (QMI)	Norton (1983)
	I feel competent and fully able to handle my relationship.	Marital Satisfaction Scale (MSS)	Roach, Frazier, & Bowden (1981)
	I get along well with my partner.	Marital Satisfaction Scale (MSS)	Roach, Frazier, & Bowden (1981)
	I have made a success of my relationship so far.	Marital Satisfaction Scale (MSS)	Roach, Frazier, & Bowden (1981)
	My life would seem empty without my relationship.	Marital Satisfaction Scale (MSS)	Roach, Frazier, & Bowden (1981)
	My relationship helps me toward the goals I have set for myself.	Marital Satisfaction Scale (MSS)	Roach, Frazier, & Bowden (1981)
	I have a close relationship with my partner.	Relationship Satisfaction Scale (RS)	Roysamb, Vitterso, & Tambs (2014)
	My relationship is close to ideal.	Investment Model Scale	Rusbult, Martz, & Agnew (1998)
	I am satisfied with my partner.	Kansas Marital Satisfaction Scale (KMS)	Schumm et al. (1983)
Idealistic Distortion	I have never regretted my relationship with my partner, not even for a moment.	ENRICH Marital Satisfaction Scale (EMS)	Fowers & Olson (1993)
	Our relationship is a perfect success.	ENRICH Marital Satisfaction Scale (EMS)	Fowers & Olson (1993)
	My partner has all the qualities I've ever wanted in a mate.	Personal Assessment of Intimacy in Relationships (PAIR)	Schaefer & Olson (1981)
Investment	I have invested a great deal of time in our relationship.	Investment Model Scale	Rusbult, Martz, & Agnew (1998)
	I have told my partner many private things about myself.	Investment Model Scale	Rusbult, Martz, & Agnew (1998)
	My partner and I share many memories.	Investment Model Scale	Rusbult, Martz, & Agnew (1998)
	Many aspects of my life have become linked to my partner.	Investment Model Scale	Rusbult, Martz, & Agnew (1998)
	I have invested great deal in our relationship that I would lose if we were to break up.	Investment Model Scale	Rusbult, Martz, & Agnew (1998)
Communal Strength	I am happy when I do something that helps my partner.	Communal Strength Scale	Mills et al. (2004)
	Meeting the needs of my partner is a high priority for me.	Communal Strength Scale	Mills et al. (2004)
	I would be willing to give up a lot to benefit my partner.	Communal Strength Scale	Mills et al. (2004)
	I would go out of my way to do something for my partner.	Communal Strength Scale	Mills et al. (2004)
Own Commitment	I feel trapped in my relationship.	Single item from “Conflict in the development of close relationships”	Braiker & Kelley (1979)
	I feel very attached to my partner.	Single item from “Conflict in the development of close relationships”	Braiker & Kelley (1979)
	It is hard to imagine my life without my partner.	Friendship Network Satisfaction Scale (adapted)	Kaufman et al. (2021)
	I am committed to maintaining my relationship with my partner	Investment Model Scale	Rusbult, Martz, & Agnew (1998)
	I want our relationship to last for a very long time.	Investment Model Scale	Rusbult, Martz, & Agnew (1998)
	I would feel very upset if our relationship were to end in the near future.	Investment Model Scale	Rusbult, Martz, & Agnew (1998)
	I want this relationship to stay strong no matter what rough times we may encounter.	Commitment Inventory	Stanley & Markman (1992)
	I want to grow old with my partner.	Commitment Inventory	Stanley & Markman (1992)
Perceived Partner Regard	My partner feels affection for me.	Barrett-Lennard Relationship Inventory (OS-40)	Barrett-Lennard (1981)
	My partner respects me.	Barrett-Lennard Relationship Inventory (OS-40)	Barrett-Lennard (1981)
	My partner finds me rather dull and uninteresting. (-)	Barrett-Lennard Relationship Inventory (OS-40)	Barrett-Lennard (1981)
	My partner just tolerates or puts up with me. (-)	Barrett-Lennard Relationship Inventory (OS-40)	Barrett-Lennard (1981)
	I feel that my partner disapproves of me (-)	Barrett-Lennard Relationship Inventory (OS-40)	Barrett-Lennard (1981)
	My partner lacks respect for me. (-)	Marital Satisfaction Scale (MSS)	Roach, Frazier, & Bowden (1981)
	My partner regards me as an equal.	Marital Satisfaction Scale (MSS)	Roach, Frazier, & Bowden (1981)

Perceived Partner Satisfaction	My partner thinks we have a better relationship than most couples s/he knows.	Marital Satisfaction Scale (MSS) (adapted)	Blum & Mehrabian (1999)
	My partner is very happy with our relationship.	Perceived Relationship Quality Component (PRQC)	Fletcher, Simpson, & Thomas (2000)
	My partner thinks our relationship is strong.	Couples Satisfaction Index (CSI-16) (adapted)	Funke & Rogge (2007)
	My partner thinks we make a good team.	--	New
Perceived Similarity	My partner and I have similar ambitions and goals.	Marital Satisfaction Scale (MSS)	Blum & Mehrabian (1999)
	My partner and I agree on how we handle our finances.	Marital Satisfaction Scale (MSS)	Blum & Mehrabian (1999)
	My partner and I agree on our dealings with our in-laws.	Marital Satisfaction Scale (MSS)	Blum & Mehrabian (1999)
	My partner and I differ on our general values and beliefs. (-)	Marital Satisfaction Scale (MSS)	Blum & Mehrabian (1999)
	My partner and I often agree about major decisions.	Marital Satisfaction Scale (MSS)	Blum & Mehrabian (1999)
	My partner and I share the same basic philosophy of life.	Marital Satisfaction Scale (MSS)	Blum & Mehrabian (1999)
	I feel very good about how we each practice our religious beliefs and values.	ENRICH Marital Satisfaction Scale (EMS)	Fowers & Olson (1993)
	We agree on how children should be raised.	Relationship Satisfaction scale (RS)	Roysamb, Vitterso, & Tambs (2014)
	I share in many of my partner's interests.	Personal Assessment of Intimacy in Relationships (PAIR)	Schaefer & Olson (1981)
	My partner and I don't have much in common to talk about. (-)	Marital Satisfaction Inventory (MSI)	Snyder (1979)
	My partner and I agree on career decisions.	Dyadic Adjustment Scale (DAS)	Spanier (1976)
	My partner and I agree on household tasks.	Dyadic Adjustment Scale (DAS)	Spanier (1976)
Perceived Partner Commitment	I think my partner feels trapped in our relationship. (-)	Single item from "Conflict in the development of close relationships"	Braiker & Kelley (1979)
	I think my partner is dedicated to our relationship.	Perceived Relationship Quality Component (PRQC)	Fletcher, Simpson, & Thomas (2000)
	I think my partner is committed to maintaining our relationship.	Investment Model Scale (adapted)	Rusbult, Martz, & Agnew (1998)
	My partner wants our relationship to last forever.	Investment Model Scale (adapted)	Rusbult, Martz, & Agnew (1998)
	I think my partner is unfaithful.	--	New
Role Fulfillment	I know what my partner expects of me in our relationship.	Marital Satisfaction Scale (MSS)	Roach, Frazier, & Bowden (1981)
Sex	My partner and I disagree on sexual matters. (-)	Marital Satisfaction Scale (MSS)	Blum & Mehrabian (1999)
	My relationship is passionate.	Perceived Relationship Quality Component (PRQC)	Fletcher, Simpson, & Thomas (2000)
	My partner is very sensitive to my sexual needs and desires.	Index of Sexual Satisfaction (ISS)	Hudson, Harrison, & Crosscup (1981)
	My partner enjoys our sex life.	Index of Sexual Satisfaction (ISS)	Hudson, Harrison, & Crosscup (1981)
	I am able to tell my partner when I want sexual intercourse.	Personal Assessment of Intimacy in Relationships (PAIR)	Schaefer & Olson (1981)
	I feel our sexual activity is just routine. (-)	Personal Assessment of Intimacy in Relationships (PAIR)	Schaefer & Olson (1981)
	My partner seems disinterested in sex. (-)	Personal Assessment of Intimacy in Relationships (PAIR)	Schaefer & Olson (1981)
	I am satisfied with our sexual relationship.	Quality of Sex Inventory (QSI)	Shaw & Rogge (2016)
	I do not enjoy sexual activity with my partner. (-)	Quality of Sex Inventory (QSI)	Shaw & Rogge (2016)
	My sex life is fulfilling.	Quality of Sex Inventory (QSI)	Shaw & Rogge (2016)
	My sex life is very exciting.	Quality of Sex Inventory (QSI)	Shaw & Rogge (2016)
	Sex is fun for my partner and me.	Quality of Sex Inventory (QSI)	Shaw & Rogge (2016)
	Sexual activity with my partner leaves me empty. (-)	Quality of Sex Inventory (QSI)	Shaw & Rogge (2016)
	My partner and I are sexually compatible.	--	New
	My partner is willing to try new things in bed.	--	New
Shared Activities	I prefer doing things without my partner. (-)	Marital Satisfaction Scale (MSS)	Blum & Mehrabian (1999)

	My partner and I agree on how to spend our leisure time.	Marital Satisfaction Scale (MSS)	Blum & Mehrabian (1999)
	My partner and I try new things together.	--	New
	I attend social events with my partner.	Friendship Network Satisfaction Scale (adapted)	Kaufman et al. (2021)
	I like to hang out with my partner	Friendship Network Satisfaction Scale (adapted)	Kaufman et al. (2021)
	I spend free time with my partner.	Friendship Network Satisfaction Scale (adapted)	Kaufman et al. (2021)
	My partner and I eat together often.	Friendship Network Satisfaction Scale (adapted)	Kaufman et al. (2021)
	My partner and I have fun together.	Friendship Network Satisfaction Scale (adapted)	Kaufman et al. (2021)
	My partner usually seems interested in doing things with me.	Perceived Partner Responsiveness Scale (PPR)	Reis et al. (2017)
	I frequently enjoy pleasant conversations with my partner.	Marital Satisfaction Scale (MSS)	Roach, Frazier, & Bowden (1981)
	We enjoy the same recreational activities.	Personal Assessment of Intimacy in Relationships (PAIR)	Schaefer & Olson (1981)
	We like playing together.	Personal Assessment of Intimacy in Relationships (PAIR)	Schaefer & Olson (1981)
	We make time to do fun things together.	Personal Assessment of Intimacy in Relationships (PAIR)	Schaefer & Olson (1981)
	My partner and I engage in outside interests together.	Dyadic Adjustment Scale (DAS)	Spanier (1976)
	My partner and I laugh together.	Dyadic Adjustment Scale (DAS)	Spanier (1976)
	My partner and I work together on projects.	Dyadic Adjustment Scale (DAS)	Spanier (1976)
Socializing with Friends	My partner and I like to hang out with our friends together.	--	New
	Many of my partner's closest friends are also my closest friends.	Personal Assessment of Intimacy in Relationships (PAIR)	Schaefer & Olson (1981)
	My partner disapproves of some of my friends. (-)	Personal Assessment of Intimacy in Relationships (PAIR)	Schaefer & Olson (1981)
	We enjoy spending time with other couples.	Personal Assessment of Intimacy in Relationships (PAIR)	Schaefer & Olson (1981)
	We have very few friends in common. (-)	Personal Assessment of Intimacy in Relationships (PAIR)	Schaefer & Olson (1981)
Support	My partner cares for me.	Barrett-Lennard Relationship Inventory (OS-40)	Barrett-Lennard (1981)
	My partner is supportive of me when I have problems.	Frequency and Acceptability of Partner Behavior (adapted)	Doss & Christensen (2006)
	My partner celebrates my good news.	Friendship Network Satisfaction Scale (adapted)	Kaufman, Perez, Reise, Bradbury, & Karney (2021)
	My partner supports my career goals.	--	New
Trust	I feel that my partner is genuine with me.	Barrett-Lennard Relationship Inventory (OS-40)	Barrett-Lennard (1981)
	I always confide in my partner.	Marital Satisfaction Scale (MSS)	Blum & Mehrabian (1999)
	There are times when my partner is dishonest with me. (-)	Frequency and Acceptability of Partner Behavior (adapted)	Doss & Christensen (2006)
	My partner is dependable.	Perceived Relationship Quality Component (PRQC)	Fletcher, Simpson, & Thomas (2000)
	There are times when my partner cannot be trusted. (-)	Dyadic Trust Scale	Larzelere & Huston (1980)
	I can always trust my partner.	Marital Satisfaction Scale (MSS)	Roach, Frazier, & Bowden (1981)

5. Study 2 Review of Satisfaction/Relationship Quality Measures for Item Selection

As noted in the manuscript, a potential critique of Study 1 was that certain constructs were represented by an insufficient number of (prototypical) items (e.g., few items were coded as measures of trust, and these items also may not align well with widely used relationship trust

scales). Prior to Study 2, a review was conducted by members of the research team (specifically the 1st, 2nd, 4th, and 8th authors). The top measures in the field were reviewed (in consideration of those already encompassed in Study 1) in order to select the 408 prototypical items representing 34 of the most prominent relationship constructs in Study 2. In particular, these relationship constructs were selected based on research identifying the most frequently studied measured constructs in the field (Joel et al., 2020). Item indicators for each construct were selected from established measures based on their prevalence in the literature (i.e., selecting measures with the highest citation count), and based on the length of the measure (i.e., favoring shorter versus longer measures when measures were roughly similar in terms of their assumed validity and usage in the field). Items were also selected based on their overlap with the 206 items from Study 1, such that we favored measures with items already included in Study 1. In other words, we sought to expand upon the items already included to encompass as many constructs as possible, while also striving to ensure there were a minimally sufficient number of items per construct (i.e., three indicators; Marsh et al., 1998). We also attempted to include additional items so that the collective set of items for a construct could be reasonably said to represent a complete validated measure of that construct (rather than including only one or two items from various different scales to measure a given construct). Table S5 below provides a full list of the constructs examined, the source of the items of each construct, and number of item indicators selected across both studies.

The 34 constructs were determined by first taking the best predictors of relationship quality listed in Table 2 of Joel et al. (2020). This consisted of 29 constructs (including satisfaction and commitment). From this list of 29 constructs, we omitted four as they are single-indicator demographic variables (sexual frequency, relationship status, cohabiting, children).

Then, five additional constructs were included by the authors to ensure major relationship theories listed in Eastwick & Finkel (2018) were represented. Specifically, we included communal strength (communal theory), relationship-specific attachment (attachment theory), self-disclosure (interpersonal process of intimacy), goal compatibility (transactive goal dynamics theory), and partner traits (ideal standards model).

To select measures and items across these constructs, our approach was as follows:

1. We first identified the few most prominent measures for each of the 34 constructs based on citation count and usage in the datasets of Joel et al. (2020). We then sought to determine whether items from these measures were already represented (at least to some degree) in the 206 items of Study 1. For many of the constructs, there was a clear representative measure based on its total citation count and citation count over the past 10 years. See Table S2 for the commonly-used measures for each construct that were considered based on citation count and overlap with the item pool in Study 1.
2. In line with the item selection criteria overviewed earlier, we next identified how many items in Study 1 were already represented (i.e., had strong content overlap) within the prominent measures identified across the 34 constructs (e.g., a satisfaction measure, X, contains the item “Y” which is similar to the item “Y2” in the formal measure of Z). Table S4 indicates how many items from top measures for each construct were included in Study 1 and Study 2.
3. Finally, we included items from the most prominent measures across constructs, striving to ensure each construct is represented by at least six items per construct (with the exception of IOS, commonly measured as a 1-item figure). See

supplemental document. An excel document in the OSF (S1 and S2 Item Information.xlsx) provides information about all final items selected and their original source, as well as any modifications made to items relative to the original source.

5.1 Table S5. *Study 2 focal measures selection*

Study 2 Selected Construct (34 total)	Citation #	Citation # past 10 yrs	Common Measure/s Considered Based on Citation Count and S1 Item Pool Overlap	Reference	Does S1 include scale items from this measure?	Measure selected for inclusion in S2 item pool?
**Affection (& Perceived Partner Affection)**	516	223	Socioemotional Behavior index	(Huston & Vangelisti, 1991)	N	Y
**Appreciation (& Perceived Partner Affection)**	331	315	Appreciation in Relationships (AIR)	(Gordon et al., 2012)	Y	Y
**Capitalization**	1676	1340	Perceived Responses to Capitalization Attempts Scale	(Gable et al., 2018)	N	Y
**Commitment**	2760	1990	Investment Model Scale	(Rusbult, Martz, & Agnew, 1998)	Y	Y
	1126	857	PRQC	(Fletcher, Simpson, & Thomas, 2000)	Y	Y
	1055	621	Commitment Inventory	Stanley & Markman (1992)	Y	N
**Communal Strength**	325	234	Communal Strength Scale	(Mills et al., 2004)	Y	Y
**Conflict Frequency**	320	190	Marital Satisfaction Scale (CMSS) - 1 item	(Blum & Mehrabian, 1999)	Y	Y
	103	103	Conflict Frequency Items	(Gordon & Chen, 2016)	N	Y
	1228	451		(Braiker & Kelley, 1979)	Y	N
**Conflict strategies & Communication**	1424	586	Communication patterns questionnaire Short (CPQ-SF)	(Christensen & Heavey, 1990)	Y	Y
	566	349	Conflict Resolution Styles Inventory (CRSI)	(Kurdek, 1994)	N	N
	538	271	Kerig Conflict Strategies	(Kerig, 1996)	N	N
	245	77	Conflict Resolution Behavior	(Rands, Levinger, & Mellinger, 1981)	N	N
	666	480	Dutch Test for Conflict Handling	(De Dreu et al., 2001)	N	N
**Empathy**	178	170	Interpersonal Reactivity Index for Couples	(Péloquin & Lafontaine, 2010)	N	Y
**Goal Compatibility**	320	190	Marital Satisfaction Scale (CMSS)	(Blum & Mehrabian, 1999)	Y	Y

	11147	4450	Dyadic Adjustment Scale (DAS)	(Spanier, 1976)	Y	N
**Inclusion of Other in Self**	5558	4120	Inclusion of Other in Self Scale	(Aron, Aron, & Smollan, 1992)	N	Y
**Intimacy & Domain-specific Intimacy**	1268	556	Personal Assessment of Intimacy in Relationships (PAIR)	(Schaefer & Olson, 1981)	Y	Y
	1126	857	PRQC	(Fletcher, Simpson, & Thomas, 2000)	Y	Y
	254	82	Marital Intimacy Questionnaire	(Waring & Reddon, 1983)	N	N
**Intimate Partner Violence**	850	741	Conflict Tactics Scale Revised (CTS2) Short Form	(Straus & Douglas, 2004)	N	N
	8266	5390	Conflict Tactics Scale - Revised	(Straus et al., 1996)	N	Y
**Investment**	2760	1990	Investment Model Scale	(Rusbult, Martz, & Agnew, 1998)	Y	Y
**Love**	259	158	Passionate and Companionate Love Scale	(Sprecher & Regan, 1998)	Y	Y
	1126	857	PRQC	(Fletcher, Simpson, & Thomas, 2000)	Y	Y
	1685	792	Love Attitudes Scale	(Hendrick & Hendrick, 1986)	N	N
	1088	793	Sternberg Triangular Love Scale	(Sternberg, 1997)	N	N
	2135	797	Rubin Love Scale	(Rubin, 1970)	N	N
**Normative Attachment**	171	123	Attachment Features and Functions	(Tancredy & Fraley, 2006)	N	Y
**Partner Traits**	812	537	Positive Ideals Scale	(Fletcher, Simpson, Thomas, & Giles, 1999)	N	Y
**Passion**	259	158	Passionate and Companionate Love Scale	(Sprecher & Regan, 1998)	Y	Y
	1126	857	PRQC	(Fletcher, Simpson, & Thomas, 2000)	Y	Y
**Perceived Partner Commitment**	173	139	(adapted) Investment Model Scale	(Arriaga et al., 2006)	Y	Y
	1126	857	(adapted) PRQC	(Fletcher, Simpson, & Thomas, 2000)	Y	N
**Perceived Partner Responsiveness**	275	263	Perceived Partner Responsiveness S cale (PPRS)	(Reis et al., 2011)	Y	Y
**Perceived Partner Satisfaction**	2760	1990	(adapted) Investment Model Scale (2 subscales)	(Rusbult, Martz, & Agnew, 1998)	N	Y
	1126	857	(adapted) PRQC	(Fletcher, Simpson, & Thomas, 2000)	Y	N
	1813	1700	(adapted) Couples Satisfaction Index	(Funk & Rogge, 2007)	Y	N
	320	190	(adapted) Comp. Marital Satisfaction Scale (CMSS)	(Blum & Mehrabian, 1999)	Y	N
**Power**	802	786	Personal Sense of Power Scale	(Anderson, John, & Keltner, 2012)	N	Y
	57	57	Relationship Power Inventory	(Farrell, Simpson, & Rothman, 2015)	N	N
**Quality Of Alternatives**	2760	1990	Investment Model Scale	(Rusbult, Martz, & Agnew, 1998)	Y	Y

**Relationship-specific Attachment (Avoidance & Anxiety)**	1238	1210	Experiences in Close Relationships - Relationship Structures Questionnaire	(Fraley et al., 2011)	N	Y
**Sacrifice motives**	398	324	Impett Approach Avoidance Motives Scale	(Impett, Gable, & Peplau, 2005)	N	Y
**Satisfaction**	2760	1990	Investment Model Scale	(Rusbult, Martz, & Agnew, 1998)	Y	Y
	320	190	Comp. Marital Satisfaction Scale (CMSS)	(Blum & Mehrabian, 1999)	Y	Y
	1813	1700	Couples Satisfaction Index (CSI-16)	(Funk & Rogge, 2007)	Y	N
	2870	1890	Relationship Assessment Scale (RAS)	(Hendrick, 1988)	Y	N
	11147	4450	Dyadic Adjustment Scale (DAS)	(Spanier, 1976)	Y	N
	695	519	ENRICH Marital Satisfaction Scale (EMS)	(Fowers & Olson, 1993)	Y	N
	1168	542	Kansas Marital Satisfaction Scale (KMS)	(Schumm et al., 1986)	Y	N
	477	216	Marital Satisfaction Scale (MSS)	(Roach, Frazier, & Bowden, 1981)	Y	N
	11147	4450	Dyadic Adjustment Scale (DAS)	(Spanier, 1976)	Y	N
	2389	1330	Quality of Marriage Index (QMI)	(Norton, 1983)	Y	N
**Self-disclosure**	801	339	self-disclosure index	(Miller, Berg, & Archer, 1983)	N	Y
**Sexual satisfaction**	47	47	Quality of Sex Inventory (QSI)	(Shaw & Rogge, 2016)	Y	Y
	484	275	Index of Sexual Satisfaction (ISS)	(Hudson, Harrison, & Crosscup, 1981)	Y	N
	961	734	GMSEX	(Lawrance & Byers, 1995)	N	N
	344	334	New Sexual Satisfaction Scale	(Štulhofer, Buško, & Brouillard, 2010)	N	N
**Social Support**	1003	473	Quality of Relationships Inventory (subscale)	(Pierce, Sarason, & Sarason, 1991)	Y	Y
	521	195	Social Support Behaviors Scale	(Vaux, Riedel, & Stewart, 1987)	N	Y
**Trust**	2050	989	Dyadic Trust Scale	(Larzelere & Huston, 1980)	Y	Y
	1126	857	PRQC	(Fletcher, Simpson, & Thomas, 2000)	Y	Y
	5142	2660	Trust in Close Relationships Scale	(Rempel, Holmes, & Zanna, 1985)	N	N
	5815	3050	Rotter Interpersonal Trust Scale	(Rotter, 1967)	N	N

5.2 Table S6. *Study 2 item selection information (constructs, sources, and citation count, and number of items included)*

Construct	Measure	Source	Items Present in S1	Items Added to S2	Citation #
Affection	Comp. Marital Satisfaction Scale (CMSS)	Blum & Mehrabian (1999)	3	-	320
	Dyadic Adjustment Scale (DAS)	Spanier (1976)	1	-	10,915
	Socioemotional Behavior Index (SBI)	Huston & Vangelisti (1991)	-	7	552
Affection (Perceived Ptr)	Barrett-Lennard Relationship Inventory (OS-40)*	Barrett-Lennard (1981)	3	-	2154
	Frequency and Acceptability of Partner Behavior*	Doss & Christensen (2006)	3	-	33
Appreciation	Appreciation in Relationships Scale (AIR)	Gordon et al. (2012)	4	5	331
	Face-Valid	NA	1	-	NA
Appreciation (Perceived Ptr)	Appreciation in Relationships Scale (AIR)	Gordon et al. (2012)	5	-	331
	Barrett-Lennard Relationship Inventory (OS-40)	Barrett-Lennard (1981)	7	-	2154
	Face-Valid	NA	1	-	NA
	Marital Satisfaction Scale (MSS)	Roach, Frazier, & Bowden (1981)	1	-	477
Attachment Anxiety	Experiences in Close Relationships - Relationship Structures Questionnaire (ECR-RS)	Fraley et al. (2011)	-	3	1238
	Experiences in Close Relationships - Short Form (ECR-S)*	Wei et al. (2007)	-	2	1670
	Marital Satisfaction Scale (MSS)	Roach, Frazier, & Bowden (1981)	1	-	477
Attachment Avoidance	Experiences in Close Relationships - Relationship Structures Questionnaire (ECR-RS)	Fraley et al. (2011)	-	6	1238
Capitalization	Perceived Responses to Capitalization Attempts Scale	Gable et al., 2004	-	12	1676
Commitment	Alone Together Scale	Amato, Booth, Johnson, & Rogers (2007)	2	-	849
	Commitment Inventory	Stanley & Markman (1992)	2	-	1055
	Dyadic Adjustment Scale (DAS)	Spanier (1976)	1	-	10,915
	Investment Model Scale	Rusbult, Martz, & Agnew (1998)	3	4	2760
	Marital Satisfaction Inventory (MSI)	Snyder (1979)	1	-	309
	Marital Satisfaction Scale (MSS)	Roach, Frazier, & Bowden (1981)	1	-	477
	Friendship Network Satisfaction Scale*	Kaufman et al. (2021)	1	-	0
	Quality of Marriage Index (QMI)	Norton (1983)	1	-	2389
	Relationship Satisfaction Scale (RS)	Roysamb, Vitterso, & Tambs (2014)	1	-	50

Commitment (Perceived Ptr)	Frequency and Acceptability of Partner Behavior*	Doss & Christensen (2006)	1	-	33
	Alone Together Scale	Amato, Booth, Johnson, & Rogers (2007)	2	-	849
	Investment Model Scale*	Arriaga et al. (2006)	-	4	173
	Face-Valid	NA	5	-	NA
Communal Strength	Communal Strength Scale	Mills et al. (2004)	4	6	325
	Relationship Assessment Scale (RAS)	Hendrick (1988)	1	-	50
	Braiker-Kelley Partnership Questionnaire	Braiker & Kelley (1979)	2	-	1194
	Comp. Marital Satisfaction Scale (CMSS)	Blum & Mehrabian (1999)	2	-	320
	ENRICH Marital Satisfaction Scale (EMS)	Fowers & Olson (1993)	1	-	695
	Friendship Network Satisfaction Scale*	Kaufman et al. (2021)	1	-	0
Conflict Frequency	Braiker-Kelley Partnership Questionnaire	Braiker & Kelley (1979)	1	-	1194
	Comp. Marital Satisfaction Scale (CMSS)	Blum & Mehrabian (1999)	1	-	320
	Conflict Frequency Measure	Gordon & Chen, 2016	-	6	103
	Dyadic Adjustment Scale (DAS)	Spanier (1976)	1	-	10,915
	Marital Satisfaction Inventory (MSI)	Snyder (1979)	2	-	309
	Relationship Satisfaction Scale (RS)	Roysamb, Vitterso, & Tambs (2014)	1	-	50
Conflict Strategies	Frequency and Acceptability of Partner Behavior*	Doss & Christensen (2006)	1	-	33
	Communication Patterns Questionnaire Short (CPQ-SF)	Christensen & Heavey (1990)	6	6	1424
	Communication Patterns Questionnaire (CPQ)	Christensen & Sullaway (1984)	6	-	455
	Comp. Marital Satisfaction Scale (CMSS)	Blum & Mehrabian (1999)	1	-	320
	Marital Satisfaction Scale (MSS)	Roach, Frazier, & Bowden (1981)	3	-	477
Empathy	Interpersonal Reactivity Index for Couples Short	Peloquin & Lafontaine (2010)	-	10	170
Goal Compatibility	Comp. Marital Satisfaction Scale (CMSS)	Blum & Mehrabian (1999)	9	-	320
	Dyadic Adjustment Scale (DAS)	Spanier (1976)	4	-	10,915
	ENRICH Marital Satisfaction Scale (EMS)	Fowers & Olson (1993)	1	-	695
	Friendship Network Satisfaction Scale*	Kaufman et al. (2021)	1	-	0
	Relationship Satisfaction Scale (RS)	Roysamb, Vitterso, & Tambs (2014)	1	-	50
Inclusion of Other in Self	Inclusion of Other in Self Measure	Aron et al. (1992)	-	1	5558
Intimacy	Marital Satisfaction Inventory (MSI)	Snyder (1979)	1	-	309
	Marital Satisfaction Scale (MSS)	Roach, Frazier, & Bowden (1981)	2	-	477
	Friendship Network Satisfaction Scale*	Kaufman et al. (2021)	9	-	0

	Perceived Relationship Quality Components Scale (PRQC)	Fletcher, Simpson, & Thomas (2000)	-	3	1126
	Quality of Marriage Index (QMI)	Norton (1983)	1	-	2389
	Relationship Satisfaction Scale (RS)	Roysamb, Vitterso, & Tambs (2014)	1	-	50
Intimacy (domain-specific)	Personal Assessment of Intimacy in Relationships (PAIR)	Schaefer & Olson (1981)	18	-	1246
Intimate Partner Violence	Conflict Tactics Scale Revised - Short	Straus & Douglas (2004)	-	16	850
Investment	Braiker-Kelley Partnership Questionnaire	Braiker & Kelley (1979)	1	-	1194
	Investment Model Scale	Rusbult, Martz, & Agnew (1998)	3	5	2661
	Face-Valid	NA	2	-	NA
Love	Companionate Love Scale	Sprecher & Regan (1998)	-	7	259
	Perceived Relationship Quality Components Scale (PRQC)	Fletcher, Simpson, & Thomas (2000)	2	1	1126
Normative Attachment	Attachment Features and Functions Scale*	Tancredy & Fraley (2006)	-	8	171
	Braiker-Kelley Partnership Questionnaire	Braiker & Kelley (1979)	1	-	1194
Partner Traits	Alone Together Scale	Amato, Booth, Johnson, & Rogers (2007)	7	-	849
	Comp. Marital Satisfaction Scale (CMSS)	Blum & Mehrabian (1999)	1	-	320
	Marital Satisfaction Scale (MSS)	Roach, Frazier, & Bowden (1981)	1	-	477
	Positive Ideals Scale	Fletcher et al. (1999)	-	12	812
Passion	Passionate Love Scale	Sprecher & Regan (1998)	-	10	259
	Perceived Relationship Quality Components Scale (PRQC)	Fletcher, Simpson, & Thomas (2000)	1	2	1126
Perceived Partner Responsiveness	Perceived Partner Responsiveness Scale (PPRS)*	Reis et al. (2011)	4	8	275
	Relationship Satisfaction Scale (RS)	Roysamb, Vitterso, & Tambs (2014)	1	-	50
Power (felt and desired)	Feeling Powerful and Desiring Power Scales	Murphy et al. (2022)	-	6	0
	Personal Sense of Power Scale	Anderson, John, & Keltner (2012)	-	8	802
Quality of Alternatives	Investment Model Scale	Rusbult, Martz, & Agnew (1998)	-	6	2661
	Marital Satisfaction Scale (MSS)	Roach, Frazier, & Bowden (1981)	1	-	477
Sacrifice Motives	Approach and Avoidance Motives Scale	Impett et al. (2013)	-	8	398
Satisfaction	Braiker-Kelley Partnership Questionnaire	Braiker & Kelley (1979)	1	-	1194
	Comp. Marital Satisfaction Scale (CMSS)	Blum & Mehrabian (1999)	2	-	320
	Couples Satisfaction Index (CSI-16)	Funk & Rogge (2007)	3	-	1813
	ENRICH Marital Satisfaction Scale (EMS)	Fowers & Olson (1993)	2	-	695
	Investment Model Scale	Rusbult, Martz, & Agnew (1998)	1	-	2760

	Kansas Marital Satisfaction Scale (KMS)	Schumm et al., 1986	1	-	1168
	Marital Satisfaction Inventory (MSI)	Snyder (1979)	1	-	309
	Marital Satisfaction Scale (MSS)	Roach, Frazier, & Bowden (1981)	8	-	477
	Positive and Negative Semantic Differential (PN-SMD)	Mattson et al. (2013)	4	-	109
	Quality of Marriage Index (QMI)	Norton (1983)	1	-	2389
	Relationship Assessment Scale (RAS)	Hendrick (1988)	1	-	2870
Satisfaction (Perceived Ptr)	Investment Model Scale*	Rusbult, Martz, & Agnew (1998)	-	5	2661
	Face-Valid	NA	4	-	NA
Self-Disclosure	Self-Disclosure Index	Miller, Berg, & Archer, 1983	-	10	801
	Comp. Marital Satisfaction Scale (CMSS)	Blum & Mehrabian (1999)	1	-	320
Sexual Satisfaction	Index of Sexual Satisfaction (ISS)	Hudson, Harrison, & Crosscup (1981)	2	-	484
	Quality of Sex Inventory*	Shaw & Rogge (2016)	6	8	47
	Face-Valid	NA	2	-	NA
Social Support	Frequency and Acceptability of Partner Behavior*	Doss & Christensen (2006)	1	-	33
	Quality of Relationships Inventory (QRI subscale)	Pierce et al. (1991)	-	7	1003
Trust	Dyadic Trust Scale	Larzelere & Huston (1980)	4	-	2005
	Marital Satisfaction Scale (MSS)	Roach, Frazier, & Bowden (1981)	2	-	477
	Perceived Relationship Quality Components Scale (PRQC)	Fletcher, Simpson, & Thomas (2000)	1	2	1126

Note. Measures with an asterisk indicates that at least some of the items were adapted for inclusion in study.

6. Additional Information for Results of Study 2 Replication Analyses

Results from exploratory factor analyses replicated the results from Study 1 (see output for ‘S2.analyses_OSF’). EFA metrics suggested various potential factor solutions across selection criteria. Scree plot and hierarchical cluster analysis suggested 2 factors (comprised of 169 and 37 items, with eigenvalues indicating a very large first factor compared to subsequent extracted factors), MAP and BIC suggested 11 factors, SABIC suggested 20 factors, and parallel analyses suggested 32 factors. Thus, we estimated EFA models probing each of these factor structures across three different extraction methods (which yielded similar results) and applied our

evaluation criteria (i.e., primary loading >.30 and cross-loadings <.30) to interpret the viability of each potential factor solution. Aligning with Study 1 findings, a two-factor model was represented by a positive factor and negative factor, (43% variance explained) whereby the strongest loading items on the positive factor were sexual satisfaction items. Solutions for 11, 20, and 32 factors were not readily interpretable due to several factors having limited item representation and/or weak loadings. For example, in the 11-factor solution, 5 of the factors were not represented by any items, and one factor was represented by less than 3 items. The pattern of item loadings across these higher factor solutions showed that these structures were only interpretable up to their first 4 factors; however, the same 4 factors emerged consistently within these models. On the basis of our EFA results, and in consideration of EFA findings from Study 1 supporting a 3-factor structure, we additionally examined 3- and 4-factor solutions. Examination of the 3-factor solution was represented by a positive factor, a negative factor, and a sex factor, consistent with Study 1 (45% variance explained). A similar structure was obtained for the 4-factor solution (47% variance explained), except the positive factor split into a factor primarily characterized by indicators reflecting ‘positive partner evaluations’ (i.e., perceived partner responsiveness) and ‘emotional attachment’ (e.g., indicators of love and commitment). Taken together, the broad pattern of findings from EFA were highly consistent with the content of dimensions identified in Study 1. (See supplemental tables for item loading matrices of all factor models).

Given that the optimal number of factors (2, 3, or 4) wasn't clearly evident in EFA models, we explored each of these possibilities in subsequent EBFA analysis by evaluating bifactor models with 2, 3, and 4 specific factors. Across these models, we evaluated omegaH and ECV indices to quantify the strength of a general Q-factor explaining the common variance

across all items, and omegaHS and ECV values to assess the unique contribution of specific factors as substantive dimensions after partitioning out variability attributed to the general factor (Rodriguez et al., 2016b). EBFA models did not converge beyond solutions with six specific factors, consistent with the indeterminacy of the higher-factor solutions identified in EFA.

Table S7 presents the bifactor indices for the solution with 3 specific factors with the same 206 items in Study 1. As in Study 1, OmegaH was high (.84), indicating a strong influence of a general factor. ECV for the general factor was also high (.83), suggesting the common variance across items is essentially unidimensional (Rodriguez et al., 2016a). In turn, omega hierarchical estimates for specific factors (i.e., OmegaHS) were very low for all specific group factor scores across all solutions (OmegaHS range = .00 – .11), as were ECV_{SS} values for specific factors (range = .04 – .09). Thus, consistent with Study 1, these findings indicated that, regardless of whether we specified two, three, or four specific factors, the specific factors captured no meaningful variance over and above the influence of a single general factor representing global relationship assessments.

6.1 Table S7. *Study 2 EBFA 206-item Re-analysis: Top 10 Factor Loadings and Bifactor Indices*

	Bifactor Model (Q + 3 specific factors)			
Item	General (Q)	SF1	SF2	SF3
My relationship with my partner is enjoyable.	**0.84**	0.06	0.13	-0.06
All things considered, I am very happy in my relationship with my partner.	**0.82**	0.02	0.13	-0.12
My relationship with my partner is rewarding.	**0.83**	0.01	0.10	-0.07
My relationship with my partner is strong.	**0.82**	-0.02	0.09	-0.07
I have a close relationship with my partner.	**0.82**	0.00	0.09	-0.02
My partner respects me.	**0.83**	-0.10	-0.07	-0.15
I know I’m valued and appreciated by my partner.	**0.82**	-0.02	-0.08	-0.14
My partner is generally understanding.	**0.82**	-0.06	-0.07	-0.11
I have a warm and comfortable relationship with my partner.	**0.84**	-0.02	0.05	-0.11
My partner makes me feel special.	**0.82**	0.01	-0.04	-0.04
I want our relationship to last a very long time.	0.67	**0.44**	0.01	-0.03

I love my partner.	0.60	**0.44**	0.08	0.04
I want to grow old with my partner.	0.67	**0.40**	-0.01	-0.01
I want this relationship to stay strong no matter what rough times we may encounter.	0.61	**0.37**	0.03	-0.03
It is hard to imagine my life without my partner.	0.62	**0.35**	0.00	0.05
I am committed to maintaining my relationship with my partner.	0.65	**0.34**	0.01	-0.03
I would go out of my way to do something for my partner.	0.51	**0.32**	0.02	0.04
I would enjoy living apart from my partner.	-0.53	**-0.29**	0.20	0.04
I adore my partner.	0.70	**0.27**	0.07	0.12
I feel happy when I do something that helps my partner.	0.54	**0.27**	0.04	0.10
When we have problems, I call my partner names, swear at them, or attack their character.	-0.31	-0.07	**0.49**	0.01
My partner and I argue with each other often.	-0.44	0.01	**0.49**	0.01
When we have problems, my partner calls me names, swears at me, or attacks my character.	-0.54	-0.06	**0.47**	0.10
Minor disagreements with my partner often end up in big arguments.	-0.53	0.06	**0.46**	-0.04
During a discussion of a relationship issue or problem, my partner and I blame, accuse, and criticize one another.	-0.54	0.05	**0.46**	-0.02
When we have problems, my partner and I threaten one another with negative consequences.	-0.50	-0.02	**0.45**	0.01
My partner gets angry easily.	-0.43	0.06	**0.45**	-0.04
My partner and I often argue about finances.	-0.43	-0.05	**0.43**	0.04
I worry a lot about my relationship with my partner.	-0.44	0.17	**0.42**	0.04
My partner and I often get on each other's nerves.	-0.52	0.00	**0.41**	-0.15
My sex life with my partner is very exciting.	0.55	0.06	0.02	**0.60**
My sex life with my partner is fulfilling.	0.59	0.07	-0.03	**0.55**
Sex is fun for my partner and I.	0.65	0.15	0.01	**0.52**
I am satisfied with our sexual relationship.	0.60	0.00	-0.03	**0.51**
My partner and I are sexually compatible.	0.55	0.19	0.04	**0.49**
My partner enjoys our sex life.	0.57	-0.04	-0.03	**0.44**
My partner is willing to try new things in bed.	0.43	-0.02	0.12	**0.40**
My partner seems disinterested in sex.	-0.44	0.03	0.08	**-0.40**
I do NOT enjoy sexual activity with my partner.	-0.50	-0.30	0.04	**-0.39**
My partner and I disagree on sexual matters.	-0.44	-0.06	0.17	**-0.38**
	Bifactor Indices			
	ω_H	ω_{HS}	ω_{HS}	ω_{HS}
	.84	.11	.00	.01
	ECV	ECV_{SS}	ECV_{SS}	ECV_{SS}
	.83	.09	.04	.05

Note. Factor loadings in bold designate the strongest loading items for each general and specific factor.

7. Additional Information for Results of Study 2 Expansion Analyses

Results from exploratory factor analyses with the broader item set (i.e., 408 items) are provided in the output for 'S2.analyses_OSF'. Scree plot and eigenvalues suggested 1 large cluster, while hierarchical cluster analysis indicated 3 or 5 additional smaller components comprised of 9-12 items each. SABIC suggested 10 factors, BIC suggested 11 factors, parallel analyses using different extraction methods suggested between 15-17 factors, and MAP suggested 17 factors. Thus, EFAs were conducted for each of these factor solutions, in addition to exploring a bifactor model with two and three specific factors based on previous findings. Applying different extraction methods across these solutions (i.e., 1, 2, 3, 4, 6, 11, 15, 16, 17) again yielded similar results.

A one-factor solution was best represented by items capturing positive global evaluations of the relationship and/or partner. Similar to the 206-item analyses, a two-factor model was represented by a positive factor and negative factor, with sexual satisfaction items representing the strongest loading items on the positive factor, and partner-specific attachment anxiety and conflict items representing the negative factor. In the three-factor model, the first factor was now best represented by positive global partner evaluation items, the second was represented by negative evaluations in the form of relational dominance variables (e.g., desire for power over partner, intimate partner violence), and the third factor was now primarily represented by sexual satisfaction. In the four-factor model, each of the three factors were retained, with the fourth consisting of the emotional attachment factor (i.e., love and commitment items).

Factor solutions for five factors and greater (i.e., 5, 6, 11, 15, 16, 17) were not readily interpretable due to several factors having limited item representation and/or weak loadings.

Furthermore, we note that EBFA models did not converge when specifying more than five specific factors. An EBFA model specifying four specific factors showed that only one item

loaded (weakly) onto the fourth specific factor, leaving this solution uninterpretable. Thus, consistent with prior results drawn from the 206 items, a bifactor structure with three specific factors was retained for subsequent analyses. The top loading items for the three-factor EFA model are presented in Table S8.

7.1 Table S8. *Top Loading Items for the 3-factor EFA Model in Study 2 (408 items)*

Item	F1 Pos	F2 Neg	F3 Sex
My partner respects me.	**.78**	.07	.07
My partner regards me as an equal.	**.75**	.02	.04
I know I'm valued and appreciated by my partner.	**.75**	.08	.05
My partner makes sure I feel appreciated.	**.73**	.10	.12
My partner values my abilities and opinions.	**.73**	.04	.14
My partner gives me sufficient opportunity to express my opinions.	**.73**	.06	.09
I can get my partner to listen to what I say.	**.73**	.03	.08
My partner thinks our relationship is strong.	**.72**	.01	.09
My partner is generally understanding.	**.71**	.13	.09
I would enjoy having authority over my partner.	.20	**.68**	-.04
I work to control my partner more than they control me.	.27	**.65**	-.16
I try to have more influence than my partner.	.25	**.63**	-.16
I have a strong drive to get power in my romantic relationship.	.16	**.60**	.16
I like to tell my partner what they should do.	.21	**.58**	-.04
In our relationship, my partner often uses force (like hits, holds me down, or uses a weapon) to make me have sex.	.07	**.43**	-.08
In our relationship, I often use force (like hitting, holding down, or using a weapon) to make my partner have sex.	.18	**.42**	-.13
When I tell my partner about something good that has happened to me, my partner reminds me that most good things have their bad aspects as well.	-.13	**.41**	.05
The people other than my partner with whom I might become involved with are very appealing.	.10	**.40**	-.18
My partner is sexy.	-.09	-.04	**.67**
Sexual activity with my partner is rewarding.	.07	.02	**.62**
Sexual activity with my partner is fantastic.	.14	.11	**.59**
I want my partner physically, emotionally, mentally.	.02	-.07	**.59**
Sex is fun for my partner and I.	.15	.06	**.59**
My partner is attractive.	-.04	-.10	**.58**
I love my partner.	-.02	-.18	**.57**
My sex life with my partner is very exciting.	.13	.13	**.56**
I do NOT enjoy sexual activity with my partner.	.05	.10	**-.56**
I would rather be with my partner than anyone else.	.09	-.16	**.55**
	Factor Correlations		
F1	-		
F2	-.52	-	
F3	. 66	-.29	**-**

Note. Factor loadings in bold indicate the strongest loading items for each factor. See Appendix D for full pattern matrix.

8. EFA results by estimation method

Recognizing that different factor selection criteria and extraction methods could yield diverse results, our approach entailed examining all factor solutions suggested across selection methods. Further, we conducted separate analyses for each potential n-factor solution derived from EFA metrics with three different extraction methods (maximum likelihood, minimum residual, principal axis). For all EFA solutions that were initially considered in Studies 1 and 2 (see manuscript), we provide additional information regarding the top loading items for those factor solutions (i.e., which construct the item belongs to, the loading value) for each of the three estimation methods. Overall, results were highly consistent and converged across the estimation methods. This information can be viewed in the supplemental excel spreadsheet documents for Studies 1 and 2 (S1_top10_EFAs.xlsx & S2_top10_EFAs.xlsx).

9. EBFA results by rotation method

As noted in the manuscript, we relied primarily on SLiD and we report results from it throughout this manuscript. At the same time, we also ran all analyses using five other bifactor algorithms, to see whether they produced largely converging results and ensure that our conclusions were not unique to the SLiD method. Drawing on research comparing optimal EBFA analytic rotation criteria (e.g., Abad et al., 2017; Cho, 2022; Garcia-Garzon et al., 2019), we used non-iterative Schmid-Leiman methods (i.e., SL, DSL, DBF) and direct analytical bifactor rotations (i.e., Bi-quartimin, bi-geomin). Bifactor estimates (omegaH, omegaHS, and ECV values) are provided in the supplemental excel spreadsheet documents for Studies 1 and 2 (S1_EBFAresults.xlsx & S2_EBFAresults.xlsx).

We briefly outline here some of the strengths and limitations of the various exploratory bifactor rotation methods. The most well-known technique is Schmid-Leiman (SL) procedure, in which: a) a primary factor solution is extracted, b) an oblique rotation is performed, c) a higher-

order factor solution consisting of a general factor is extracted, and d) loadings are then orthogonalized into g + 1 factors comprising a general factor and g group factors. There are two primary limitations of SL. First, general factor loadings are a linear combination of group factor loadings, thus making this technique unsuitable for nonproportional data. Second, SL arbitrarily presents only one of an infinite number of linear combinations and thus may not be optimal. Four techniques have been proposed to solve these problems, and perform well in Monte Carlo simulations (Abad et al., 2017; Garcia-Garzon et al., 2021; Giordano & Waller, 2020). In direct SL (DSL) and direct bifactor (DBF) rotation, a bifactor solution is estimated using orthogonal Procrustes rotation and only one factor analysis is needed unlike the two required in SL. DSL rotates an augmented and rank-deficient facor solution to a target matrix whereas DBF consists of a rotating a full-rank loadings matrix to a target matrix (Waller, 2018). Another group of techniques consists of SL with iterative target rotation (SLi) (Abad et al., 2017; Browne, 2001; Moore et al., 2015; Reise et al., 2011) and iterative empirical target rotation (SLiD) (Garcia-Garzon et al., 2019). These techniques are free from the proportionality constraints of SL. In SLi, an initial SL solution is obtained, a target matrix is subsequently defined in which loadings below a user-specified cutoff (e.g., .20; Abad et al., 2017) are set to zero while loadings above the cutoff are unspecified, and then a target rotation is performed. After the target matrix is updated with the obtained solution, this step is repeated until a particular criterion is obtained. SLiD is analogous to SLi but uses an empirical cutoff obtained from factor loadings instead of a fixed cutoff. Nonhierarchical exploratory bifactor analysis algorithms (Jennrich & Bentler, 2011) don’t assume a strict hierarchy between the general factor and the specific factors, such that all factors are allowed to correlate freely with each other, and a general factor essentially competes with specific factors to explain variance. However, bi-geomin and bi-quartimin are prone to

shifting the variance contained in one of the group factors to the general factor and to produce factor collapse (Mansolf & Reise, 2016). Other authors have also found these methods to overestimate general factor loadings (Revelle & Wilt, 2013, p. 495). Bi-geomin is also expected to be more accurate than bi-quartimin for complex structures (i.e., ICBP; Abad et al., 2017), but not for simpler structures (ICB; Figure 2; Giordano & Waller, 2019). Simulation studies have demonstrated SLiD outperforms and provides better reliability estimation than bi-geomin, bi-quartimin methods (Abad et al., 2017; Garcia-Garzon et al., 2019; Giordano & Waller, 2019), and is able to recover complex bifactor structures better than other rotation algorithms when multiple cross-loadings and weak factors are present (Garcia-Garzon et al., 2019). Unlike the DSL (i.e., Direct Schmid-Leiman) and DBF (i.e., Direct Bi-Factor) algorithms (Waller, 2018), SLiD differs in that it does not apply a specified target rotation where all elements of the target matrix are given a value toward which loadings are maximized (±1) or minimized (0) (in DSL, a user-defined item loading threshold of .25 is applied). Based on previous studies, DSL is expected to provide adequate results when applied to recover either type of structure (i.e., full or deficient rank solutions), and to outperform DBF under many conditions (Giordano & Waller, 2019). However, DSL has been shown to provide optimal approximation to a bi-factor structure only if the given target is already known (Waller, 2018), which was not the case in the present research. Thus, DSL and DBF specialize in this particular data structure and degrade their performance in other data structures where assumptions of proportionality are not met (i.e., the ratio of every item's group factor loading to the general factor loading is not equal the ratio of other items belonging to the same group factor). Overall, we prioritized results obtained using iterative target rotation procedures (i.e., SLiD in particular, but also SLi).

10. Item loading matrices for other EFA and EBFA solutions examined

Information regarding the 3-factor EFA solutions and EBFA solutions with 3 specific factors are reported in the manuscript for Studies 1 and 2. We provide the full item loading matrices for the other viable factor models considered based on EFA selection criteria and EBFA tests. Specifically, EFA item loading matrices for 1, 2, and 4 factors are provided in the supplemental tables document (S1_EFAsolutions_F1toF4.xlsx & S2_EFAsolutions_F1toF4.xlsx). For alternative EBFA solutions considered (i.e., bifactor model with 2 and 4 specific factors, respectively), information regarding the top item loadings for these factor solutions are provided in the supplemental excel spreadsheet documents (S1_EBFAresults.xlsx & S2_EBFAresults.xlsx).

11. Study 2 Data Screening: Participant Attentiveness Index

An index of participant attentiveness was computed to screen for low-quality responding in participants. We specified 8 content-matched, opposite-keyed item pairs (16 items overall) to employ a squared difference approach and exclude participants based on indices of random or inattentive responding (Litman et al., 2015; Robinson et al., 2019). Four of these pairs were included as part of the larger pool of relationship items measuring specific constructs (e.g., "I am not afraid about being abandoned by my partner." – "I'm afraid my partner may abandon me."), while the other four pairs were non-relationship specific items belonging to the set of evaluative consistency bias items (e.g., "People other than my partner treat me well" – "People other than my partner treat me poorly"). A discrepancy score was calculated for every participant by squaring the discrepancy between the opposite-keyed items on the 5-point response scale (e.g., participants who 'strongly agree' that their partner feels affection for them and does not feel affection for them would receive a higher discrepancy score), then summing across the 8 item pairs. See Table S9 for items included to capture evaluative consistency bias, non-relationship

specific well-being measures, and participant attentiveness (i.e., Item Set X). This information is also included in the preregistration document.

11.1 Table S9. *Item Set X*

Item	Opposite-Keyed	Evaluative Consistency/ Non-Relationship
My partner often tells me s/he loves me.	1_a	
My partner rarely tells me s/he loves me.	1_b	
My partner does not feel affection for me.	2_a	
My partner feels affection for me.	2_b	
I am not afraid about being abandoned by my partner.	3_a	
I'm afraid my partner may abandon me.	3_b	
My needs for intimacy and companionship could NOT easily be fulfilled in an alternative relationship.	4_a	
My needs for intimacy and companionship could easily be fulfilled in an alternative relationship.	4_b	
My mental health is very good.	5_a	
My mental health is not good at all.	5_b	x
I am doing well in my professional life.	6_a	x
I am not doing well in my professional life.	6_b	x
Overall, I am satisfied with my life.	7_a	x
Overall, I am not satisfied with my life.	7_b	x
People other than my partner treat me well.	8_a	x
People other than my partner treat me poorly.	8_b	x
I have a good social life.		x
I don't feel close to my friends.		x
People other than my partner understand me.		x
I have close friendships with people other than my partner.		x
I have good athletic ability.		x
My general trivia knowledge is excellent.		x
I am a physically attractive person.		x
I consider myself to be intelligent.		x

Note. Opposite-keyed item pairs are designated by subscripts a=positive and b=negative).

12. Auxiliary CFA/CBFA tests of pre-registered factor models

As noted in the manuscript, we conducted auxiliary confirmatory analyses according to the four pre-registered hypothesized models to evaluate the evidence in support of each of the model structures (i.e., Models A-D). See supplemental file 'Aux.S2.confirmatorymodels_OSF'. More specifically, we employed confirmatory factor analyses (i.e., CFA, CBFA) in Study 2 to

test the robustness and generalizability of findings derived from exploratory results of Study 1. We note that Model A (independence model) and Model C (unidimensional model) were directly testable as measurement models since relationships between latent variables and indicators were clear. However, Model B (n-correlated-factor model) and Model D (bifactor model with n specific factors) required a priori specification of the pattern and number of correlated/specific factors, which we had intentionally left undetermined as part of our exploratory approach. To address this, we leveraged the EFA findings from Studies 1 and 2 to guide the specification of first-order factors for Model 2 (i.e., variations of correlated-factor models) and the EBFA results to inform the specific factors to be tested in Model 4 (i.e., variations of bifactor models). To compare and interpret the competing models, we did not solely rely on global model fit statistics (e.g., CFI, TLI, RMSEA, SRMR, AIC, BIC; Hu & Bentler, 2008), as previous research has cautioned against comparing hierarchical models with non-hierarchical models due to overfitting tendencies in bifactor and other hierarchical models (Markon, 2019; Bonifay & Cai, 2017; Murray & Johnson, 2013; Reise et al., 2016). Instead, we adopted recommendations to evaluate multiple fit indices alongside multiple robustness checks, including evaluating candidate models based on prior theory, assessing the strength of item loadings to determine the presence and content of factors, and applied the same bifactor metrics overviewed in the manuscript to assess factor reliability and determinacy (i.e., omegaH, omegaHS, omegaS, ECV, ECV_{SS}) (Dueber & Toland, 2021; Rodriguez et al., 2016). Due to computational limitations posed by attempting to estimate CFA and BCFA models using the entire set of 408 relationship-specific items, we adopted a strategy of item grouping based on theoretical and empirical considerations. Specifically, in a separate set of analyses, we selected the top 3 items from each construct's item set, favoring those with the strongest loadings on their respective factors (see supplemental file

'Aux.S2.confirmatorymodels_OSF'). As a result, the extensive pool of 408 items was pruned to a more manageable subset of 102 items representing the same 34 constructs.

Results soundly rejected the Independence Model (Model A), which suggests all constructs can be treated as distinct from one another. A CFA specifying the various constructs as distinct latent entities did not converge due to excessive multicollinearity across the latent constructs specified (32% of the inter-factor correlations were greater than .80). Thus, we examined an alternative multidimensional structure (Model B), in which we estimated a confirmatory model specifying three correlated factors (Positive, Negative, and Sex) based on the factor loadings identified from Study 1. Certain fit indices for this model met acceptable fit criteria (RMSEA, SRMR) while others did not (CFI, TLI): (CFI = .88, TLI = .88, RMSEA = .04, SRMR = .06). Similar results were obtained for the unidimensional model (Model C); however, considering that over 30 ostensibly distinct relationship construct were included, model fit indices were better than might be expected (CFI = .84, TLI = .84, RMSEA = .04, SRMR = .04). Closer inspection of the low-loading items in this model (<.400) showed they were mostly indicators of constructs that are more conceptually distinct from our interpretation of the Q-factor (e.g., sacrifice motives, investment, desired power). Model comparison tests favored the three-factor model (i.e., Model B); thus, we tested a bifactor model (Model D) by specifying a general factor and three (correlated) specific factors comprising a Negative factor, a Sex factor, and an Emotional Attachment factor. This model approached acceptable fit across metrics (CFI = .89, TLI = .89, RMSEA = .03, SRMR = .04). Bifactor indices indicated a strong Q factor (OmegaH = .93) represented by items reflecting positive partner regard and global satisfaction. Additionally, there was partial evidence that the three specific factors (i.e., Negative and Sex) may be interpretable as explaining substantive variance beyond Q if following certain cutoff

thresholds in the literature (e.g., Dueber & Toland, 2021) but not others (Gignac & Kretzschmar, 2017; Reise et al., 2013b). Dueber and Toland (2021) suggest that interpreting subscores corresponding to specific factors is appropriate when specific factors with moderate reliability (OmegaS = .80) have OmegaHS = .20 or ECVSS = .30. For the Negative specific factor, OmegaS = .76, OmegaHS = .52 and ECV_{SS} = .73, and. For the Sex specific factor, OmegaS = .82, OmegaHS = .38 and ECV_{SS} = .46, and. For the Emotional Attachment specific factor, OmegaS = .82, OmegaHS = .26, and ECV_{SS} = .31, and. All estimates for this are displayed in Table S10. This resulted in a model with good fit (final model: CFI = .95, TLI = .95, RMSEA = .03, SRMR = .03). Item loadings are displayed below. Model comparison tests suggested that this bifactor model fit better than the unidimensional and three correlated-factor factor. Overall, the bifactor analyses support a strong general Q factor, and provide partial evidence that the Negative and Sex (and, more modestly, Emotional Attachment) specific factors may account for reliable variance in addition to Q. Still, these findings argue against treating these domains as distinct, and suggest that any specific factor interpretation be secondary to, and conditional on, the dominance of Q.

12.1 Table S10. *Study 2 Bifactor indices for CBFA model with 3 specific factors (using reduced 102-item pool)*

**CBFA Model with 3 specific factors**		OmegaH	ECV
General factor (Q)		.929	.891
	OmegaS	OmegaHS	ECV_{SS}
Specific Factor 1 (Negative)	.756	.522	.733
Specific Factor 2 (Sex)	.817	.378	.463
Specific Factor 3 (Emotional Attachment)	.820	.261	.311

13. Clarification of Preregistered vs Implemented Evaluative Bias Analyses

In our preregistration, we described *Item Subset C* as a set of evaluative items to probe whether the general relationship factor was distinguishable from a broader general evaluative

consistency bias. Specifically, we planned to combine non-relationship-specific evaluative items with indicators of the expected general relationship factor and to use EFA/EBFA to examine their joint structure. We also referred to this broader evaluative tendency as a potential "general evaluative consistency" (GEC) influence. In the implemented analyses reported in the supplement, we retained the same core substantive goal (testing whether Q is distinguishable from a more general evaluative tendency) but report two clarifications from the preregistered plan. First, we used the EBFA on the full 408-item pool to identify pure indicators of Q (i.e., items that loaded strongly on the general factor and minimally on any specific factors). As per our preregistration, we identified the "top 8 indicators of the expected general factor", then used these eight pure Q indicators in a series of confirmatory models (CFA/CBFA) to test more directly whether Q functions as a distinct latent dimension versus merely reflecting a more global evaluative response tendency.

Second, the preregistration incorrectly states that 32 items total would be relevant for this analysis (16 relationship specific + 16 non-relationship specific), but this should in fact state 16 items total (i.e., 8 relationship specific + 8 non-relationship specific) given that the general factor Q (i.e., G_{rel} in the pre-registration) was intended to be captured by the top 8 indicators of Q. Accordingly, we restricted our specification of general evaluative consistency bias (GEC) to 8 items based on a combination of the four ECB items (i.e., the four halo bias items from Anusic et al., 2009: athletic ability, physical attractiveness, trivia knowledge, intelligence) and the four general well-being (GWB) items corresponding to the four positively-keyed general well-being items (i.e., overall life satisfaction, mental health, professional life, social life) included as part of Item Subset C.

14. Auxiliary CFA/CBFA tests to Probe Content of Q

We conducted auxiliary analyses in which we modeled a latent method factor using CFA and BCFA to further probe construct-irrelevant substantive variance attributable to evaluative consistency bias. A series of competing confirmatory models were specified and compared, with similar results suggesting Q has similar features of a more general systematic response tendency, despite being conceptually distinct.

The following confirmatory models were specified to test and compare their fit characteristics and are described as follows: 2A) a single-factor model whereby all method factor and Q indicators load onto one latent factor, 2B) a correlated two-factor model with evaluative consistency items loading on a latent method factor and a equal number of pure indicators of Q loading onto a latent Q factor with correlation between the method factor freely estimated, 2C) a constrained version of model B with the correlation between the method factor and Q set to zero, and 2D) a bifactor CFA whereby 3 types of response patterns are modelled: a general factor capturing a general evaluative consistency bias (GEC) that would generalize to ratings across relationship and non-relationship domains, alongside a specific factor Q reflecting a relationship-specific response style. Examination of item loading patterns and bifactor indices would inform the meaning of Q insofar as whether it stands on its own as a distinct specific factor and explains substantive variance or is engulfed by the influence of the general method factor (thus simply represents a subdomain of a more general evaluative consistency method artifact). Results are reported in Table S9.

First, we examined results from the EBFA model with the 408 items to identify pure indicators of Q, that is, those items which loaded most strongly on the general factor but not appreciably on any specific factor (Abad et al., 2017). We identified the top 8 items with the highest loadings on Q ($|\lambda|$range = .69 to .75), and which had loadings below .10 on any of the

other potential specific factors. These were used in conjunction with 8 evaluative consistency bias items (see Table S11 below) to allow for balanced sets of measures for each of the two latent factors to be specified.

14.1 Table S11. ***Confirmatory factor loading patterns from models testing Q as general evaluative consistency bias***

	Single-factor model (2A)	Correlated 2-factor model (2B)		Constrained correlated 2-factor model (2C)		Bifactor model (2D)	
Item	Factor 1	Factor 1	Factor 2	Factor 1	Factor 2	General Factor	Specific Factor Q
**Q (pure indicators)**							
All things considered, I am very happy in my relationship with my partner.	0.76	0.77	–	0.77	–	0.45	0.62
My partner is responsive to my needs.	0.73	0.74	–	0.74	–	0.43	0.60
I am satisfied with my partner.	0.74	0.75	–	0.75	–	0.40	0.63
My partner meets my needs.	0.73	0.74	–	0.74	–	0.44	0.59
Our relationship makes my partner very happy.	0.69	0.69	–	0.68	–	0.46	0.52
My relationship with my partner is close to ideal.	0.66	0.67	–	0.67	–	0.39	0.54
My partner is very loving and affectionate.	0.69	0.71	–	0.71	–	0.37	0.62
My partner often tells me s/he loves me.	0.68	0.70	–	0.71	–	0.33	0.64
**General Evaluative Consistency**							
Overall, I am satisfied with my life.	0.66	–	0.69	–	0.62	0.69	–
My mental health is very good.	0.41	–	0.66	–	0.67	0.66	–
I am doing well in my professional life.	0.38	–	0.62	–	0.63	0.62	–
I have a good social life.	0.44	–	0.69	–	0.70	0.69	–
I have good athletic ability.	0.31	–	0.49	–	0.51	0.49	–
I am a physically attractive person.	0.27	–	0.45	–	0.47	0.45	–
My general trivia knowledge is excellent.	0.12	–	0.30	–	0.34	0.30	–
I consider myself to be intelligent.	0.26	–	0.44	–	0.46	0.44	–
r (Factor 1, Factor 2)	NA	.57		0		NA	

In the single-factor model (Model 2A), item loadings for Q-items were all strong (≥ .68) while ECB-items ranged from low to strong (.12 - .66). This model exhibited an unsatisfactory fit (CFI = .78, SRMR = .099, RMSEA = .11). Indeed, model comparison tests showed the correlated two-factor model (Model 2B) demonstrated a superior fit (CFI = .91, SRMR = .06, RMSEA = .07, $p < .001$), and it was also superior to the constrained two-factor model (Model 2C, CFI = .87, SRMR = .17, RMSEA = .09, $p < .001$), suggesting Q indicators represent a distinguishable measurement dimension from that of the ECB indicators. The inter-factor correlation was strong (.57), suggesting Q has similar features of a more general systematic response tendency factor, despite being not completely redundant with this factor. This was further supported by examination of the bifactor model with Q specified as a specific factor (Model 2D), which indicated that OmegaH for the GEC general factor was relatively high (OmegaH = .65) suggesting some degree of a common factor underlying these items. Importantly, however, omegaHS (.61) and ECV_{SS} (.68) values for Q were high, far exceeding cutoffs for interpreting the dimensional uniqueness of a specific factors/subdomain.

15. Auxiliary Analyses with Full Sample of Excluded Participants (Studies 1 and 2)

At a reviewer's suggestion, we conducted auxiliary analyses re-analyzing the data using the entire samples for Studies 1 and 2, including respondents who failed our screening measures (e.g., attention/speeding/straightlining/inattentiveness checks). Full output is provided in the OSF project page in 'Aux.S1-S2.excludedpts_OSF'. Results were highly consistent with the main findings, with a key notable difference being that there was weaker evidence for a coherent multidimensional factor solution using EFA. In both Studies 1 and 2, EFAs no longer supported the preferred 3-factor solution: the Sex factor dropped out and solutions reduced to broader Positive/Negative factors. We attribute this to the inclusion of careless responders and low-quality participants who would introduce more noise to capture discriminant responding in the

sample (Kam, 2019). Further, the average inter-item correlations dropped and indices for the general factor attenuated (e.g., for the 3-factor EBFA, Q's ωH decreased from [.92] to [.76] in Study 1 and [.69] to [.52] in Study 2, and ECV from [.73] to [.56] in Study 1 and [.82] to [.68] in Study 2). This would be expected when low-effort cases are introduced. Importantly, this attenuation did not coincide with the emergence of coherent content factors. Overall, EFAs became notably less stable and less interpretable, and in bifactor models the subdomain indices (ωHS, ECVSS) remained below conventional thresholds for treating specific factors as substantive.

16. References for Study 1 measures listed in Table 1 and Appendix A of manuscript

Amato, P. R., & Rogers, S. J. (1997). A longitudinal study of marital problems and subsequent divorce. *Journal of Marriage and the Family*, 612-624.

Anderson, C., John, O. P., & Keltner, D. (2012). The personal sense of power. *Journal of Personality*, *80*(2), 313-344.

Arriaga, X. B., Reed, J. T., Goodfriend, W., & Agnew, C. R. (2006). Relationship perceptions and persistence: Do fluctuations in perceived partner commitment undermine dating relationships?. *Journal of Personality and Social Psychology*, *91*(6), 1045.

Barrett-Lennard, G. T. (1962). Dimensions of therapist response as causal factors in therapeutic change. *Psychological monographs: General and applied*, *76*(43), 1.

Blum, J. S., & Mehrabian, A. (1999). Personality and temperament correlates of marital satisfaction. *Journal of personality*, *67*(1), 93-125.

Booth, A., Johnson, D., & Edwards, J. N. (1983). Measuring marital instability. *Journal of Marriage and the Family*, 387-394.

Braiker, H. B., & Kelley, H. H. (1979). Conflict in the development of close relationships. *Social exchange in developing relationships*, *135*, 168.

Christensen, A., & Heavey, C. L. (1990). Gender and social structure in the demand-withdraw pattern of marital conflict. *Journal of Personality and Social Psychology, 59*, 73-81.

Christensen, A., & Sullaway, M. (1984). *Communications patterns questionnaire*. Unpublished questionnaire. Los Angeles: University of California.

Doss, B. D., & Christensen, A. (2006). Acceptance in romantic relationships: the frequency and acceptability of partner behavior inventory. *Psychological Assessment*, *18*(3), 289.

Fletcher, G. J., Simpson, J. A., Thomas, G., & Giles, L. (1999). Ideals in intimate relationships. *Journal of Personality and Social Psychology*, *76*(1), 72.

Fowers, B. J., & Olson, D. H. (1993). ENRICH Marital Satisfaction Scale: A brief research and clinical tool. *Journal of Family psychology*, *7*(2), 176.

Fraley, R. C., Heffernan, M. E., Vicary, A. M., & Brumbaugh, C. C. (2011). The experiences in close relationships—Relationship Structures Questionnaire: A method for assessing attachment orientations across relationships. *Psychological Assessment*, *23*(3), 615.

Gable, S. L., Reis, H. T., Impett, E. A., & Asher, E. R. (2004). What do you do when things go right? The intrapersonal and interpersonal benefits of sharing positive events. *Journal of Personality and Social Psychology*, *87*(2), 228.

Gordon, A. M., & Chen, S. (2016). Do you get where I'm coming from?: Perceived understanding buffers against the negative impact of conflict on relationship satisfaction. *Journal of Personality and Social Psychology*, *110*(2), 239.

Gordon, A. M., Impett, E. A., Kogan, A., Oveis, C., & Keltner, D. (2012). To have and to hold: gratitude promotes relationship maintenance in intimate bonds. *Journal of Personality and Social Psychology*, *103*(2), 257.

Hudson, W. W., Harrison, D. F., & Crosscup, P. C. (1981). A short-form scale to measure sexual discord in dyadic relationships. *Journal of Sex Research*, *17*(2), 157-174.

Huston, T. L., & Vangelisti, A. L. (1991). Socioemotional behavior and satisfaction in marital relationships: a longitudinal study. *Journal of Personality and Social Psychology*, *61*(5), 721.

Impett, E. A., Javam, L., Le, B. M., Asyabi-Eshghi, B. E., & Kogan, A. (2013). The joys of genuine giving: Approach and avoidance sacrifice motivation and authenticity. *Personal Relationships*, *20*(4), 740-754.

Kaufman, V. A., Perez, J. C., Reise, S. P., Bradbury, T. N., & Karney, B. R. (2022). Friendship network satisfaction: A multifaceted construct scored as a unidimensional scale. *Journal of Social and Personal Relationships*, *39*(2), 325-346.

Larzelere, R. E., & Huston, T. L. (1980). The dyadic trust scale: Toward understanding interpersonal trust in close relationships. *Journal of Marriage and the Family*, 595-604.

Marsh, H. W., Hau, K. T., Balla, J. R., & Grayson, D. (1998). Is more ever too much? The number of indicators per factor in confirmatory factor analysis. *Multivariate Behavioral Research*, *33*(2), 181-220.

Mattson, R. E., Rogge, R. D., Johnson, M. D., Davidson, E. K., & Fincham, F. D. (2013). The positive and negative semantic dimensions of relationship satisfaction. *Personal Relationships*, *20*(2), 328-355.

Miller, L. C., Berg, J. H., & Archer, R. L. (1983). Openers: Individuals who elicit intimate self-disclosure. *Journal of Personality and Social Psychology*, *44*(6), 1234.

Mills, J., Clark, M. S., Ford, T. E., & Johnson, M. (2004). Measurement of communal strength. *Personal Relationships*, *11*(2), 213-230.

Murphy, B. A., Casto, K. V., Watts, A. L., Costello, T. H., Jolink, T. A., Verona, E., & Algoe, S. B. (2022). “Feeling Powerful” versus “Desiring Power”: A pervasive and problematic conflation in personality assessment?. *Journal of Research in Personality*, *101*, 104305.

Norton, R. (1983). Measuring marital quality: A critical look at the dependent variable. *Journal of Marriage and the Family*, 141-151.

Park, Y., Impett, E. A., MacDonald, G., & Lemay Jr, E. P. (2019). Saying “thank you”: Partners’ expressions of gratitude protect relationship satisfaction and commitment from the harmful effects of attachment insecurity. *Journal of Personality and Social Psychology*, *117*(4), 773.

Péloquin, K., & Lafontaine, M. F. (2010). Measuring empathy in couples: Validity and reliability of the interpersonal reactivity index for couples. *Journal of Personality Assessment*, *92*(2), 146-157.

Pierce, G. R., Sarason, I. G., & Sarason, B. R. (1991). Quality of relationships inventory. *Psychological Assessment*.

Reis, H. T., Crasta, D., Rogge, R. D., Maniaci, M. R., & Carmichael, C. L. (2017). Perceived Partner Responsiveness Scale (PPRS) (Reis & Carmichael, 2006). *The sourcebook of listening research: Methodology and measures*, 516-521.

Roach, A. J., Frazier, L. P., & Bowden, S. R. (1981). The marital satisfaction scale: Development of a measure for intervention research. *Journal of Marriage and the Family*, 537-546.

Røysamb, E., Vittersø, J., & Tambs, K. (2014). The Relationship Satisfaction scale-psychometric properties. *Norsk epidemiologi*, *24*(1-2), 187-194

Rusbult, C. E., Martz, J. M., & Agnew, C. R. (1998). The investment model scale: Measuring commitment level, satisfaction level, quality of alternatives, and investment size. *Personal Relationships*, *5*(4), 357-387.

Schaefer, M. T., & Olson, D. H. (1981). Assessing intimacy: The PAIR inventory. *Journal of Marital and Family Therapy*, *7*(1), 47-60.

Schumm, W. R., Paff-Bergen, L. A., Hatch, R. C., Obiorah, F. C., Copeland, J. M., Meens, L. D., & Bugaighis, M. A. (1986). Concurrent and discriminant validity of the Kansas Marital Satisfaction Scale. *Journal of Marriage and the Family,* 381-387.

Shaw, A. M., & Rogge, R. D. (2016). Evaluating and refining the construct of sexual quality with item response theory: Development of the Quality of Sex Inventory. *Archives of Sexual Behavior*, *45*, 249-270.

Snyder, D. K. (2013). Marital satisfaction inventory. In *Handbook of measurements for marriage and family therapy* (pp. 65-71). Routledge.

Spanier, G. B. (1976). Measuring dyadic adjustment: New scales for assessing the quality of marriage and similar dyads. *Journal of Marriage and Family, 38,* 15-28.

Sprecher, S., & Regan, P. C. (1998). Passionate and companionate love in courting and young married couples. *Sociological Inquiry, 68*(2), 163-185.

Stanley, S. M., & Markman, H. J. (1992). Assessing commitment in personal relationships. *Journal of Marriage and the Family*, 595-608.

Straus, M. A., & Douglas, E. M. (2004). A short form of the Revised Conflict Tactics Scales, and typologies for severity and mutuality. *Violence and Victims, 19*(5), 507-520.

Tancredy, C. M., & Fraley, R. C. (2006). The nature of adult twin relationships: an attachment-theoretical perspective. *Journal of Personality and Social Psychology, 90*(1), 78.

Wei, M., Russell, D. W., Mallinckrodt, B., & Vogel, D. L. (2007). The Experiences in Close Relationship Scale (ECR)-short form: Reliability, validity, and factor structure. *Journal of Personality Assessment*, *88*(2), 187-204.
