# Supplementary material for: Predicting relationship quality with itself? A single general factor captures most of the variance across 34 common relationship measures
Source: PLoS One. 2026 Apr 1;21(4):e0342451. doi: 10.1371/journal.pone.0342451 (PMC13042769; doi:10.1371/journal.pone.0342451)
Supplement: S2 File — (PDF) [file pone.0342451.s002.pdf]

Appendix A

Complete list of 206 items in Study 1 and 408 items in Study 2

Construct	Item	Scale	Source	Study 1 item?
1. Affection	My partner and I kiss daily.	Comprehensive Marital Satisfaction Scale (CMSS)	Blum & Mehrabian (1999)	✓
	My partner and I laugh together.	Dyadic Adjustment Scale (DAS)	Spanier (1976)	✓
	My partner is very loving and affectionate.	Comprehensive Marital Satisfaction Scale (CMSS)	Blum & Mehrabian (1999)	✓
	I often approve of or compliment my partner.	Socioemotional Behavior Index (SBI)	Huston & Vangelisti (1991)	
	I often do something nice for my partner.	Socioemotional Behavior Index (SBI)	Huston & Vangelisti (1991)	
	I often make my partner laugh.	Socioemotional Behavior Index (SBI)	Huston & Vangelisti (1991)	
	I often say "I love you" to my partner.	Socioemotional Behavior Index (SBI)	Huston & Vangelisti (1991)	
	I often share emotions, feelings, or problems with my partner.	Socioemotional Behavior Index (SBI)	Huston & Vangelisti (1991)	
	I often share physical affection (outside sex) with my partner.	Socioemotional Behavior Index (SBI)	Huston & Vangelisti (1991)	
	I often talk about day's events with my partner.	Socioemotional Behavior Index (SBI)	Huston & Vangelisti (1991)	
	I don't get the love and affection I want from my partner.	Comprehensive Marital Satisfaction Scale (CMSS)	Blum & Mehrabian (1999)	✓
2. Affection (Perceived)	My partner often compliments me.	Adapted Frequency and Acceptability of Partner Behavior (adapted)	Doss & Christensen (2006)	✓
	My partner is friendly and warm toward me.	Barrett-Lennard Relationship Inventory (BLRT)	Barrett-Lennard (1962)	✓
	My partner does not feel affection for me.	Barrett-Lennard Relationship Inventory (BLRT) (adapted)	Barrett-Lennard (1962)	
	My partner feels affection for me.	Barrett-Lennard Relationship Inventory (BLRT)	Barrett-Lennard (1962)	✓
	My partner often tells me s/he loves me.	Adapted Frequency and Acceptability of Partner Behavior (adapted)	Doss & Christensen (2006)	✓
	My partner rarely tells me s/he loves me.	Adapted Frequency and Acceptability of Partner Behavior (adapted)	Doss & Christensen (2006)	
3. Appreciation	At times I take my partner for granted.	Appreciation in Relationships	Gordon et al. (2012)	✓
	I appreciate my partner.	Appreciation in Relationships	Gordon et al. (2012)	✓
	I make sure my partner feels appreciated.	Appreciation in Relationships	Gordon et al. (2012)	✓
	I tell my partner often that s/he is the best.	Appreciation in Relationships	Gordon et al. (2012)	✓
	I feel very lucky to have my partner in my life.	Face-valid	--	✓
	I acknowledge the things that my partner does for me, even the really small things.	Appreciation in Relationships	Gordon et al. (2012)	
	I am sometimes struck with a sense of awe and wonder when I think about my partner being in my life.	Appreciation in Relationships	Gordon et al. (2012)	
	I often tell my partner how much I appreciate her/him.	Appreciation in Relationships	Gordon et al. (2012)	
	My partner sometimes says that I fail to notice the nice things that s/he does for me.	Appreciation in Relationships	Gordon et al. (2012)	
	Sometimes I don't really acknowledge or treat my partner like s/he is someone special.	Appreciation in Relationships	Gordon et al. (2012)	
4. Appreciation (Perceived)	My partner makes me feel special.	Appreciation in Relationships	Gordon et al. (2012)	✓
	My partner makes sure I feel appreciated.	Appreciation in Relationships	Gordon et al. (2012)	✓

	My partner often tells me the things that s/he really likes about me.	Appreciation in Relationships	Gordon et al. (2012)	✓
	I feel that my partner is genuine with me.	Barrett-Lennard Relationship Inventory (BLRT)	Barrett-Lennard (1962)	✓
	I know I'm valued and appreciated by my partner.	Barrett-Lennard Relationship Inventory (BLRT)	Barrett-Lennard (1962)	✓
	My partner cares for me.	Barrett-Lennard Relationship Inventory (BLRT)	Barrett-Lennard (1962)	✓
	My partner respects me.	Barrett-Lennard Relationship Inventory (BLRT)	Barrett-Lennard (1962)	✓
	My partner expresses gratitude towards me often.	Single item	Park et al. (2019)	✓
	At times my partner takes me for granted.	Appreciation in Relationships	Gordon et al. (2012)	✓
	My partner doesn't notice when I do nice things for her/him.	Appreciation in Relationships	Gordon et al. (2012)	✓
	I feel that my partner disapproves of me.	Barrett-Lennard Relationship Inventory (BLRT)	Barrett-Lennard (1962)	✓
	My partner finds me rather dull and uninteresting.	Barrett-Lennard Relationship Inventory (BLRT)	Barrett-Lennard (1962)	✓
	My partner just tolerates or puts up with me.	Barrett-Lennard Relationship Inventory (BLRT)	Barrett-Lennard (1962)	✓
	My partner lacks respect for me.	Marital Satisfaction Scale (MSS)	Roach et al. (1981)	✓
5. Attachment anxiety (partner-specific)	I worry a lot about my relationship with my partner.	Marital Satisfaction Scale (MSS)	Roach et al. (1981)	✓
	I need a lot of reassurance that I am loved by my partner.	Experiences in Close Relationships - Short Form	Wei et al. (2007)	
	I often worry about whether my partner really cares for me.	Experiences in Close Relationships - Relationship Structures Questionnaire	Fraley et al. (2011)	
	I worry that my partner won't care about me as much as I care about them.	Experiences in Close Relationships - Relationship Structures Questionnaire	Fraley et al. (2011)	
	I'm afraid my partner may abandon me.	Experiences in Close Relationships - Relationship Structures Questionnaire	Fraley et al. (2011)	
	I am not afraid about being abandoned by my partner.	Experiences in Close Relationships - Short Form (adapted)	Wei et al. (2007)	
6. Attachment avoidance (partner-specific)	I don't feel comfortable opening up to my partner.	Experiences in Close Relationships - Relationship Structures Questionnaire	Fraley et al. (2011)	
	I find it easy to depend on my partner.	Experiences in Close Relationships - Relationship Structures Questionnaire	Fraley et al. (2011)	
	I prefer not to show my partner how I feel deep down.	Experiences in Close Relationships - Relationship Structures Questionnaire	Fraley et al. (2011)	
	I usually talk things over with my partner.	Experiences in Close Relationships - Relationship Structures Questionnaire	Fraley et al. (2011)	
	I usually discuss my problems and concerns with my partner.	Experiences in Close Relationships - Relationship Structures Questionnaire	Fraley et al. (2011)	
	It helps to turn to my partner in times of need.	Experiences in Close Relationships - Relationship Structures Questionnaire	Fraley et al. (2011)	
7. Capitalization	When I tell my partner about something good that has happened to me, I sometimes get the sense that my partner is even more happy and excited than I am.	Perceived Responses to Capitalization Attempts Scale	Gable et al. (2004)	
	When I tell my partner about something good that has happened to me, my partner often asks a lot of questions and shows genuine concern about the good event.	Perceived Responses to Capitalization Attempts Scale	Gable et al. (2004)	
	When I tell my partner about something good that has happened to me, my partner usually reacts to my good fortune enthusiastically.	Perceived Responses to Capitalization Attempts Scale	Gable et al. (2004)	

	When I tell my partner about something good that has happened to me, he/she points out the potential down sides of the good event.	Perceived Responses to Capitalization Attempts Scale	Gable et al. (2004)	
	When I tell my partner about something good that has happened to me, my partner often finds problems with it.	Perceived Responses to Capitalization Attempts Scale	Gable et al. (2004)	
	When I tell my partner about something good that has happened to me, my partner reminds me that most good things have their bad aspects as well.	Perceived Responses to Capitalization Attempts Scale	Gable et al. (2004)	
	When I tell my partner about something good that has happened to me, my partner is usually silently supportive of the good things that occur to me.	Perceived Responses to Capitalization Attempts Scale	Gable et al. (2004)	
	When I tell my partner about something good that has happened to me, my partner says little, but I know he/she is happy for me.	Perceived Responses to Capitalization Attempts Scale	Gable et al. (2004)	
	When I tell my partner about something good that has happened to me, my partner tries not to make a big deal out of it, but is happy for me.	Perceived Responses to Capitalization Attempts Scale	Gable et al. (2004)	
	When I tell my partner about something good that has happened to me, my partner doesn't pay much attention to me.	Perceived Responses to Capitalization Attempts Scale	Gable et al. (2004)	
	When I tell my partner about something good that has happened to me, my partner often seems disinterested.	Perceived Responses to Capitalization Attempts Scale	Gable et al. (2004)	
	When I tell my partner about something good that has happened to me, I sometimes get the impression that he/she doesn't care that much.	Perceived Responses to Capitalization Attempts Scale	Gable et al. (2004)	
8. Commitment	I want this relationship to stay strong no matter what rough times we may encounter.	Commitment Inventory	Stanley & Markman (1992)	✓
	I want to grow old with my partner.	Commitment Inventory	Stanley & Markman (1992)	✓
	I am committed to maintaining my relationship with my partner.	Investment Model Scale	Rusbult et al. (1998)	✓
	I want our relationship to last a very long time.	Investment Model Scale	Rusbult et al. (1998)	✓
	I would not feel very upset if my relationship with my partner were to end in the near future.	Investment Model Scale	Rusbult et al. (1998)	✓
	The future of my relationship with my partner looks promising to me.	Marital Satisfaction Scale (MSS)	Roach et al. (1981)	✓
	It is hard to imagine my life without my partner.	Friendship Network Satisfaction Scale (adapted)	Kaufman et al. (2021)	✓
	My relationship with my partner is very stable.	Quality of Marriage Index (QMI)	Norton (1983)	✓
	I am oriented toward the long-term future of my relationship (for example, I imagine being with my partner several years from now).	Investment Model Scale	Rusbult et al. (1998)	
	I feel very attached to our relationship--very strongly linked to my partner.	Investment Model Scale	Rusbult et al. (1998)	
	I want my relationship with my partner to last forever.	Investment Model Scale	Rusbult et al. (1998)	
	It is likely that I will date someone other than my partner within the next year.	Investment Model Scale	Rusbult et al. (1998)	
	I have discussed ending the relationship with friends and family members.	Marital Instability Scale	Booth et al. (1983)	✓
	I would enjoy living apart from my partner.	Marital Instability Scale	Booth et al. (1983)	✓
	My partner and I often discuss or consider separation or ending our relationship.	Dyadic Adjustment Scale (DAS)	Spanier (1976)	✓

	The future of my relationship with my partner is too uncertain to make serious plans.	Marital Satisfaction Inventory (MSI)	Snyder (1979)	✓
	I often consider ending my relationship with my partner.	Relationship Satisfaction scale (RS)	Roysamb et al. (2014)	✓
9. Commitment (Perceived)	I think my partner feels trapped in our relationship.	Face-valid	--	✓
	I think my partner is dedicated to our relationship.	Face-valid	--	✓
	My partner wants our relationship to last forever.	Face-valid	--	✓
	I think my partner is committed to maintaining our relationship.	Face-valid	--	✓
	My partner feels very attached to our relationship -- strongly linked to me.	Perceived Partner Commitment	Arriaga et al. (2006)	
	My partner intends to stay in this relationship.	Perceived Partner Commitment	Arriaga et al. (2006)	
	My partner is committed to maintaining our relationship.	Perceived Partner Commitment	Arriaga et al. (2006)	
	My partner is oriented toward the long-term future of our relationship (for example, imagines being with me several years from now).	Perceived Partner Commitment	Arriaga et al. (2006)	
	My partner is too flirtatious with other men/women.	Frequency and Acceptability of Partner Behavior (adapted)	Doss & Christensen (2006)	✓
	My partner has seriously suggested the idea of ending the relationship.	Marital Instability Scale (adapted)	Booth et al. (1983)	✓
	My partner thinks our relationship is in trouble.	Marital Instability Scale	Booth et al. (1983)	✓
	I think my partner is unfaithful.	Face-valid	--	✓
10. Communal Strength	My partner meets my needs.	Relationship Assessment Scale (RAS)	Hendrick (1988)	✓
	I feel happy when I do something that helps my partner.	Communal Strength	Mills et al. (2004)	✓
	I would be willing to give up a lot to benefit my partner.	Communal Strength	Mills et al. (2004)	✓
	I would go out of my way to do something for my partner.	Communal Strength	Mills et al. (2004)	✓
	Meeting the needs of my partner is a high priority for me.	Communal Strength	Mills et al. (2004)	✓
	I would be willing to go far to visit my partner.	Communal Strength	Mills et al. (2004)	
	I would be likely to give my partner large benefits.	Communal Strength	Mills et al. (2004)	
	I would incur large costs to meet the needs of my partner.	Communal Strength	Mills et al. (2004)	
	It would be easy for me to accept not helping my partner.	Communal Strength	Mills et al. (2004)	
	I can readily put the needs of my partner out of my thoughts.	Communal Strength	Mills et al. (2004)	
	I would be reluctant to sacrifice for my partner.	Communal Strength	Mills et al. (2004)	
11. Communication	My partner and I talk about the quality of our relationship often.	Braiker-Kelley Partnership Questionnaire	Braiker & Kelley (1979)	✓
	I tell my partner what I want or need from the relationship.	Braiker-Kelley Partnership Questionnaire	Braiker & Kelley (1979)	✓
	My partner and I settle our disagreements with mutual give and take.	Comprehensive Marital Satisfaction Scale (CMSS)	Blum & Mehrabian (1999)	✓
	I am very happy about how we make decisions and resolve conflicts.	ENRICH Marital Satisfaction Scale (EMS)	Fowers & Olson (1993)	✓
	When I have a problem, I can talk to my partner about it.	Friendship Network Satisfaction Scale (adapted)	Kaufman et al. (2021)	✓
	My partner and I do not communicate well with each other.	Comprehensive Marital Satisfaction Scale (CMSS)	Blum & Mehrabian (1999)	✓
12. Conflict Frequency	My partner and I argue with each other often.	Braiker-Kelley Partnership Questionnaire	Braiker & Kelley (1979)	✓
	My partner and I often argue about finances.	Comprehensive Marital Satisfaction Scale (CMSS)	Blum & Mehrabian (1999)	✓
	My partner and I often get on each other's nerves.	Dyadic Adjustment Scale (DAS)	Spanier (1976)	✓

	Minor disagreements with my partner often end up in big arguments.	Marital Satisfaction Inventory (MSI)	Snyder (1979)	✓
	My partner and I seem able to go for days sometimes without settling our differences.	Marital Satisfaction Inventory (MSI)	Snyder (1979)	✓
	My partner and I have problems in our relationship.	Relationship Satisfaction scale (RS)	Roysamb et al. (2014)	✓
	I feel like all my partner and I do is fight.	Conflict Frequency	Gordon & Chen (2016)	
	I am often irritated by my partner.	Conflict Frequency	Gordon & Chen (2016)	
	It is rare that my partner and I get in a big argument.	Conflict Frequency	Gordon & Chen (2016)	
	My partner and I are always in agreement on major issues.	Conflict Frequency	Gordon & Chen (2016)	
	My partner and I have a lot of disagreements.	Conflict Frequency	Gordon & Chen (2016)	
	There is a lot of conflict in my relationship with my partner.	Conflict Frequency	Gordon & Chen (2016)	
13. Conflict Strategies	During a discussion of a relationship issue or problem, my partner and I blame, accuse, and criticize one another.	Communication Patterns Questionnaire - short form (CPQ-SF)	Christensen & Heavey (1990)	✓
	During a discussion of a relationship issue or problem, my partner and I express our feelings to each other.	Communication Patterns Questionnaire - short form (CPQ-SF)	Christensen & Heavey (1990)	✓
	When we have problems, my partner and I suggest possible solutions and compromises.	Communication Patterns Questionnaire - short form (CPQ-SF)	Christensen & Heavey (1990)	✓
	During a discussion of a relationship issue or problem, my partner and I feel understood by each other.	Communication Patterns Questionnaire (CPQ)	Christensen & Heavey (1990)	✓
	When we have problems, my partner and I avoid discussing the problem.	Communication Patterns Questionnaire - short form (CPQ-SF)	Christensen & Heavey (1990)	✓
	When we have problems, my partner and I try to discuss the problem.	Communication Patterns Questionnaire - short form (CPQ-SF)	Christensen & Heavey (1990)	✓
	When we have problems, I call my partner names, swear at them, or attack their character.	Communication Patterns Questionnaire (CPQ)	Christensen & Sullaway (1984)	✓
	When we have problems, I push, shove, slap, hit, or kick my partner.	Communication Patterns Questionnaire (CPQ)	Christensen & Sullaway (1984)	✓
	When we have problems, my partner and I threaten one another with negative consequences.	Communication Patterns Questionnaire (CPQ)	Christensen & Sullaway (1984)	✓
	When we have problems, my partner and I try to be especially nice to each other.	Communication Patterns Questionnaire (CPQ)	Christensen & Sullaway (1984)	✓
	When we have problems, my partner calls me names, swears at me, or attacks my character.	Communication Patterns Questionnaire (CPQ)	Christensen & Sullaway (1984)	✓
	When we have problems, my partner pushes, shoves, slaps, hits, or kicks me.	Communication Patterns Questionnaire (CPQ)	Christensen & Sullaway (1984)	✓
	My partner gives me sufficient opportunity to express my opinions.	Marital Satisfaction Scale (MSS)	Roach et al. (1981)	✓
	My partner is willing to make helpful improvements in our relationship.	Marital Satisfaction Scale (MSS)	Roach et al. (1981)	✓
	There are times when my partner is dishonest with me.	Frequency and Acceptability of Partner Behavior (adapted)	Doss & Christensen (2006)	✓
	My partner's habits annoy me.	Comprehensive Marital Satisfaction Scale (CMSS)	Blum & Mehrabian (1999)	✓
	My partner gets me badly flustered and jittery.	Marital Satisfaction Scale (MSS)	Roach et al. (1981)	✓
	During a discussion of a relationship issue or problem, I criticize while my partner defends themself.	Communication Patterns Questionnaire - short form (CPQ-SF)	Christensen & Heavey (1990)	
	During a discussion of a relationship issue or problem, I pressure, nag, or demand while my partner withdraws, becomes silent, or refuses to discuss further.	Communication Patterns Questionnaire - short form (CPQ-SF)	Christensen & Heavey (1990)	

	During a discussion of a relationship issue or problem, I try to start a discussion while my partner tries to avoid a discussion.	Communication Patterns Questionnaire - short form (CPQ-SF)	Christensen & Heavey (1990)	
	During a discussion of a relationship issue or problem, my partner criticizes while I defend myself.	Communication Patterns Questionnaire - short form (CPQ-SF)	Christensen & Heavey (1990)	
	During a discussion of a relationship issue or problem, my partner pressures, nags, or demands while I withdraw, become silent, or refuse to discuss further.	Communication Patterns Questionnaire - short form (CPQ-SF)	Christensen & Heavey (1990)	
	During a discussion of a relationship issue or problem, my partner tries to start a discussion while I try to avoid a discussion.	Communication Patterns Questionnaire - short form (CPQ-SF)	Christensen & Heavey (1990)	
14. Empathy (empathic concern)	I often have tender, concerned feelings for my partner when he/she is less fortunate than me.	Interpersonal Reactivity Index for Couples (shortened-10)	Peloquin & Lafontaine (2010)	
	In my relationship with my partner, I would describe myself as a pretty soft-hearted person.	Interpersonal Reactivity Index for Couples (shortened-10)	Peloquin & Lafontaine (2010)	
	My partner's misfortunes do not usually disturb me a great deal.	Interpersonal Reactivity Index for Couples (shortened-10)	Peloquin & Lafontaine (2010)	
	Sometimes I don't feel very sorry for my partner when he/she is having problems.	Interpersonal Reactivity Index for Couples (shortened-10)	Peloquin & Lafontaine (2010)	
	When I see my partner being treated unfairly, I sometimes don't feel very much pity for him/her.	Interpersonal Reactivity Index for Couples (shortened-10)	Peloquin & Lafontaine (2010)	
	Before criticizing my partner, I try to imagine how I would feel if I were in his/her place.	Interpersonal Reactivity Index for Couples (shortened-10)	Peloquin & Lafontaine (2010)	
	I sometimes try to understand my partner better by imagining how things look from his/her perspective.	Interpersonal Reactivity Index for Couples (shortened-10)	Peloquin & Lafontaine (2010)	
	I try to look at my partner's side of a disagreement before I make a decision.	Interpersonal Reactivity Index for Couples (shortened-10)	Peloquin & Lafontaine (2010)	
	In my relationship, I believe that there are two sides to every question and try to look at them both.	Interpersonal Reactivity Index for Couples (shortened-10)	Peloquin & Lafontaine (2010)	
	When I'm upset at my partner, I usually try to "put myself in his/her shoes" for a while.	Interpersonal Reactivity Index for Couples (shortened-10)	Peloquin & Lafontaine (2010)	
15. Goal Compatibility	My partner and I agree on how to spend our leisure time.	Comprehensive Marital Satisfaction Scale (CMSS)	Blum & Mehrabian (1999)	✓
	My partner and I agree on how we handle our finances.	Comprehensive Marital Satisfaction Scale (CMSS)	Blum & Mehrabian (1999)	✓
	My partner and I agree on our dealings with our in-laws.	Comprehensive Marital Satisfaction Scale (CMSS)	Blum & Mehrabian (1999)	✓
	My partner and I have similar ambitions and goals.	Comprehensive Marital Satisfaction Scale (CMSS)	Blum & Mehrabian (1999)	✓
	My partner and I often agree about major decisions.	Comprehensive Marital Satisfaction Scale (CMSS)	Blum & Mehrabian (1999)	✓
	My partner and I share the same basic philosophy of life.	Comprehensive Marital Satisfaction Scale (CMSS)	Blum & Mehrabian (1999)	✓
	My partner and I agree on career decisions.	Dyadic Adjustment Scale (DAS)	Spanier (1976)	✓
	My partner and I agree on household tasks.	Dyadic Adjustment Scale (DAS)	Spanier (1976)	✓
	My partner and I engage in outside interests together.	Dyadic Adjustment Scale (DAS)	Spanier (1976)	✓
	My partner and I agree on how children should be raised.	Relationship Satisfaction scale (RS)	Roysamb et al. (2014)	✓
	My partner and I work together on projects.	Dyadic Adjustment Scale (DAS)	Spanier (1976)	✓
	I feel very good about how my partner and I practice our religious beliefs and values.	ENRICH Marital Satisfaction Scale (EMS)	Fowers & Olson (1993)	✓
	My partner supports my career goals.	Face-valid	--	✓
	I prefer doing things without my partner.	Comprehensive Marital Satisfaction Scale (CMSS)	Blum & Mehrabian (1999)	✓
	My partner and I differ on our general values and beliefs.	Comprehensive Marital Satisfaction Scale (CMSS)	Blum & Mehrabian (1999)	✓
	My partner and I disagree on sexual matters.	Comprehensive Marital Satisfaction Scale (CMSS)	Blum & Mehrabian (1999)	✓

16. Inclusion of Other in Self	Which of these circles best describes your relationship with your partner?	Inclusion of Other in Self Scale	Aron et al. (1992)	
17. Intimacy	My partner inspires me to do my best work.	Marital Satisfaction Scale (MSS)	Roach et al. (1981)	✓
	I really feel like part of a team with my partner.	Quality of Marriage Index (QMI)	Norton	✓
	I attend social events with my partner.	Friendship Network Satisfaction Scale (adapted)	Kaufman et al. (2021)	✓
	My partner and I don't have much in common to talk about.	Marital Satisfaction Inventory (MSI)	Snyder (1979)	✓
	I have meaningful conversations with my partner.	Friendship Network Satisfaction Scale (adapted)	Kaufman et al. (2021)	✓
	I like to hang out with my partner.	Friendship Network Satisfaction Scale (adapted)	Kaufman et al. (2021)	✓
	I spend free time with my partner.	Friendship Network Satisfaction Scale (adapted)	Kaufman et al. (2021)	✓
	My partner and I eat together often.	Friendship Network Satisfaction Scale (adapted)	Kaufman et al. (2021)	✓
	My partner celebrates my good news.	Friendship Network Satisfaction Scale (adapted)	Kaufman et al. (2021)	✓
	My partner and I have fun together.	Friendship Network Satisfaction Scale (adapted)	Kaufman et al. (2021)	✓
	My partner and I like to hang out with our friends together.	Face-valid	--	✓
	My partner and I try new things together.	Face-valid	--	✓
	I have a close relationship with my partner.	Relationship Satisfaction scale (RS)	Roysamb et al. (2014)	✓
	My partner makes unfair demands of my free time.	Marital Satisfaction Scale (MSS)	Roach et al. (1981)	✓
	My relationship with my partner is close.	PRQC	Fletcher et al. (2000)	
	I am connected to my partner.	PRQC	Fletcher et al. (2000)	
	My relationship with my partner is intimate.	PRQC	Fletcher et al. (2000)	
18. Intimacy (Domain-specific)	I feel neglected at times by my partner.	Personal Assessment of Intimacy in Relationships (PAIR)	Schaefer & Olson (1981)	✓
	I often feel distant from my partner.	Personal Assessment of Intimacy in Relationships (PAIR)	Schaefer & Olson (1981)	✓
	My partner listens to me when I need someone to talk to.	Personal Assessment of Intimacy in Relationships (PAIR)	Schaefer & Olson (1981)	✓
	My partner has all the qualities I've ever wanted in a mate.	Personal Assessment of Intimacy in Relationships (PAIR)	Schaefer & Olson (1981)	✓
	I feel it is useless to discuss some things with my partner.	Personal Assessment of Intimacy in Relationships (PAIR)	Schaefer & Olson (1981)	✓
	My partner frequently tries to change my ideas.	Personal Assessment of Intimacy in Relationships (PAIR)	Schaefer & Olson (1981)	✓
	My partner helps me clarify my thoughts.	Personal Assessment of Intimacy in Relationships (PAIR)	Schaefer & Olson (1981)	✓
	I share in many of my partner's interests.	Personal Assessment of Intimacy in Relationships (PAIR)	Schaefer & Olson (1981)	✓
	My partner and I enjoy the same recreational activities.	Personal Assessment of Intimacy in Relationships (PAIR)	Schaefer & Olson (1981)	✓
	My partner and I like playing together.	Personal Assessment of Intimacy in Relationships (PAIR)	Schaefer & Olson (1981)	✓
	My partner and I make time to do fun things together.	Personal Assessment of Intimacy in Relationships (PAIR)	Schaefer & Olson (1981)	✓
	I am able to tell my partner when I want sexual intercourse.	Personal Assessment of Intimacy in Relationships (PAIR)	Schaefer & Olson (1981)	✓

	I feel our sexual activity is just routine.	Personal Assessment of Intimacy in Relationships (PAIR)	Schaefer & Olson (1981)	✓
	My partner seems disinterested in sex.	Personal Assessment of Intimacy in Relationships (PAIR)	Schaefer & Olson (1981)	✓
	Many of my partner's closest friends are also my closest friends.	Personal Assessment of Intimacy in Relationships (PAIR)	Schaefer & Olson (1981)	✓
	My partner disapproves of some of my friends.	Personal Assessment of Intimacy in Relationships (PAIR)	Schaefer & Olson (1981)	✓
	My partner and I enjoy spending time with other couples.	Personal Assessment of Intimacy in Relationships (PAIR)	Schaefer & Olson (1981)	✓
	My partner and I have very few friends in common.	Personal Assessment of Intimacy in Relationships (PAIR)	Schaefer & Olson (1981)	✓
19. IPV	In our relationship, I often destroy something belonging to my partner or threaten to hit my partner.	Conflict Tactics Scale Revised - Short	Straus & Douglas (2004)	
	In our relationship, I often explain my side or suggest a compromise for a disagreement with my partner.	Conflict Tactics Scale Revised - Short	Straus & Douglas (2004)	
	In our relationship, I often have a sprain, bruise, or small cut because of a fight with my partner.	Conflict Tactics Scale Revised - Short	Straus & Douglas (2004)	
	In our relationship, I often insult or swear at my partner.	Conflict Tactics Scale Revised - Short	Straus & Douglas (2004)	
	In our relationship, I often punch, kick, or beat-up my partner.	Conflict Tactics Scale Revised - Short	Straus & Douglas (2004)	
	In our relationship, I often push, shove, or slap my partner.	Conflict Tactics Scale Revised - Short	Straus & Douglas (2004)	
	In our relationship, I often show respect for my partner's feelings about an issue we disagreed on.	Conflict Tactics Scale Revised - Short	Straus & Douglas (2004)	
	In our relationship, I often use force (like hitting, holding down, or using a weapon) to make my partner have sex.	Conflict Tactics Scale Revised - Short	Straus & Douglas (2004)	
	In our relationship, my partner often destroys something belonging to me or threatens to hit me.	Conflict Tactics Scale Revised - Short	Straus & Douglas (2004)	
	In our relationship, my partner often explains their side or suggests a compromise.	Conflict Tactics Scale Revised - Short	Straus & Douglas (2004)	
	In our relationship, my partner often has a sprain, bruise, or small cut because of a fight with me.	Conflict Tactics Scale Revised - Short	Straus & Douglas (2004)	
	In our relationship, my partner often insults me or swears at me.	Conflict Tactics Scale Revised - Short	Straus & Douglas (2004)	
	In our relationship, my partner often punches, kicks, or beats me up.	Conflict Tactics Scale Revised - Short	Straus & Douglas (2004)	
	In our relationship, my partner often pushes, shoves, or slaps me.	Conflict Tactics Scale Revised - Short	Straus & Douglas (2004)	
	In our relationship, my partner often shows respect for my feelings about an issue we disagree on.	Conflict Tactics Scale Revised - Short	Straus & Douglas (2004)	
	In our relationship, my partner often uses force (like hits, holds me down, or uses a weapon) to make me have sex.	Conflict Tactics Scale Revised - Short	Straus & Douglas (2004)	
20. Investment	I have told my partner many private things about myself.	Investment Model Scale (adapted)	Rusbult et al. (1998)	✓
				✓
	My partner and I share many memories.	Investment Model Scale (adapted)	Rusbult et al. (1998)	✓
	I have invested a great deal into our relationship that I would lose if the relationship were to end.	Investment Model Scale	Rusbult et al. (1998)	✓
	Compared to other people I know, I have invested a great deal in my relationship with my partner.	Investment Model Scale	Rusbult et al. (1998)	✓

	Many aspects of my life have become linked to my partner (recreational activities, etc.) and I would lose all of this if we were to break up.	Investment Model Scale	Rusbult et al. (1998)	✓
	I feel very involved in my relationship with my partner - like I have put a great deal into it.	Investment Model Scale	Rusbult et al. (1998)	
	My relationships with friends and family members would be complicated if my partner and I were to break up (e.g. my partner is friends with people I care about).	Investment Model Scale	Rusbult et al. (1998)	
	I feel trapped in my relationship with my partner.	Braiker-Kelley Partnership Questionnaire	Braiker & Kelley (1979)	✓
21. Love	I adore my partner.	PRQC	Fletcher et al. (2000)	✓
	I love my partner.	PRQC	Fletcher et al. (2000)	✓
	I care about my partner.	Companionate Love Scale	Sprecher & Regan (1998)	
	I feel that I can confide in my partner about virtually everything.	Companionate Love Scale	Sprecher & Regan (1998)	
	I feel that I can trust my partner completely.	Companionate Love Scale	Sprecher & Regan (1998)	
	I find it easy to ignore my partner's faults.	Companionate Love Scale	Sprecher & Regan (1998)	
	I would do almost anything for my partner.	Companionate Love Scale	Sprecher & Regan (1998)	
	I would forgive my partner for practically anything.	Companionate Love Scale	Sprecher & Regan (1998)	
	I would greatly enjoy being confided in by my partner.	Companionate Love Scale	Sprecher & Regan (1998)	
	I cherish my partner.	PRQC	Fletcher et al. (2000)	
22. Normative Attachment	I feel very attached to my partner.	Braiker-Kelley Partnership Questionnaire	Braiker & Kelley (1979)	✓
	I make an effort to stay in contact with my partner.	Attachment Features and Functions	Tancredy & Fraley (2006)	
	It is important to me to see or talk with my partner regularly.	Attachment Features and Functions	Tancredy & Fraley (2006)	
	My partner is the first person that I would turn to if I had a problem.	Attachment Features and Functions	Tancredy & Fraley (2006)	
	My partner is the person that I would want to go to, to help me feel better when something bad happens to me or I feel upset.	Attachment Features and Functions	Tancredy & Fraley (2006)	
	If I achieved something good, my partner is the person that I would tell first.	Attachment Features and Functions	Tancredy & Fraley (2006)	
	My partner is the person that I would like to be able to count on to always be there for me and care about me no matter what.	Attachment Features and Functions	Tancredy & Fraley (2006)	
	My life would be severely disrupted if my partner was no longer a part of it.	Attachment Features and Functions	Tancredy & Fraley (2006)	
	When I am away from my partner, I feel down.	Attachment Features and Functions	Tancredy & Fraley (2006)	
23. Partner Traits	My partner is one of the best people I know.	Marital Satisfaction Scale (MSS)	Roach et al. (1981)	✓
	My partner has a good job.	Positive Ideals Scale	Fletcher et al. (1999)	
	My partner is adventurous.	Positive Ideals Scale	Fletcher et al. (1999)	
	My partner dresses well.	Positive Ideals Scale	Fletcher et al. (1999)	
	My partner has a nice body.	Positive Ideals Scale	Fletcher et al. (1999)	
	My partner is attractive.	Positive Ideals Scale	Fletcher et al. (1999)	
	My partner is considerate.	Positive Ideals Scale	Fletcher et al. (1999)	
	My partner is financially secure.	Positive Ideals Scale	Fletcher et al. (1999)	
	My partner is kind.	Positive Ideals Scale	Fletcher et al. (1999)	
	My partner is outgoing.	Positive Ideals Scale	Fletcher et al. (1999)	

	My partner is sexy.	Positive Ideals Scale	Fletcher et al. (1999)	
	My partner is successful.	Positive Ideals Scale	Fletcher et al. (1999)	
	My partner is understanding.	Positive Ideals Scale	Fletcher et al. (1999)	
	I don't approve of the way my partner relates to my family.	Comprehensive Marital Satisfaction Scale (CMSS)	Blum & Mehrabian (1999)	✓
	My partner drinks or uses drugs.	Marital Problems Scale	Amato & Rogers (1997)	✓
	My partner gets angry easily.	Marital Problems Scale	Amato & Rogers (1997)	✓
	My partner has feelings that are easily hurt.	Marital Problems Scale	Amato & Rogers (1997)	✓
	My partner is critical.	Marital Problems Scale	Amato & Rogers (1997)	✓
	My partner is domineering.	Marital Problems Scale	Amato & Rogers (1997)	✓
	My partner is jealous.	Marital Problems Scale	Amato & Rogers (1997)	✓
	My partner is moody.	Marital Problems Scale	Amato & Rogers (1997)	✓
24. Passion	My relationship is passionate.	PRQC	Fletcher et al. (2000)	✓
	My relationship is lustful.	PRQC	Fletcher et al. (2000)	
	My relationship is sexually intense.	PRQC	Fletcher et al. (2000)	
	I eagerly look for signs indicating my partner's desire for me.	Passionate Love Scale	Sprecher & Regan (1998)	
	I get extremely depressed when things don't go right in my relationship with my partner.	Passionate Love Scale	Sprecher & Regan (1998)	
	I have an endless appetite for affection from my partner.	Passionate Love Scale	Sprecher & Regan (1998)	
	I possess a powerful attraction for my partner.	Passionate Love Scale	Sprecher & Regan (1998)	
	I want my partner physically, emotionally, mentally.	Passionate Love Scale	Sprecher & Regan (1998)	
	I would feel deep despair if my partner left me.	Passionate Love Scale	Sprecher & Regan (1998)	
	I would rather be with my partner than anyone else.	Passionate Love Scale	Sprecher & Regan (1998)	
	I'd get jealous if l thought my partner were falling in love with someone else.	Passionate Love Scale	Sprecher & Regan (1998)	
	My partner always seems to be on my mind.	Passionate Love Scale	Sprecher & Regan (1998)	
	Sometimes I feel I can't control my thoughts; they are obsessively on my partner.	Passionate Love Scale	Sprecher & Regan (1998)	
25. Perceived Partner Responsiveness	My partner usually seems interested in doing things with me.	Perceived Partner Responsiveness Scale	Reis et al. (2017)	✓
	My partner is generally understanding.	Relationship Satisfaction scale (RS)	Roysamb et al. (2014)	✓
	My partner understands me.	Friendship Network Satisfaction Scale (adapted)	Kaufman et al. (2021)	✓
	My partner knows me well.	Perceived Partner Responsiveness Scale	Reis et al. (2017)	✓
	My partner is responsive to my needs.	Perceived Partner Responsiveness Scale	Reis et al. (2017)	✓
	My partner seems interested in what I am thinking and feeling.	Perceived Partner Responsiveness Scale	Reis et al. (2017)	✓
	My partner "gets the facts right” about me.	Perceived Partner Responsiveness Scale	Reis et al. (2017)	
	My partner esteems me, shortcomings and all.	Perceived Partner Responsiveness Scale	Reis et al. (2017)	
	My partner expresses liking and encouragement for me.	Perceived Partner Responsiveness Scale	Reis et al. (2017)	
	My partner is "on the same wavelength” with me.	Perceived Partner Responsiveness Scale	Reis et al. (2017)	
	My partner really listens to me.	Perceived Partner Responsiveness Scale	Reis et al. (2017)	
	My partner sees the "real” me.	Perceived Partner Responsiveness Scale	Reis et al. (2017)	
	My partner values and respects the whole package that is the "real” me.	Perceived Partner Responsiveness Scale	Reis et al. (2017)	
	My partner values my abilities and opinions.	Perceived Partner Responsiveness Scale	Reis et al. (2017)	
26. Power (Felt/Desired)	My partner regards me as an equal.	Marital Satisfaction Scale (MSS)	Roach et al. (1981)	✓

	I can get my partner to listen to what I say.	Sense of Power Scale	Anderson et al. (2012)	
	If I want to, I get to make decisions in my romantic relationship.	Sense of Power Scale	Anderson et al. (2012)	
	I think I have a great deal of power in my romantic relationship.	Sense of Power Scale	Anderson et al. (2012)	
	I can get my partner to do what I want.	Sense of Power Scale	Anderson et al. (2012)	
	My views have little sway, even if I voice them to my partner.	Sense of Power Scale	Anderson et al. (2012)	
	My ideas and wishes are often ignored by partner.	Sense of Power Scale	Anderson et al. (2012)	
	My wishes do not carry much weight in my romantic relationship.	Sense of Power Scale	Anderson et al. (2012)	
	I am not able to get my way with my partner, even when I try.	Sense of Power Scale	Anderson et al. (2012)	
	I have a strong drive to get power in my romantic relationship.	Feeling Powerful and Desiring Power Scales	Murphy et al. (2022)	
	I like to have power over my partner.	Feeling Powerful and Desiring Power Scales	Murphy et al. (2022)	
	I would enjoy having authority over my partner.	Feeling Powerful and Desiring Power Scales	Murphy et al. (2022)	
	I work to control my partner more than they control me.	Feeling Powerful and Desiring Power Scales	Murphy et al. (2022)	
	I try to have more influence than my partner.	Feeling Powerful and Desiring Power Scales	Murphy et al. (2022)	
	I like to tell my partner what they should do.	Feeling Powerful and Desiring Power Scales	Murphy et al. (2022)	
27. Quality of alternatives	My life would seem empty without my relationship to my partner.	Marital Satisfaction Scale (MSS)	Roach et al. (1981)	✓
	My alternatives to our relationship are close to ideal (dating another, spending time with friends or on my own, etc.).	Investment Model Scale	Rusbult et al. (1998)	
	If I weren't with my dating partner, I would do fine- I'd find another appealing person to date.	Investment Model Scale	Rusbult et al. (1998)	
	My alternatives are attractive to me (dating another, spending time with friends or on my own, etc.).	Investment Model Scale	Rusbult et al. (1998)	
	The people other than my partner with whom I might become involved with are very appealing.	Investment Model Scale	Rusbult et al. (1998)	
	My needs for intimacy and companionship could NOT easily be fulfilled in an alternative relationship.	Investment Model Scale	Rusbult et al. (1998)	
	My needs for intimacy and companionship could easily be fulfilled in an alternative relationship.	Investment Model Scale	Rusbult et al. (1998)	
28. Sacrifice Motives	When sacrificing for my partner, I generally do so to make my partner feel loved.	Impett Approach Avoidance Motives Scale	Impett et al. (2013)	
	When sacrificing for my partner, I generally do so to make my partner happy.	Impett Approach Avoidance Motives Scale	Impett et al. (2013)	
	When sacrificing for my partner, I generally do so to increase intimacy in our relationship.	Impett Approach Avoidance Motives Scale	Impett et al. (2013)	
	When sacrificing for my partner, I generally do so to create more satisfaction in our relationship.	Impett Approach Avoidance Motives Scale	Impett et al. (2013)	
	When sacrificing for my partner, I generally do so to prevent my partner from feeling upset.	Impett Approach Avoidance Motives Scale	Impett et al. (2013)	
	When sacrificing for my partner, I generally do so to prevent my partner from feeling let down.	Impett Approach Avoidance Motives Scale	Impett et al. (2013)	
	When sacrificing for my partner, I generally do so to avoid conflict in our relationship.	Impett Approach Avoidance Motives Scale	Impett et al. (2013)	

	When sacrificing for my partner, I generally do so to avoid tension in our relationship.	Impett Approach Avoidance Motives Scale	Impett et al. (2013)	
29. Satisfaction	I feel competent and fully able to handle my relationship with my partner.	Marital Satisfaction Scale (MSS)	Roach et al. (1981)	✓
	I frequently enjoy pleasant conversations with my partner.	Marital Satisfaction Scale (MSS)	Roach et al. (1981)	✓
	I get along well with my partner.	Marital Satisfaction Scale (MSS)	Roach et al. (1981)	✓
	I have made a success of my relationship with my partner so far.	Marital Satisfaction Scale (MSS)	Roach et al. (1981)	✓
	My relationship with my partner helps me toward the goals I have set for myself.	Marital Satisfaction Scale (MSS)	Roach et al. (1981)	✓
	All things considered, I am very happy in my relationship with my partner.	Quality of Marriage Index (QMI)	Norton (1983)	✓
	I have a warm and comfortable relationship with my partner.	Couples Satisfaction Index (CSI-16)	Funk & Rogge (2007)	✓
	My relationship with my partner is rewarding.	Couples Satisfaction Index (CSI-16)	Funk & Rogge (2007)	✓
	My relationship with my partner is strong.	Couples Satisfaction Index (CSI-16)	Funk & Rogge (2007)	✓
	I have never regretted my relationship with my partner, not even for a moment.	ENRICH Marital Satisfaction Scale (EMS)	Fowers & Olson (1993)	✓
	My relationship with my partner is a perfect success.	ENRICH Marital Satisfaction Scale (EMS)	Fowers & Olson (1993)	✓
	I am satisfied with my partner.	Kansas Marital Satisfaction Scale (KMS)	Schumm et al. (1986)	✓
	My relationship with my partner is enjoyable.	Positive and Negative Semantic Differential (PN-SMD)	Mattson et al. (2013)	✓
	My partner and I have a better relationship than most couples I know.	Comprehensive Marital Satisfaction Scale (CMSS)	Blum & Mehrabian (1999)	✓
	My relationship with my partner is close to ideal.	Investment Model Scale	Rusbult et al. (1998)	✓
	I often feel angry or resentful toward my partner.	Braiker-Kelley Partnership Questionnaire	Braiker & Kelley (1979)	✓
	My relationship with my partner has been disappointing in several ways.	Marital Satisfaction Inventory (MSI)	Snyder (1979)	✓
	I become upset, angry, or irritable because of things that occur in the relationship.	Marital Satisfaction Scale (MSS)	Roach et al. (1981)	✓
	I get discouraged trying to make the relationship work out.	Marital Satisfaction Scale (MSS)	Roach et al. (1981)	✓
	My relationship with my partner is definitely unhappy.	Marital Satisfaction Scale (MSS)	Roach et al. (1981)	✓
	My relationship with my partner is not as good as most relationships.	Comprehensive Marital Satisfaction Scale (CMSS)	Blum & Mehrabian (1999)	✓
	I often wish I hadn't gotten into this relationship with my partner.	Relationship Assessment Scale (RAS)	Hendrick (1988)	✓
	My relationship with my partner is boring.	Positive and Negative Semantic Differential (PN-SMD)	Mattson et al. (2013)	✓
	My relationship with my partner is empty.	Positive and Negative Semantic Differential (PN-SMD)	Mattson et al. (2013)	✓
	My relationship with my partner is miserable.	Positive and Negative Semantic Differential (PN-SMD)	Mattson et al. (2013)	✓
30. Satisfaction (Perceived)	My partner is happy with our relationship.	Face-valid	Joel et al. (2020)	✓
	My partner thinks our relationship is strong.	Face-valid	--	✓
	My partner thinks we have a better relationship than most couples s/he knows.	Face-valid	--	✓
	My partner thinks we make a good team.	Face-valid	--	✓
	My partner thinks our relationship is close to ideal.	(adapted) Investment Model Scale (subscale)	Rusbult et al. (1998)	

	Our relationship does a good job of fulfilling my partner's needs for intimacy, companionship, etc.	(adapted) Investment Model Scale (subscale)	Rusbult et al. (1998)	
	My partner feels satisfied with our relationship.	(adapted) Investment Model Scale (subscale)	Rusbult et al. (1998)	
	My partner thinks our relationship is much better than others' relationships.	(adapted) Investment Model Scale (subscale)	Rusbult et al. (1998)	
	Our relationship makes my partner very happy.	(adapted) Investment Model Scale (subscale)	Rusbult et al. (1998)	
31. Self-disclosure	I always confide in my partner.	Comprehensive Marital Satisfaction Scale (CMSS)	Blum & Mehrabian (1999)	✓
	I have talked with my partner about my close relationships with other people.	Self-disclosure Index	Miller et al. (1983)	
	I have talked with my partner about my deepest feelings.	Self-disclosure Index	Miller et al. (1983)	
	I have talked with my partner about my personal habits.	Self-disclosure Index	Miller et al. (1983)	
	I have talked with my partner about my worst fears.	Self-disclosure Index	Miller et al. (1983)	
	I have talked with my partner about things I have done which I am proud of.	Self-disclosure Index	Miller et al. (1983)	
	I have talked with my partner about things I have done which I feel guilty about.	Self-disclosure Index	Miller et al. (1983)	
	I have talked with my partner about what I like and dislike about myself.	Self-disclosure Index	Miller et al. (1983)	
	I have talked with my partner about what is important to me in life.	Self-disclosure Index	Miller et al. (1983)	
	I have talked with my partner about what makes me the person I am.	Self-disclosure Index	Miller et al. (1983)	
32. Sexual Satisfaction	My sex life with my partner is fulfilling.	Quality of Sex Inventory	Shaw & Rogge (2016)	✓
	My sex life with my partner is very exciting.	Quality of Sex Inventory	Shaw & Rogge (2016)	✓
	Sex is fun for my partner and I.	Quality of Sex Inventory	Shaw & Rogge (2016)	✓
	My partner and I are sexually compatible.	Face-valid	--	✓
	My partner is willing to try new things in bed.	Face-valid	--	✓
	My partner is very sensitive to my sexual needs and desires.	Index of Sexual Satisfaction (ISS)	Hudson et al. (1981)	✓
	My partner enjoys our sex life.	Index of Sexual Satisfaction (ISS)	Hudson et al. (1981)	✓
	I am satisfied with our sexual relationship.	Quality of Sex Inventory (QSI-12)	Shaw & Rogge (2016)	✓
	I am happy with my partner as a lover.	Quality of Sex Inventory (QSI-12)	Shaw & Rogge (2016)	
	I am happy with my sex life with my partner.	Quality of Sex Inventory (QSI-12)	Shaw & Rogge (2016)	
	I am happy with the quality of sexual activity in our relationship.	Quality of Sex Inventory (QSI-12)	Shaw & Rogge (2016)	
	Sexual activity with my partner is fantastic.	Quality of Sex Inventory (QSI-12)	Shaw & Rogge (2016)	
	Sexual activity with my partner is rewarding.	Quality of Sex Inventory (QSI-12)	Shaw & Rogge (2016)	
	I do NOT enjoy sexual activity with my partner.	Quality of Sex Inventory (QSI-12)	Shaw & Rogge (2016)	✓
	Sexual activity with my partner leaves me empty.	Quality of Sex Inventory (QSI-12)	Shaw & Rogge (2016)	✓
	Sexual activity with my partner is a turn off.	Quality of Sex Inventory (QSI-12)	Shaw & Rogge (2016)	
	Sexual activity with my partner is not fun.	Quality of Sex Inventory (QSI-12)	Shaw & Rogge (2016)	
	Sexual activity with my partner is not worth the time or effort.	Quality of Sex Inventory (QSI-12)	Shaw & Rogge (2016)	
33. Social support	My partner is supportive of me when I have problems.	Frequency and Acceptability of Partner Behavior (adapted)	Doss & Christensen (2006)	✓
	I can count on my partner for help with a problem.	Quality of Relationships Inventory (QRI subscale)	Pierce et al. (1991)	
	I can count on my partner to give me honest feedback, even if I might not want to hear it.	Quality of Relationships Inventory (QRI subscale)	Pierce et al. (1991)	

	I can count on my partner to listen to me when I am very angry at something.	Quality of Relationships Inventory (QRI subscale)	Pierce et al. (1991)	
	I can count on my partner to me help me if my close family member died.	Quality of Relationships Inventory (QRI subscale)	Pierce et al. (1991)	
	I can really count on my partner to distract me from my worries when I feel under stress.	Quality of Relationships Inventory (QRI subscale)	Pierce et al. (1991)	
	I can turn to my partner for advice about problems.	Quality of Relationships Inventory (QRI subscale)	Pierce et al. (1991)	
	If I wanted to go out and do something, I am confident my partner would be willing to go with me.	Quality of Relationships Inventory (QRI subscale)	Pierce et al. (1991)	
34. Trust	I feel that my partner does not show me enough consideration.	Dyadic Trust Scale	Larzelere & Huston (1980)	✓
	My partner is primarily interested in their own welfare.	Dyadic Trust Scale	Larzelere & Huston (1980)	✓
	My partner treats me fairly and justly.	Dyadic Trust Scale	Larzelere & Huston (1980)	✓
	There are times when my partner cannot be trusted.	Dyadic Trust Scale	Larzelere & Huston (1980)	✓
	I can always trust my partner.	Marital Satisfaction Scale (MSS)	Roach et al. (1981)	✓
	I know what my partner expects of me in our relationship.	Marital Satisfaction Scale (MSS)	Roach et al. (1981)	✓
	My partner is dependable.	PRQC	Fletcher et al. (2000)	✓
	I can count on my partner.	PRQC	Fletcher et al. (2000)	
	I trust my partner.	PRQC	Fletcher et al. (2000)	

Note. For space considerations, we provide references for scales in the supplemental materials.
