# Supplementary material for: Predicting relationship quality with itself? A single general factor captures most of the variance across 34 common relationship measures
Source: PLoS One. 2026 Apr 1;21(4):e0342451. doi: 10.1371/journal.pone.0342451 (PMC13042769; doi:10.1371/journal.pone.0342451)
Supplement: S3 File — (PDF) [file pone.0342451.s003.pdf]

Appendix B

Study 2 Items Representing 34 Prominent Constructs in Relationship Science

	Construct	# items	Example item
1.	Affection	11	*My partner and I kiss daily.*
2.	Perceived Partner Affection	6	*My partner feels affection for me*
3.	Appreciation	10	*I appreciate my partner.*
4.	Perceived Partner Appreciation	14	*My partner makes sure I feel appreciated.*
5.	Partner-specific Attachment Anxiety	6	*I'm afraid my partner may abandon me.*
6.	Partner-specific Attachment Avoidance	6	*It helps to turn to my partner in times of need.*
7.	Capitalization	12	*My partner usually reacts to my good fortune enthusiastically.*
8.	Commitment	17	*I am committed to maintaining my relationship with my partner.*
9.	Perceived Partner Commitment	12	*My partner is committed to maintaining our relationship.*
10.	Communal strength	11	*I would be willing to give up a lot to benefit my partner.*
11.	Communication	6	*I tell my partner what I want or need from the relationship.*
12.	Conflict Frequency	12	*My partner and I argue with each other often.*
13.	Conflict Strategies	23	*When we have problems, my partner and I suggest possible solutions and compromises.*
14.	Empathy	10	*I try to look at my partner's side of a disagreement before I make a decision.*
15.	Goal Compatibility	16	*My partner and I share the same basic philosophy of life.*
16.	Inclusion of Other in Self	1	*Which of these circles best describes your relationship with your partner?*
17.	Intimacy	17	*I have a close relationship with my partner.*
18.	Intimacy (domain-specific)	18	*I share in many of my partner's interests.*
19.	Intimate Partner Violence	16	*In our relationship, I often insult or swear at my partner.*
20.	Investment	8	*I have invested a great deal into our relationship that I would lose if the relationship were to end.*
21.	Love	10	*I love my partner.*

22.	Normative Attachment	9	*My partner is the first person that I would turn to if I had a problem.*
23.	Partner Traits	21	*My partner is attractive.*
24.	Passion	13	*My relationship is passionate.*
25.	Perceived Partner Responsiveness	14	*My partner is responsive to my needs.*
26a.	Power (Felt)	9	*I can get my partner to listen to what I say.*
26b.	Power (Desired)	6	*I like to have power over my partner.*
27.	Quality of Alternatives	7	*The people other than my partner with whom I might become involved with are very appealing.*
28.	Sacrifice Motives	8	*When sacrificing for my partner, I generally do so to make my partner feel loved.*
29.	Satisfaction	25	*I am satisfied with my partner.*
30.	Perceived Partner Satisfaction	9	*My partner is happy with our relationship.*
31.	Self-disclosure	10	*I have talked with my partner about my deepest feelings.*
32.	Sexual Satisfaction	18	*My sex life with my partner is fulfilling.*
33.	Social Support	8	*My partner is supportive of me when I have problems.*
34.	Trust	9	*I trust my partner.*

Note. The table above lists the 34 relationship constructs measured in Study 2 (408 items total). The number of items per construct and example items are provided.
