# Supplementary material for: Predicting relationship quality with itself? A single general factor captures most of the variance across 34 common relationship measures
Source: PLoS One. 2026 Apr 1;21(4):e0342451. doi: 10.1371/journal.pone.0342451 (PMC13042769; doi:10.1371/journal.pone.0342451)
Supplement: S4 File — (PDF) [file pone.0342451.s004.pdf]

Appendix C

Full pattern matrix for Study 1 EFA and EBFA solution (206 items)

	3 correlated factors			Bifactor (G + 3 specific factors)			
Item	F1	F2	F3	General	SF1	SF2	SF3
I feel very lucky to have my partner in my life.	**.89**	.07	-.10	**.81**	.37	.00	-.04
I am committed to maintaining my relationship with my partner.	**.89**	.07	-.20	**.72**	**.52**	.03	-.01
I want our relationship to last a very long time.	**.88**	.04	-.21	**.68**	**.55**	.02	.01
My partner is one of the best people I know.	**.87**	.11	-.11	**.81**	.33	.04	-.08
My relationship with my partner is strong.	**.87**	.11	-.04	**.88**	.24	.02	-.09
I feel very attached to my partner.	**.87**	.02	-.09	**.75**	**.42**	-.03	.03
I really feel like part of a team with my partner.	**.86**	.05	-.01	**.86**	.20	-.04	-.08
I want to grow old with my partner.	**.86**	.12	-.22	**.71**	**.54**	.09	.00
The future of my relationship with my partner looks promising to me.	**.86**	.12	-.06	**.85**	.29	.04	-.05
All things considered, I am very happy in my relationship with my partner.	**.86**	.09	.03	**.88**	.28	.00	.02
My partner cares for me.	**.85**	.10	-.09	**.81**	.30	.03	-.09
I am satisfied with my partner.	**.85**	.10	.01	**.86**	.29	.01	.02
I have a close relationship with my partner.	**.85**	.07	.00	**.84**	.28	-.01	-.01
I appreciate my partner.	**.85**	.07	-.10	**.76**	**.40**	.02	.01
I want this relationship to stay strong no matter what rough times we may encounter.	**.84**	.09	-.21	**.68**	**.53**	.06	.00
My relationship with my partner is rewarding.	**.84**	.08	.04	**.87**	.23	-.01	-.01
I like to hang out with my partner.	**.84**	.04	.00	**.80**	.33	-.03	.05
I would not feel very upset if my relationship with my partner were to end in the near future.	**.83**	-.04	-.15	**.64**	**.47**	-.06	.02
My relationship with my partner is enjoyable.	**.83**	.08	.07	**.88**	.21	-.02	.02
My relationship with my partner is very stable.	**.83**	.17	-.07	**.85**	.22	.07	-.11
I love my partner.	**.83**	.07	-.14	**.69**	**.52**	.04	.08
My partner understands me.	**.82**	.06	.07	**.89**	.12	-.04	-.08
My partner knows me well.	**.82**	.03	-.06	**.76**	.30	-.03	-.04
My partner thinks we make a good team.	**.82**	.04	-.02	**.81**	.19	-.04	-.09
I get along well with my partner.	**.82**	.14	-.04	**.84**	.25	.05	-.06
I have made a success of my relationship with my partner so far.	**.82**	.09	-.01	**.84**	.20	-.01	-.08
I think my partner is dedicated to our relationship.	**.82**	.12	-.11	**.79**	.24	.04	-.13
My partner and I share many memories.	**.82**	-.03	-.15	**.65**	.39	-.06	-.05
I spend free time with my partner.	**.82**	-.01	-.01	**.76**	.26	-.07	-.03
I have a warm and comfortable relationship with my partner.	**.82**	.06	.04	**.84**	.19	-.03	-.05
I feel that my partner is genuine with me.	**.82**	.11	-.05	**.82**	.22	.03	-.09
I feel happy when I do something that helps my partner.	**.81**	-.03	-.08	**.67**	.42	-.06	.05
I think my partner is committed to maintaining our relationship.	**.81**	.10	-.09	**.77**	.26	.03	-.09
I adore my partner.	**.81**	.03	.05	**.79**	.32	-.04	.09
My life would seem empty without my relationship to my partner.	**.81**	.02	-.20	**.63**	**.45**	.00	-.04
My partner and I have a better relationship than most couples I know.	**.80**	-.02	.05	**.79**	.17	-.10	-.04
My partner has all the qualities I've ever wanted in a mate.	**.80**	.01	.10	**.83**	.20	-.07	.03
My partner listens to me when I need someone to talk to.	**.80**	.06	.06	**.86**	.10	-.05	-.10
I can always trust my partner.	**.80**	.13	-.14	**.76**	.25	.05	-.16
I frequently enjoy pleasant conversations with my partner.	**.80**	.05	.06	**.82**	.22	-.04	.02
My partner meets my needs.	**.80**	.04	.12	**.85**	.20	-.05	.07
I have meaningful conversations with my partner.	**.79**	.01	.07	**.81**	.16	-.07	-.03
My partner and I laugh together.	**.79**	.04	.05	**.80**	.24	-.04	.02
I know I'm valued and appreciated by my partner.	**.79**	.07	.11	**.88**	.10	-.04	-.05
During a discussion of a relationship issue or problem, my partner and I feel understood by each other.	**.79**	.08	.07	**.86**	.11	-.03	-.07
I am very happy about how we make decisions and resolve conflicts.	**.79**	.06	.10	**.88**	.06	-.05	-.09
My partner is happy with our relationship.	**.79**	.04	.10	**.85**	.12	-.06	-.03
My partner celebrates my good news.	**.79**	.08	.01	**.82**	.13	-.02	-.11
My partner thinks our relationship is strong.	**.78**	.05	.01	**.80**	.14	-.04	-.10
I feel competent and fully able to handle my relationship with my partner.	**.78**	.07	.01	**.80**	.18	-.01	-.06
My partner inspires me to do my best work.	**.78**	-.01	.13	**.82**	.15	-.10	.02
My partner and I agree on career decisions.	**.77**	.01	-.02	**.75**	.15	-.06	-.13
My partner is generally understanding.	**.77**	.10	.04	**.84**	.10	.00	-.12
My partner and I have similar ambitions and goals.	**.77**	-.01	.05	**.77**	.14	-.09	-.07
My relationship with my partner is close to ideal.	**.77**	.02	.17	**.86**	.10	-.09	.02
My partner wants our relationship to last forever.	**.77**	.02	-.13	**.66**	.29	-.03	-.10
My partner is friendly and warm toward me.	**.77**	.12	.07	**.85**	.14	.01	-.05
My partner and I share the same basic philosophy of life.	**.76**	.04	-.02	**.75**	.15	-.04	-.12
I always confide in my partner.	**.76**	-.04	.10	**.77**	.17	-.11	.01
My partner and I like playing together.	**.76**	-.01	.18	**.83**	.15	-.10	.09

Meeting the needs of my partner is a high priority for me.	**.76**	-.01	.03	**.72**	.28	-.07	.05
My partner respects me.	**.76**	.15	.03	**.86**	.09	.04	-.12
I know what my partner expects of me in our relationship.	**.76**	-.03	.07	**.75**	.15	-.11	-.03
My partner usually seems interested in doing things with me.	**.75**	.01	.14	**.81**	.11	-.09	.00
My relationship with my partner is a perfect success.	**.75**	-.02	.16	**.83**	.04	-.12	-.03
My partner and I agree on how we handle our finances.	**.75**	.02	-.05	**.72**	.11	-.06	-.18
My partner and I have fun together.	**.75**	.01	.13	**.79**	.18	-.08	.06
It is hard to imagine my life without my partner.	**.75**	.02	-.18	**.58**	**.44**	.00	-.01
My partner supports my career goals.	**.75**	.07	-.01	**.76**	.15	-.02	-.10
My partner and I make time to do fun things together.	**.74**	-.02	.18	**.82**	.08	-.11	.02
My partner and I agree on how to spend our leisure time.	**.74**	.03	.07	**.79**	.07	-.07	-.09
I tell my partner often that s/he is the best.	**.74**	-.03	.19	**.80**	.15	-.12	.09
I would be willing to give up a lot to benefit my partner.	**.74**	-.13	-.06	**.57**	.33	-.15	.01
My relationship with my partner helps me toward the goals I have set for myself.	**.74**	.00	.11	**.79**	.09	-.09	-.04
My partner thinks we have a better relationship than most couples s/he knows.	**.74**	-.05	.07	**.74**	.10	-.13	-.06
My partner makes me feel special.	**.74**	.01	.25	**.88**	.05	-.11	.07
I share in many of my partner's interests.	**.73**	-.12	.17	**.75**	.10	-.19	.03
My partner seems interested in what I am thinking and feeling.	**.73**	.00	.20	**.85**	.01	-.11	-.02
When I have a problem, I can talk to my partner about it.	**.73**	.09	.10	**.84**	.08	-.01	-.05
My partner is willing to make helpful improvements in our relationship.	**.73**	.01	.20	**.84**	.05	-.10	.01
My partner feels affection for me.	**.73**	.04	.16	**.81**	.13	-.06	.05
My partner is supportive of me when I have problems.	**.73**	.06	.05	**.78**	.08	-.04	-.10
My partner and I engage in outside interests together.	**.72**	-.02	.12	**.76**	.10	-.11	-.01
My partner helps me clarify my thoughts.	**.72**	-.10	.17	**.76**	.05	-.18	-.01
I make sure my partner feels appreciated.	**.72**	-.05	.13	**.73**	.21	-.11	.09
My partner regards me as an equal.	**.72**	.10	.01	**.77**	.08	.01	-.14
During a discussion of a relationship issue or problem, my partner and I express our feelings to each other.	**.71**	-.03	.26	**.84**	.06	-.13	.08
My partner treats me fairly and justly.	**.71**	.14	.01	**.79**	.09	.03	-.13
My partner is dependable.	**.71**	.09	-.17	**.64**	.24	.04	-.16
When we have problems, my partner and I suggest possible solutions and compromises.	**.71**	-.03	.17	**.80**	.00	-.12	-.06
My partner is responsive to my needs.	**.70**	.02	.25	**.85**	.08	-.08	.11
When we have problems, my partner and I try to discuss the problem.	**.70**	-.03	.19	**.79**	.04	-.12	.01
My partner and I eat together often.	**.70**	-.03	-.11	**.59**	.24	-.07	-.11
My partner and I work together on projects.	**.70**	-.06	.08	**.70**	.08	-.13	-.07
My partner and I agree on our dealings with our in-laws.	**.70**	-.01	-.06	**.63**	.17	-.06	-.12
My partner and I agree on household tasks.	**.69**	.00	.05	**.72**	.06	-.08	-.11
My partner expresses gratitude towards me often.	**.69**	.04	.25	**.87**	-.03	-.08	.00
My partner makes sure I feel appreciated.	**.69**	-.02	.20	**.80**	.00	-.12	.00
My partner and I settle our disagreements with mutual give and take.	**.68**	.02	.08	**.74**	.03	-.08	-.11
My partner and I try new things together.	**.68**	-.06	.30	**.81**	.04	-.15	.13
My partner often tells me s/he loves me.	**.67**	.00	.18	**.76**	.10	-.09	.07
When we have problems, my partner and I try to be especially nice to each other.	**.67**	-.07	.15	**.72**	.01	-.15	-.05
My partner is very loving and affectionate.	**.66**	-.01	.34	**.84**	.02	-.12	.16
Compared to other people I know, I have invested a great deal in my relationship with my partner.	**.66**	-.15	-.12	**.44**	.36	-.14	.01
My partner gives me sufficient opportunity to express my opinions.	**.65**	.05	.07	**.72**	.05	-.04	-.09
My partner often compliments me.	**.65**	-.02	.26	**.79**	.01	-.12	.06
I attend social events with my partner.	**.65**	-.04	.06	**.64**	.12	-.10	-.04
My partner and I agree on how children should be raised.	**.65**	-.02	-.04	**.60**	.12	-.08	-.12
I would go out of my way to do something for my partner.	**.64**	.05	-.24	**.45**	**.48**	.05	.00
My partner and I enjoy the same recreational activities.	**.64**	-.07	.16	**.69**	.05	-.14	.01
Many aspects of my life have become linked to my partner (recreational activities, etc.) and I would lose all of this if we were to break up.	**.64**	-.10	-.15	**.44**	.31	-.10	-.06
My partner and I often agree about major decisions.	**.64**	-.01	-.07	**.57**	.14	-.06	-.14
My partner and I like to hang out with our friends together.	**.64**	-.10	.13	**.64**	.07	-.16	-.01
I feel very good about how my partner and I practice our religious beliefs and values.	**.62**	-.04	.05	**.62**	.11	-.10	-.04
I have invested a great deal into our relationship that I would lose if the relationship were to end.	**.61**	-.09	-.18	.39	.35	-.09	-.06
My partner and I kiss daily.	**.60**	-.03	.27	**.71**	.12	-.11	.19
I have told my partner many private things about myself.	**.59**	-.11	.09	**.54**	.20	-.15	.09
I have never regretted my relationship with my partner, not even for a moment.	**.59**	.02	.04	**.60**	.13	-.04	-.02
My partner often tells me the things that s/he really likes about me.	**.59**	-.09	.38	**.76**	-.08	-.19	.12
My partner and I talk about the quality of our relationship often.	**.56**	-.18	.37	**.68**	-.07	-.26	.13
My partner and I enjoy spending time with other couples.	**.55**	-.11	.13	**.56**	.04	-.17	-.01
I tell my partner what I want or need from the relationship.	**.55**	-.16	.39	**.67**	.00	-.23	.22
My relationship is passionate.	**.54**	-.07	.52	**.79**	-.02	-.18	.34
Many of my partner's closest friends are also my closest friends.	**.51**	-.22	.17	**.49**	-.05	-.27	-.04
My partner and I are sexually compatible.	**.49**	-.06	.40	**.64**	.15	-.13	**.42**
My partner and I often discuss or consider separation or ending our relationship.	-.05	**.82**	-.15	.31	.12	**.70**	-.01

My relationship with my partner is miserable.	.11	**.78**	-.07	**.49**	.16	**.66**	.05
My partner makes unfair demands of my free time.	-.05	**.78**	-.05	.38	-.02	**.65**	-.04
When we have problems, my partner and I threaten one another with negative consequences.	-.08	**.78**	-.09	.32	.01	**.66**	-.04
I often feel angry or resentful toward my partner.	.07	**.77**	.04	**.53**	.00	**.62**	.03
During a discussion of a relationship issue or problem, my partner and I blame, accuse, and criticize one another.	.04	**.77**	.00	**.49**	-.03	**.62**	-.03
I often consider ending my relationship with my partner.	.13	**.77**	-.14	**.45**	.23	**.66**	.04
I feel that my partner disapproves of me.	.03	**.76**	.02	**.48**	-.02	**.62**	.00
When we have problems, my partner pushes, shoves, slaps, hits, or kicks me.	-.19	**.76**	-.13	.15	.08	**.67**	.01
My partner gets me badly flustered and jittery.	.02	**.75**	-.03	**.44**	-.04	**.61**	-.06
My partner thinks our relationship is in trouble.	.01	**.75**	-.07	.39	.06	**.63**	.00
When we have problems, my partner calls me names, swears at me, or attacks my character.	.01	**.75**	-.06	**.42**	-.05	**.61**	-.10
When we have problems, I call my partner names, swear at them, or attack their character.	-.17	**.75**	-.10	.20	.01	**.64**	-.03
My relationship with my partner is empty.	.14	**.75**	.04	**.57**	.10	**.60**	.10
Minor disagreements with my partner often end up in big arguments.	.09	**.74**	.03	**.55**	-.06	**.59**	-.05
I feel trapped in my relationship with my partner.	.14	**.74**	.00	**.54**	.16	**.61**	.10
There are times when my partner cannot be trusted.	.09	**.74**	-.12	**.45**	.05	**.61**	-.09
When we have problems, I push, shove, slap, hit, or kick my partner.	-.28	**.74**	-.13	.06	.05	**.66**	.01
I think my partner is unfaithful.	-.04	**.74**	-.11	.31	.05	**.63**	-.04
My partner is too flirtatious with other men/women.	-.09	**.74**	-.13	.26	.01	**.63**	-.08
I have discussed ending the relationship with friends and family members.	.06	**.73**	-.17	.35	.16	**.63**	-.04
My partner finds me rather dull and uninteresting.	-.07	**.73**	.13	**.44**	-.08	**.58**	.09
My relationship with my partner has been disappointing in several ways.	.15	**.72**	.09	**.62**	.01	**.56**	.06
My partner has seriously suggested the idea of ending the relationship.	-.03	**.72**	-.10	.32	.03	**.60**	-.04
I think my partner feels trapped in our relationship.	.02	**.71**	.05	**.46**	.02	**.58**	.08
My partner lacks respect for me.	.07	**.71**	-.01	**.49**	-.05	**.57**	-.07
My partner and I seem able to go for days sometimes without settling our differences.	.00	**.71**	-.05	.39	-.01	**.58**	-.05
There are times when my partner is dishonest with me.	.10	**.70**	-.07	**.47**	.00	**.57**	-.09
My relationship with my partner is definitely unhappy.	.22	**.69**	-.03	**.58**	.13	**.56**	.04
My partner is domineering.	-.01	**.69**	.01	**.41**	-.08	**.55**	-.06
I become upset, angry, or irritable because of things that occur in the relationship.	.03	**.69**	.10	**.51**	-.13	**.53**	-.02
My partner and I often argue about finances.	-.05	**.68**	-.01	.36	-.08	**.55**	-.06
I don't approve of the way my partner relates to my family.	-.01	**.68**	.00	.38	-.01	**.55**	.00
My partner and I argue with each other often.	-.01	**.68**	-.01	.39	-.06	**.55**	-.05
The future of my relationship with my partner is too uncertain to make serious plans.	.14	**.68**	-.15	**.41**	.17	**.57**	-.03
I feel neglected at times by my partner.	.09	**.68**	.21	**.62**	-.12	**.50**	.07
I get discouraged trying to make the relationship work out.	.17	**.67**	.02	**.56**	.05	**.53**	.02
I feel that my partner does not show me enough consideration.	.14	**.67**	.14	**.62**	-.09	**.50**	.02
My partner disapproves of some of my friends.	-.07	**.66**	-.07	.27	-.01	**.56**	-.05
My partner gets angry easily.	.05	**.66**	.12	**.53**	-.15	**.50**	-.03
My partner just tolerates or puts up with me.	-.04	**.66**	.00	.34	.00	**.54**	.03
My partner frequently tries to change my ideas.	-.04	**.66**	.03	.37	-.07	**.53**	-.01
I often feel distant from my partner.	.16	**.65**	.12	**.61**	-.02	**.50**	.06
My partner's habits annoy me.	.10	**.65**	.11	**.54**	-.02	**.50**	.07
My relationship with my partner is not as good as most relationships.	.19	**.64**	.07	**.59**	.06	**.50**	.08
I would enjoy living apart from my partner.	.13	**.64**	-.12	.38	.24	**.55**	.06
I worry a lot about my relationship with my partner.	.02	**.64**	.01	**.41**	-.06	**.51**	-.04
My partner and I don't have much in common to talk about.	.10	**.64**	.06	**.49**	.05	**.51**	.08
Sexual activity with my partner leaves me empty.	-.04	**.63**	.26	**.45**	.04	**.50**	.33
My partner doesn't notice when I do nice things for her/him.	.07	**.62**	.15	**.53**	-.10	**.47**	.04
I feel it is useless to discuss some things with my partner.	.07	**.62**	.21	**.57**	-.12	**.46**	.08
My partner and I differ on our general values and beliefs.	-.03	**.61**	-.11	.25	.05	**.52**	-.05
My partner is primarily interested in their own welfare.	.11	**.61**	-.03	**.45**	.01	**.48**	-.05
I prefer doing things without my partner.	.09	**.61**	.05	**.45**	.11	**.49**	.12
I often wish I hadn't gotten into this relationship with my partner.	.20	**.60**	-.08	**.46**	.22	**.51**	.06
My partner and I do not communicate well with each other.	.13	**.60**	.08	**.53**	-.04	**.46**	.01
My partner is jealous.	.03	**.58**	-.11	.31	.01	**.48**	-.11
I don't get the love and affection I want from my partner.	.07	**.58**	.27	**.57**	-.08	**.43**	.18
My partner is moody.	.07	**.58**	.14	**.51**	-.17	**.43**	-.02
My partner and I often get on each other's nerves.	.03	**.57**	.08	**.42**	-.09	**.45**	-.01
At times my partner takes me for granted.	.08	**.57**	.18	**.55**	-.15	**.42**	.02
My partner and I have problems in our relationship.	.18	**.57**	.01	**.52**	.00	**.44**	-.04
When we have problems, my partner and I avoid discussing the problem.	-.02	**.54**	.16	.39	-.08	**.41**	.10
My partner has feelings that are easily hurt.	-.17	**.51**	.15	.24	-.15	**.40**	.07
My relationship with my partner is boring.	.20	**.51**	.25	**.62**	.05	.37	.24
My partner and I have very few friends in common.	-.05	**.50**	-.01	.24	-.03	.41	-.02
My partner is critical.	-.07	**.50**	.09	.30	-.14	.39	-.02

I do NOT enjoy sexual activity with my partner.	.07	**.50**	.25	**.46**	.12	.39	.36
My partner drinks or uses drugs.	-.09	**.49**	-.08	.15	-.02	.41	-.07
My partner and I disagree on sexual matters.	-.03	**.47**	.36	**.44**	-.03	.35	.36
I feel our sexual activity is just routine.	-.10	**.47**	.35	.37	-.08	.36	.31
At times I take my partner for granted.	-.27	**.46**	.23	.15	-.15	.37	.19
My sex life with my partner is very exciting.	.34	-.18	**.63**	**.59**	-.05	-.26	**.50**
My sex life with my partner is fulfilling.	.42	-.08	**.63**	**.71**	-.01	-.18	**.52**
My partner enjoys our sex life.	.38	-.10	**.59**	**.65**	-.01	-.19	**.49**
Sex is fun for my partner and I.	.44	-.08	**.58**	**.69**	.08	-.17	**.56**
My partner is willing to try new things in bed.	.34	-.15	**.56**	**.56**	.00	-.22	**.48**
I am satisfied with our sexual relationship.	.46	-.06	**.54**	**.72**	.03	-.16	**.46**
My partner seems disinterested in sex.	-.15	.44	**.53**	**.40**	-.11	.31	**.51**
My partner is very sensitive to my sexual needs and desires.	.41	-.10	**.49**	**.62**	.00	-.18	.38
I am able to tell my partner when I want sexual intercourse.	.41	-.08	**.47**	**.61**	.07	-.15	**.43**

Note. Item loadings ≥ .40 on their primary factor are bolded to highlight representative items.
