# Supplementary material for: Predicting relationship quality with itself? A single general factor captures most of the variance across 34 common relationship measures
Source: PLoS One. 2026 Apr 1;21(4):e0342451. doi: 10.1371/journal.pone.0342451 (PMC13042769; doi:10.1371/journal.pone.0342451)
Supplement: S5 File — (PDF) [file pone.0342451.s005.pdf]

Appendix D

Full pattern matrix for Study 2 EFA and EBFA solution (408 items)

	3 correlated factors			Bifactor (G + 3 specific factors)			
Item	F1	F2	F3	General	SF1	SF2	SF3
My partner respects me.	**.78**	.07	.07	**.77**	.02	-.21	-.01
My partner regards me as an equal.	**.75**	.02	.04	**.72**	-.01	-.18	-.06
I know I'm valued and appreciated by my partner.	**.75**	.08	.05	**.75**	.00	-.19	-.01
My partner makes sure I feel appreciated.	**.73**	.10	.12	**.74**	.08	-.11	-.06
My partner values my abilities and opinions.	**.73**	.04	.14	**.66**	.08	-.21	-.01
My partner gives me sufficient opportunity to express my opinions.	**.73**	.06	.09	**.71**	.04	-.17	-.04
I can get my partner to listen to what I say.	**.73**	.03	.08	**.68**	.03	-.19	-.04
I'm afraid my partner may abandon me.	**-.73**	.45	.17	**-.42**	.23	.24	.36
My partner thinks our relationship is strong.	**.72**	.01	.09	**.66**	.04	-.16	-.08
My partner is generally understanding.	**.71**	.13	.09	**.74**	.04	-.18	.03
My partner is responsive to my needs.	**.70**	.13	.11	**.73**	.08	-.08	-.05
My partner is understanding.	**.69**	.10	.01	**.73**	-.03	-.19	.01
My partner treats me fairly and justly.	**.69**	.09	.02	**.73**	-.02	-.16	-.01
My partner really listens to me.	**.69**	.11	.10	**.71**	.06	-.10	-.04
My ideas and wishes are often ignored by partner.	**-.68**	-.09	.09	**-.79**	.10	.06	.10
My partner feels satisfied with our relationship.	**.68**	.09	.11	**.68**	.07	-.12	-.04
My partner thinks we make a good team.	**.68**	.13	.07	**.71**	.03	-.21	.07
I feel that my partner disapproves of me.	**-.68**	.01	.08	**-.69**	.11	.14	.10
My partner lacks respect for me.	**-.68**	.01	.10	**-.69**	.12	.16	.09
I can always trust my partner.	**.67**	.00	-.02	**.65**	-.07	-.22	-.03
I think my partner is dedicated to our relationship.	**.67**	.05	.02	**.67**	-.03	-.26	.05
My partner is happy with our relationship.	**.67**	.11	.11	**.68**	.07	-.13	-.01
My partner makes me feel special.	**.67**	.19	.14	**.73**	.10	-.11	.03
My partner seems interested in what I am thinking and feeling.	**.66**	.14	.10	**.70**	.06	-.12	.00
I feel that my partner is genuine with me.	**.66**	.06	-.01	**.68**	-.05	-.20	.01
My partner usually seems interested in doing things with me.	**.66**	.13	.12	**.68**	.07	-.15	.02
My partner understands me.	**.66**	.16	.11	**.71**	.07	-.15	.04
My partner is considerate.	**.65**	.10	.04	**.69**	.00	-.15	.00
I am very happy about how we make decisions and resolve conflicts.	**.65**	.08	.07	**.67**	.03	-.13	-.03
My partner is supportive of me when I have problems.	**.65**	.15	.08	**.70**	.04	-.20	.08
My partner values and respects the whole package that is the "real" me.	**.65**	.10	.06	**.68**	.02	-.16	.01
My partner expresses liking and encouragement for me.	**.65**	.15	.09	**.70**	.05	-.14	.03
My partner is friendly and warm toward me.	**.65**	.12	.01	**.70**	-.02	-.18	.04
Our relationship makes my partner very happy.	**.64**	.14	.05	**.71**	.02	-.08	-.03
My relationship with my partner is very stable.	**.64**	.04	-.03	**.65**	-.07	-.22	.02
During a discussion of a relationship issue or problem, my partner and I feel understood by each other.	**.64**	.07	.11	**.62**	.06	-.14	-.03
My partner is committed to maintaining our relationship.	**.64**	.05	-.01	**.64**	-.05	-.23	.03
I have a warm and comfortable relationship with my partner.	**.63**	.21	.03	**.76**	.01	-.13	.07
My partner feels affection for me.	**.63**	.26	.07	**.78**	.05	-.12	.10
My partner listens to me when I need someone to talk to.	**.63**	.15	.05	**.70**	.02	-.14	.04
My partner does not feel affection for me.	**-.62**	-.18	.00	**-.75**	.01	.06	.01
My relationship with my partner is close to ideal.	**.61**	.16	.08	**.70**	.05	-.05	-.03
In our relationship, my partner often shows respect for my feelings about an issue we disagree on.	**.61**	.07	.08	**.61**	.04	-.12	-.04
I feel that my partner does not show me enough consideration.	**-.61**	-.02	.07	**-.65**	.09	.08	.11
I often feel distant from my partner.	**-.61**	-.07	.02	**-.67**	.04	.00	.14
I feel that I can trust my partner completely.	**.61**	.02	-.04	**.62**	-.08	-.21	.01
There is a lot of conflict in my relationship with my partner.	**-.60**	.02	.15	**-.65**	.17	.10	.12
I can get my partner to do what I want.	**.60**	.01	.32	**.43**	.24	-.16	-.04
I really feel like part of a team with my partner.	**.60**	.14	.06	**.66**	.03	-.13	.03
My wishes do not carry much weight in my romantic relationship.	**-.60**	-.03	.14	**-.69**	.14	.03	.14
My partner expresses gratitude towards me often.	**.60**	.15	.14	**.64**	.10	-.07	-.02
My partner often tells me s/he loves me.	**.59**	.22	.12	**.69**	.10	-.08	.05
My partner and I have a lot of disagreements.	**-.59**	.11	.13	**-.55**	.15	.09	.19

My partner thinks our relationship is in trouble.	**-.59**	.10	.17	**-.56**	.19	.17	.12
I think my partner is committed to maintaining our relationship.	**.59**	.08	-.04	**.63**	-.07	-.24	.08
I think I have a great deal of power in my romantic relationship.	**.59**	.01	.49	.33	**.40**	-.14	-.04
I feel neglected at times by my partner.	**-.58**	.03	.04	**-.58**	.06	.02	.19
My relationship with my partner is strong.	**.58**	.23	.02	**.73**	.00	-.14	.11
I get along well with my partner.	**.58**	.21	.00	**.72**	-.01	-.13	.09
I worry a lot about my relationship with my partner.	**-.58**	.25	.19	**-.46**	.22	.12	.27
My partner often tells me the things that s/he really likes about me.	**.58**	.19	.23	**.61**	.20	-.01	-.03
I can count on my partner to listen to me when I am very angry at something.	**.58**	.04	.00	**.59**	-.03	-.14	-.03
I trust my partner.	**.57**	.07	-.09	**.64**	-.11	-.19	.03
I can count on my partner for help with a problem.	**.57**	.09	.00	**.61**	-.03	-.18	.04
My relationship with my partner is not as good as most relationships.	**-.57**	-.10	.04	**-.66**	.05	.05	.06
I feel competent and fully able to handle my relationship with my partner.	**.56**	.10	.06	**.59**	.03	-.13	.01
All things considered, I am very happy in my relationship with my partner.	**.56**	.24	-.03	**.75**	-.04	-.09	.08
My partner is willing to make helpful improvements in our relationship.	**.56**	.19	.07	**.65**	.05	-.10	.05
My partner celebrates my good news.	**.56**	.12	.05	**.60**	.02	-.16	.05
My partner is kind.	**.56**	.14	.01	**.64**	-.02	-.16	.07
My partner is very loving and affectionate.	**.56**	.24	.08	**.69**	.07	-.03	.03
Minor disagreements with my partner often end up in big arguments.	**-.56**	.10	.18	**-.55**	.19	.07	.19
My partner thinks our relationship is much better than others' relationships.	**.55**	.03	.12	**.51**	.08	-.11	-.05
I often worry about whether my partner really cares for me.	**-.55**	.17	.18	**-.49**	.20	.10	.21
The future of my relationship with my partner looks promising to me.	**.55**	.25	.00	**.73**	-.01	-.12	.12
My partner intends to stay in this relationship.	**.55**	-.03	.00	**.50**	-.05	-.24	.01
My partner is oriented toward the long-term future of our relationship (for example, imagines being with me several years from now).	**.55**	.01	-.05	**.54**	-.08	-.23	.03
I feel that I can confide in my partner about virtually everything.	**.55**	.19	.02	**.67**	.00	-.12	.07
In our relationship, my partner often insults me or swears at me.	**-.54**	.08	.17	**-.54**	.19	.13	.13
My relationship with my partner is definitely unhappy.	**-.54**	-.13	.13	**-.70**	.13	.08	.00
My partner and I settle our disagreements with mutual give and take.	**.54**	.05	.05	**.53**	.02	-.14	-.02
My partner and I have problems in our relationship.	**-.54**	.05	.08	**-.54**	.10	.03	.18
When we have problems, my partner and I suggest possible solutions and compromises.	**.54**	.17	.11	**.60**	.08	-.10	.05
My relationship with my partner has been disappointing in several ways.	**-.54**	-.15	.08	**-.69**	.08	.01	.05
When we have problems, my partner and I try to discuss the problem.	**.54**	.15	.07	**.61**	.04	-.11	.04
If I want to, I get to make decisions in my romantic relationship.	**.53**	.15	.18	**.55**	.15	-.07	.01
My views have little sway, even if I voice them to my partner.	**-.53**	.10	.21	**-.54**	.22	.14	.13
I worry that my partner won't care about me as much as I care about them.	**-.53**	.20	.14	**-.43**	.17	.10	.23
I get discouraged trying to make the relationship work out.	**-.53**	-.03	.15	**-.61**	.15	.05	.10
I can count on my partner.	**.53**	.20	.01	**.65**	-.02	-.18	.14
When I tell my partner about something good that has happened to me, I sometimes get the impression that he/she doesn't care that much.	**-.53**	-.06	.09	**-.60**	.10	.06	.08
I am not able to get my way with my partner, even when I try.	**-.52**	.07	.09	**-.50**	.11	.08	.15
When I have a problem, I can talk to my partner about it.	**.52**	.18	-.01	**.65**	-.03	-.12	.08
I have a close relationship with my partner.	**.52**	.29	.03	**.72**	.02	-.10	.14
My partner meets my needs.	**.52**	.30	.04	**.73**	.04	.00	.06
During a discussion of a relationship issue or problem, my partner and I blame, accuse, and criticize one another.	**-.52**	.07	.18	**-.54**	.19	.08	.16
When I tell my partner about something good that has happened to me, my partner doesn't pay much attention to me.	**-.52**	-.06	.14	**-.62**	.15	.09	.05
I am satisfied with my partner.	**.52**	.29	.01	**.73**	.01	-.05	.10
My partner and I make time to do fun things together.	**.52**	.26	.11	**.66**	.10	-.03	.05
My partner supports my career goals.	**.52**	.08	.04	**.53**	.01	-.15	.02
My relationship with my partner is rewarding.	**.51**	.31	.00	**.74**	-.01	-.09	.14
My partner cares for me.	**.51**	.15	.00	**.60**	-.02	-.19	.12
I feel like all my partner and I do is fight.	**-.51**	.09	.25	**-.54**	.26	.16	.10
My partner feels very attached to our relationship -- strongly linked to me.	**.51**	.14	.04	**.58**	.01	-.15	.07
I think my partner feels trapped in our relationship.	**-.51**	.03	.13	**-.54**	.14	.08	.11
I have made a success of my relationship with my partner so far.	**.51**	.19	-.02	**.65**	-.03	-.12	.08
The future of my relationship with my partner is too uncertain to make serious plans.	**-.51**	.05	.22	**-.55**	.23	.19	.04
I find it easy to depend on my partner.	**.51**	.10	-.02	**.57**	-.04	-.14	.03
At times my partner takes me for granted.	**-.51**	.04	.07	**-.52**	.08	-.01	.20

My partner thinks we have a better relationship than most couples s/he knows.	**.51**	.14	.09	**.55**	.06	-.11	.03
My partner and I do not communicate well with each other.	**-.50**	-.09	.06	**-.59**	.07	.06	.04
My partner doesn't notice when I do nice things for her/him.	**-.50**	.03	.08	**-.49**	.10	.12	.08
My partner "gets the facts right" about me.	**.50**	.16	.08	**.57**	.05	-.09	.04
My partner and I have a better relationship than most couples I know.	**.49**	.19	.07	**.59**	.05	-.08	.06
My partner is "on the same wavelength" with me.	**.49**	.20	-.07	**.67**	-.07	-.06	.04
My partner often compliments me.	**.49**	.18	.08	**.59**	.07	.01	-.03
My partner sees the "real" me.	**.49**	.20	.03	**.61**	.02	-.12	.10
My relationship with my partner is a perfect success.	**.49**	.19	.13	**.58**	.11	.00	-.01
I don't get the love and affection I want from my partner.	**-.49**	-.16	.06	**-.65**	.05	-.03	.06
My partner gets angry easily.	**-.49**	.14	.15	**-.44**	.16	.08	.19
When I tell my partner about something good that has happened to me, my partner usually reacts to my good fortune enthusiastically.	**.49**	.22	.03	**.63**	.01	-.09	.09
My relationship with my partner helps me toward the goals I have set for myself.	**.48**	.23	.05	**.61**	.04	-.11	.11
My relationship with my partner is enjoyable.	**.48**	.33	-.02	**.74**	-.01	-.03	.13
My relationship with my partner is miserable.	**-.48**	-.14	.23	**-.69**	.22	.09	-.03
I often consider ending my relationship with my partner.	**-.48**	-.14	.21	**-.67**	.20	.10	-.04
My partner thinks our relationship is close to ideal.	**.48**	.14	.09	**.54**	.07	-.03	-.02
When we have problems, my partner calls me names, swears at me, or attacks my character.	**-.48**	.09	.25	**-.51**	.26	.14	.10
My partner and I agree on how we handle our finances.	**.48**	.11	.05	**.52**	.03	-.07	-.01
My partner wants our relationship to last forever.	**.48**	.15	.02	**.55**	-.01	-.18	.12
I am often irritated by my partner.	**-.47**	-.07	.11	**-.58**	.10	-.02	.12
My relationship with my partner is empty.	**-.47**	-.26	.14	**-.73**	.12	.02	-.07
When I tell my partner about something good that has happened to me, my partner often seems disinterested.	**-.47**	-.02	.16	**-.55**	.17	.06	.08
My partner has seriously suggested the idea of ending the relationship.	**-.47**	.13	.23	**-.45**	.24	.18	.09
I am connected to my partner.	**.47**	.29	.03	**.66**	.02	-.10	.16
During a discussion of a relationship issue or problem, my partner criticizes while I defend myself.	**-.47**	.07	.18	**-.49**	.19	.05	.16
There are times when my partner cannot be trusted.	**-.46**	.00	.21	**-.54**	.21	.13	.04
My partner inspires me to do my best work.	**.45**	.31	.09	**.64**	.08	-.04	.13
My partner knows me well.	**.45**	.23	.04	**.59**	.02	-.16	.16
During a discussion of a relationship issue or problem, my partner and I express our feelings to each other.	**.45**	.20	.07	**.56**	.05	-.06	.06
I think my partner is unfaithful.	**-.44**	.02	.14	**-.47**	.16	.17	.00
My partner helps me clarify my thoughts.	**.44**	.26	.14	**.57**	.12	-.06	.10
My relationship with my partner is close.	**.44**	.29	-.03	**.67**	-.03	-.08	.14
I frequently enjoy pleasant conversations with my partner.	**.44**	.25	.02	**.61**	.02	-.08	.11
When we have problems, my partner and I try to be especially nice to each other.	**.44**	.12	.09	**.47**	.07	-.09	.02
My partner and I often discuss or consider separation or ending our relationship.	**-.44**	.00	.22	**-.52**	.22	.13	.02
During a discussion of a relationship issue or problem, I try to start a discussion while my partner tries to avoid a discussion.	**-.44**	.02	.11	**-.47**	.12	.03	.13
I can really count on my partner to distract me from my worries when I feel under stress.	**.43**	.26	.04	**.61**	.04	.00	.06
If I wanted to go out and do something, I am confident my partner would be willing to go with me.	**.43**	.15	.03	**.52**	.01	-.09	.05
It helps to turn to my partner in times of need.	**.43**	.25	-.01	**.61**	-.01	-.10	.14
My partner gets me badly flustered and jittery.	**-.43**	.15	.16	-.38	.18	.09	.17
I am not afraid about being abandoned by my partner.	**.43**	-.37	-.16	.19	-.20	-.17	-.27
I feel it is useless to discuss some things with my partner.	**-.43**	-.06	.13	**-.53**	.12	.00	.10
My partner and I agree on career decisions.	**.42**	.11	.06	**.47**	.04	-.11	.04
I can turn to my partner for advice about problems.	**.42**	.25	.00	**.60**	-.01	-.13	.16
My partner and I agree on how to spend our leisure time.	**.42**	.17	.02	**.54**	.01	-.05	.04
I often make my partner laugh.	**.42**	.23	.10	**.54**	.08	-.08	.10
Our relationship does a good job of fulfilling my partner's needs for intimacy, companionship, etc.	**.42**	.25	.07	**.59**	.08	.10	-.03
I feel very lucky to have my partner in my life.	**.42**	.35	.01	**.66**	.01	-.11	.23
My partner and I argue with each other often.	**-.42**	.11	.26	**-.45**	.25	.04	.18

My partner is dependable.	**.42**	.10	-.12	**.52**	-.13	-.16	.08
When I tell my partner about something good that has happened to me, I sometimes get the sense that my partner is even more happy and excited than I am.	**.42**	.20	.13	**.51**	.11	.01	.01
My partner and I like playing together.	**.41**	.35	.15	**.61**	.14	.00	.13
My partner is successful.	**.41**	.19	.05	**.53**	.04	-.01	.03
My partner rarely tells me s/he loves me.	**-.41**	-.15	.01	**-.53**	.01	.04	-.02
My partner is moody.	**-.41**	.03	.15	**-.45**	.15	.04	.13
There are times when my partner is dishonest with me.	**-.41**	.08	.20	**-.44**	.20	.07	.13
I often wish I hadn't gotten into this relationship with my partner.	**-.41**	-.22	.14	**-.64**	.13	.06	-.09
I can count on my partner to me help me if my close family member died.	**.41**	.08	-.07	**.47**	-.09	-.21	.10
I have meaningful conversations with my partner.	**.41**	.34	.02	**.65**	.02	-.05	.17
When we have problems, my partner and I threaten one another with negative consequences.	**-.41**	.03	.24	**-.50**	.23	.01	.14
My partner and I often get on each other's nerves.	**-.41**	-.04	.23	**-.56**	.21	-.03	.12
My partner makes unfair demands of my free time.	**-.41**	.02	.24	**-.50**	.23	.07	.09
When I tell my partner about something good that has happened to me, my partner often asks a lot of questions and shows genuine concern about the good event.	**.41**	.10	.10	**.41**	.07	-.13	.06
My partner and I work together on projects.	**.40**	.22	.00	**.56**	-.01	-.06	.09
I usually talk things over with my partner.	**.40**	.24	-.01	**.57**	-.01	-.09	.13
My partner and I share the same basic philosophy of life.	**.40**	.12	-.06	**.50**	-.07	-.12	.07
In our relationship, my partner often explains their side or suggests a compromise.	**.40**	.06	.06	**.40**	.04	-.05	-.04
I can count on my partner to give me honest feedback, even if I might not want to hear it.	**.40**	.15	.00	**.50**	-.01	-.09	.07
My partner is domineering.	**-.40**	.09	.21	**-.41**	.21	.08	.13
It is rare that my partner and I get in a big argument.	.39	-.04	-.17	**.43**	-.17	-.08	-.09
I feel trapped in my relationship with my partner.	-.39	-.23	.19	**-.66**	.17	.01	-.05
I have discussed ending the relationship with friends and family members.	-.39	.00	.28	**-.50**	.26	.10	.04
My partner and I often argue about finances.	-.39	.10	.21	**-.41**	.21	.05	.15
When I tell my partner about something good that has happened to me, my partner often finds problems with it.	-.38	-.04	.22	**-.50**	.21	.09	.02
My partner is financially secure.	.38	-.02	.02	.35	-.01	-.08	-.07
I become upset, angry, or irritable because of things that occur in the relationship.	-.38	.01	.18	**-.47**	.17	-.04	.16
I often feel angry or resentful toward my partner.	-.38	-.16	.21	**-.61**	.18	-.01	.02
My partner and I agree on household tasks.	.38	.10	.06	**.42**	.04	-.05	.00
My partner finds me rather dull and uninteresting.	-.38	-.16	.07	**-.53**	.07	.01	-.01
My partner and I kiss daily.	.38	.26	.05	**.56**	.06	.04	.04
My partner and I like to hang out with our friends together.	.37	.15	.05	**.46**	.04	-.02	.01
My partner and I have fun together.	.37	.37	.00	**.65**	.01	-.03	.18
I know what my partner expects of me in our relationship.	.37	.16	.02	**.48**	.01	-.06	.05
My partner and I engage in outside interests together.	.37	.21	.05	**.50**	.05	-.03	.06
My partner is one of the best people I know.	.37	.35	-.01	**.64**	-.01	-.07	.21
My partner is too flirtatious with other men/women.	-.36	.12	.21	-.35	.22	.15	.08
My partner esteems me, shortcomings and all.	.36	.03	-.06	**.40**	-.07	-.10	.00
My partner and I have similar ambitions and goals.	.36	.23	-.03	**.54**	-.03	-.07	.12
My partner and I often agree about major decisions.	.36	.12	-.09	**.48**	-.09	-.08	.04
My partner is the first person that I would turn to if I had a problem.	.35	.29	-.10	**.60**	-.09	-.10	.19
When we have problems, my partner and I avoid discussing the problem.	-.34	-.13	.09	**-.49**	.08	.00	.02
My partner is primarily interested in their own welfare.	-.34	-.04	.27	**-.49**	.25	.10	.01
I always confide in my partner.	.34	.33	-.01	**.60**	.00	.01	.13
My partner and I are always in agreement on major issues.	.33	.06	.07	.34	.05	-.05	-.02
I don't feel comfortable opening up to my partner.	-.32	-.13	.06	**-.45**	.05	.02	-.01
I spend free time with my partner.	.32	.30	-.01	**.54**	-.01	-.09	.20
I need a lot of reassurance that I am loved by my partner.	-.31	.27	.16	-.17	.18	.09	.23
My partner and I agree on how children should be raised.	.31	.17	.03	**.42**	.03	-.05	.07
I attend social events with my partner.	.31	.12	.01	**.40**	.00	-.05	.03
My partner and I seem able to go for days sometimes without settling our differences.	-.31	-.02	.29	**-.47**	.27	.01	.08
My partner just tolerates or puts up with me.	-.31	.01	.24	**-.42**	.22	.00	.11

My partner and I agree on our dealings with our in-laws.	.31	.09	-.05	**.40**	-.05	-.05	.01
My partner's habits annoy me.	-.29	-.20	.07	**-.49**	.04	-.10	.03
My partner and I eat together often.	.29	.22	.02	**.44**	.02	-.04	.10
My partner is critical.	-.28	.02	.12	-.33	.11	-.05	.15
Many of my partner's closest friends are also my closest friends.	.28	.07	.09	.29	.07	-.03	-.01
My partner and I enjoy spending time with other couples.	.28	.09	.06	.31	.04	-.07	.03
If I achieved something good, my partner is the person that I would tell first.	.28	.28	-.13	**.54**	-.11	-.11	.21
I don't approve of the way my partner relates to my family.	-.28	-.10	.19	**-.44**	.17	.03	.00
My partner and I don't have much in common to talk about.	-.28	-.22	.09	**-.50**	.07	-.01	-.06
During a discussion of a relationship issue or problem, my partner pressures, nags, or demands while I withdraw, become silent, or refuse to discuss further.	-.28	-.01	.27	**-.41**	.25	.04	.06
I get extremely depressed when things don't go right in my relationship with my partner.	-.27	.18	.10	-.19	.12	.02	.20
I feel very good about how my partner and I practice our religious beliefs and values.	.27	.23	.00	**.45**	.01	.01	.08
My partner dissaproves of some of my friends.	-.27	.05	.20	-.31	.20	.10	.04
Which of these circles best describes your relationship with your partner?	.25	.20	-.05	**.42**	-.04	-.02	.09
My partner is jealous.	-.25	.05	.24	-.31	.23	.08	.05
My partner drinks or uses drugs.	-.25	.12	.15	-.21	.16	.09	.09
Sometimes I don't feel very sorry for my partner when he/she is having problems.	-.25	-.14	.17	**-.44**	.14	-.02	-.01
My partner has a good job.	.24	.12	.02	.32	.01	.00	.01
I often share physical affection (outside sex) with my partner.	.23	.21	.01	.38	.02	-.02	.10
My partner sometimes says that I fail to notice the nice things that s/he does for me.	-.23	-.01	.18	-.33	.17	-.04	.11
My partner is outgoing.	.19	.18	.12	.27	.12	.04	.04
My partner and I have very few friends in common.	-.18	-.04	.11	-.26	.10	-.01	.04
My partner is sexy.	-.09	**.67**	-.04	**.50**	.05	.33	.24
Sexual activity with my partner is rewarding.	.07	**.62**	.02	**.60**	.10	.37	.13
Sexual activity with my partner is fantastic.	.14	**.59**	.11	**.61**	.19	**.43**	.05
I want my partner physically, emotionally, mentally.	.02	**.59**	-.07	**.52**	-.01	.09	.36
Sex is fun for my partner and I.	.15	**.59**	.06	**.63**	.14	**.40**	.06
My partner is attractive.	-.04	**.58**	-.10	**.48**	-.02	.18	.28
I love my partner.	-.02	**.57**	-.18	**.51**	-.11	.04	.39
My sex life with my partner is very exciting.	.13	**.56**	.13	**.58**	.20	**.46**	.00
I do NOT enjoy sexual activity with my partner.	.05	**-.56**	.10	**-.48**	.01	-.31	-.16
I would rather be with my partner than anyone else.	.09	**.55**	-.16	**.59**	-.09	.07	.31
My partner and I are sexually compatible.	.04	**.54**	-.03	**.53**	.05	.34	.11
I adore my partner.	.17	**.54**	-.01	**.60**	.03	.06	.30
I want my relationship with my partner to last forever.	.03	**.54**	-.19	**.54**	-.13	.01	.38
My partner always seems to be on my mind.	-.01	**.54**	.03	**.41**	.08	.17	.25
My sex life with my partner is fulfilling.	.16	**.53**	.04	**.61**	.12	**.40**	.02
It is hard to imagine my life without my partner.	.06	**.53**	-.14	**.53**	-.08	.03	.35
My partner has a nice body.	-.04	**.52**	-.05	**.43**	.02	.27	.17
I am happy with my sex life with my partner.	.20	**.52**	.06	**.64**	.13	**.42**	-.02
I have an endless appetite for affection from my partner.	-.05	**.51**	-.01	.37	.05	.15	.27
I cherish my partner.	.14	**.51**	-.13	**.60**	-.08	.02	.31
I feel very attached to my partner.	.12	**.51**	-.08	**.56**	-.03	.04	.30
Meeting the needs of my partner is a high priority for me.	.05	**.51**	-.05	**.47**	.00	.07	.29
Sexual activity with my partner is not worth the time or effort.	-.11	**-.51**	.11	**-.59**	.04	-.27	-.12
I am happy with my partner as a lover.	.21	**.50**	.02	**.64**	.08	.28	.07
I possess a powerful attraction for my partner.	.09	**.50**	-.03	**.50**	.02	.10	.25
I would do almost anything for my partner.	.01	**.49**	-.12	**.45**	-.07	.01	.35
Sexual activity with my partner is a turn off.	-.04	**-.49**	.19	**-.55**	.11	-.23	-.16
I like to hang out with my partner.	.12	**.49**	-.08	**.54**	-.03	.00	.31
I tell my partner often that s/he is the best.	.18	**.48**	.02	**.56**	.06	.11	.20
I want our relationship to last a very long time.	.13	**.48**	-.16	**.57**	-.12	-.03	.34
I would be willing to go far to visit my partner.	.10	**.48**	-.08	**.51**	-.04	.01	.32
It is important to me to see or talk with my partner regularly.	.03	**.48**	-.14	**.47**	-.09	.01	.33
I often tell my partner how much I appreciate her/him.	.15	**.48**	-.03	**.55**	.01	.09	.22
Sexual activity with my partner is not fun.	-.11	**-.48**	.09	**-.57**	.02	-.30	-.07
I want to grow old with my partner.	.12	**.48**	-.18	**.57**	-.13	.00	.31

Sexual activity with my partner leaves me empty.	-.12	**-.47**	.12	**-.59**	.04	-.31	-.06
My partner is willing to try new things in bed.	.01	**.46**	.11	.36	.16	.29	.10
I want this relationship to stay strong no matter what rough times we may encounter.	.09	**.46**	-.16	**.51**	-.11	-.04	.34
I care about my partner.	.04	**.45**	-.15	**.46**	-.10	-.02	.34
My relationship with my partner is intimate.	.31	**.45**	.04	**.67**	.08	.19	.08
I am committed to maintaining my relationship with my partner.	.14	**.45**	-.14	**.55**	-.10	-.02	.30
I often approve of or compliment my partner.	.10	**.45**	-.07	**.50**	-.03	.06	.24
I often say "I love you" to my partner.	.12	**.45**	-.02	**.49**	.02	.09	.22
My relationship is passionate.	.37	**.45**	.13	**.68**	.16	.20	.05
I feel happy when I do something that helps my partner.	.03	**.44**	-.15	**.45**	-.10	.04	.27
I would go out of my way to do something for my partner.	-.03	**.44**	-.22	**.43**	-.16	.05	.29
My relationship is sexually intense.	.05	**.44**	.18	.36	.23	.36	.02
I would be willing to give up a lot to benefit my partner.	-.08	**.44**	-.07	.29	-.03	.01	.33
I often do something nice for my partner.	-.01	**.44**	-.10	.38	-.04	.09	.24
I feel very attached to our relationship--very strongly linked to my partner.	.22	**.43**	-.11	**.61**	-.07	-.01	.25
I make sure my partner feels appreciated.	.14	**.43**	-.05	**.50**	-.01	.03	.24
I make an effort to stay in contact with my partner.	.09	**.43**	-.05	**.44**	-.02	-.02	.30
I am satisfied with our sexual relationship.	.24	**.42**	.07	**.59**	.13	.36	-.06
I have talked with my partner about my deepest feelings.	.15	**.42**	.05	**.46**	.07	.04	.22
When I am away from my partner, I feel down.	-.06	**.41**	.08	.23	.12	.09	.25
My life would seem empty without my relationship to my partner.	.02	**.41**	-.18	**.42**	-.13	-.01	.30
I have talked with my partner about what I like and dislike about myself.	.07	**.40**	.05	.36	.08	.07	.22
I would feel deep despair if my partner left me.	-.05	**.40**	-.19	.35	-.14	-.01	.31
I have told my partner many private things about myself.	.09	.39	-.03	**.41**	.01	.04	.23
I am oriented toward the long-term future of my relationship (for example, I imagine being with my partner several years from now).	.15	.39	-.20	**.54**	-.16	-.09	.31
I am happy with the quality of sexual activity in our relationship.	.28	.39	.13	**.57**	.17	.32	-.05
My needs for intimacy and companionship could NOT easily be fulfilled in an alternative relationship.	-.02	.39	-.24	**.41**	-.16	.11	.20
I appreciate my partner.	.15	.38	-.17	**.53**	-.12	.00	.23
I prefer doing things without my partner.	.00	-.38	.21	**-.42**	.15	-.12	-.17
I have talked with my partner about what is important to me in life.	.17	.38	.02	**.45**	.04	.00	.22
My partner enjoys our sex life.	.29	.37	.12	**.55**	.15	.23	.00
I sometimes try to understand my partner better by imagining how things look from his/her perspective.	-.02	.37	-.03	.29	.01	.08	.20
I would be likely to give my partner large benefits.	.08	.37	-.03	.38	-.01	-.01	.25
I would greatly enjoy being confided in by my partner.	-.01	.37	-.10	.32	-.05	.01	.27
My partner has all the qualities I've ever wanted in a mate.	.31	.37	.05	**.58**	.06	.06	.13
I often share emotions, feelings, or problems with my partner.	.32	.37	.05	**.57**	.06	-.04	.20
My life would be severely disrupted if my partner was no longer a part of it.	-.04	.36	-.30	.38	-.24	-.03	.30
My relationship with my partner is boring.	-.31	-.36	.10	**-.66**	.06	-.13	-.06
Compared to other people I know, I have invested a great deal in my relationship with my partner.	-.03	.35	-.06	.27	-.02	.03	.24
I am able to tell my partner when I want sexual intercourse.	.20	.35	.05	**.48**	.08	.20	.03
My partner is the person that I would want to go to, to help me feel better when something bad happens to me or I feel upset.	.23	.35	-.11	**.55**	-.09	-.04	.21
I usually discuss my problems and concerns with my partner.	.28	.34	-.01	**.54**	.00	-.05	.20
I share in many of my partner's interests.	.22	.34	.00	**.49**	.02	.01	.17
I have talked with my partner about my personal habits.	.09	.34	.02	**.34**	.04	-.01	.23
My partner dresses well.	.15	.34	.07	**.40**	.10	.14	.09
My partner and I try new things together.	.31	.34	.04	**.57**	.06	.09	.08
I acknowledge the things that my partner does for me, even the really small things.	.06	.34	-.07	.36	-.03	.02	.21
My partner and I enjoy the same recreational activities.	.15	.33	.03	**.40**	.05	.06	.14
I have talked with my partner about things I have done which I feel guilty about.	.01	.33	.00	.27	.03	.05	.19
I tell my partner what I want or need from the relationship.	.20	.33	.14	.39	.15	.03	.15
Sometimes I don't really acknowledge or treat my partner like s/he is someone special.	-.03	-.33	.17	-.39	.11	-.18	-.07
My partner and I laugh together.	.32	.33	-.01	**.57**	.00	-.07	.20
My relationship is lustful.	-.01	.32	.06	.25	.10	.21	.07

I have talked with my partner about my worst fears.	.14	.32	.00	.39	.02	.00	.19
I have talked with my partner about what makes me the person I am.	.18	.32	.07	**.40**	.08	.07	.12
My partner is very sensitive to my sexual needs and desires.	.24	.31	.06	**.48**	.09	.19	.01
I eagerly look for signs indicating my partner's desire for me.	-.04	.31	.12	.15	.14	.05	.20
My partner and I share many memories.	.13	.31	-.11	**.42**	-.09	-.06	.24
I often talk about day's events with my partner.	.27	.31	.01	**.50**	.01	-.06	.19
When sacrificing for my partner, I generally do so to make my partner happy.	-.14	.31	-.02	.11	.01	-.01	.28
I am sometimes struck with a sense of awe and wonder when I think about my partner being in my life.	.12	.30	-.07	**.40**	-.04	.08	.12
My partner is the person that I would like to be able to count on to always be there for me and care about me no matter what.	.15	.30	-.20	**.46**	-.17	-.10	.26
When sacrificing for my partner, I generally do so to make my partner feel loved.	-.06	.30	.00	.17	.03	-.03	.26
I'd get jealous if l thought my partner were falling in love with someone else.	-.13	.30	-.15	.17	-.10	.02	.23
My partner is adventurous.	.18	.29	.14	.36	.15	.13	.05
I would forgive my partner for practically anything.	.04	.28	.01	.26	.02	-.01	.20
In our relationship, I often show respect for my partner's feelings about an issue we disagreed on.	.17	.28	-.05	**.42**	-.03	.04	.12
When sacrificing for my partner, I generally do so to create more satisfaction in our relationship.	-.08	.28	.09	.10	.10	.00	.23
I often have tender, concerned feelings for my partner when he/she is less fortunate than me.	-.03	.28	-.12	.24	-.08	.02	.19
I have talked with my partner about things I have done which I am proud of.	.16	.28	.01	.37	.02	-.03	.18
When sacrificing for my partner, I generally do so to increase intimacy in our relationship.	-.02	.27	.25	.08	.24	.02	.20
I would enjoy living apart from my partner.	-.12	-.27	.26	**-.45**	.22	.01	-.16
In our relationship, I often explain my side or suggest a compromise for a disagreement with my partner.	-.05	.27	.05	.14	.07	.04	.18
My partner and I talk about the quality of our relationship often.	.24	.26	.15	.38	.14	.02	.10
My alternatives are attractive to me (dating another, spending time with friends or on my own, etc.).	.06	-.26	.25	-.27	.19	-.07	-.15
I have never regretted my relationship with my partner, not even for a moment.	.24	.26	-.05	**.47**	-.03	.04	.07
In my relationship with my partner, I would describe myself as a pretty soft-hearted person.	-.03	.25	-.11	.23	-.07	.05	.15
I would incur large costs to meet the needs of my partner.	-.05	.25	.03	.14	.06	.05	.16
I have invested a great deal into our relationship that I would lose if the relationship were to end.	-.09	.25	-.12	.16	-.09	-.06	.26
My partner seems disinterested in sex.	-.23	-.25	-.03	-.44	-.06	-.23	.08
I feel very involved in my relationship with my partner - like I have put a great deal into it.	.15	.25	.00	.34	.01	-.05	.18
When I'm upset at my partner, I usually try to "put myself in his/her shoes” for a while.	.07	.24	-.01	.27	.02	.11	.06
My partner and I disagree on sexual matters.	-.20	-.24	.08	-.46	.03	-.24	.08
I feel our sexual activity is just routine.	-.03	-.23	.13	-.31	.07	-.23	.03
In my relationship, I believe that there are two sides to every question and try to look at them both.	.07	.23	-.07	.28	-.04	.02	.13
If I weren't with my dating partner, I would do fine- I'd find another appealing person to date.	.06	-.22	.16	-.19	.12	-.05	-.13
Many aspects of my life have become linked to my partner (recreational activities, etc.) and I would lose all of this if we were to break up.	.00	.22	.02	.15	.03	-.03	.18
When sacrificing for my partner, I generally do so to prevent my partner from feeling let down.	-.11	.21	.12	-.01	.13	-.02	.21
Sometimes I feel I can't control my thoughts; they are obsessively on my partner.	-.20	.21	.18	-.11	.19	.09	.14
I would not feel very upset if my relationship with my partner were to end in the near future.	-.03	-.21	.19	-.29	.15	-.03	-.11
I have talked with my partner about my close relationships with other people.	.06	.20	.02	.21	.03	.03	.11
I try to look at my partner's side of a disagreement before I make a decision.	.06	.20	-.13	.29	-.10	.05	.08
Before criticizing my partner, I try to imagine how I would feel if I were in his/her place.	.05	.19	-.08	.25	-.06	.05	.08
I find it easy to ignore my partner's faults.	.04	.18	-.03	.20	-.01	.03	.09

When I tell my partner about something good that has happened to me, my partner says little, but I know he/she is happy for me.	.01	.12	.09	.06	.08	-.02	.10
My relationships with friends and family members would be complicated if my partner and I were to break up (e.g. my partner is friends with people I care about).	.07	.07	.02	.11	.02	-.05	.07
I like to have power over my partner.	.30	-.14	**.69**	-.17	**.57**	-.17	-.03
I would enjoy having authority over my partner.	.20	-.04	**.68**	-.17	**.58**	-.09	.00
I work to control my partner more than they control me.	.27	-.16	**.65**	-.20	**.53**	-.17	-.04
I try to have more influence than my partner.	.25	-.16	**.63**	-.21	**.51**	-.19	-.02
I have a strong drive to get power in my romantic relationship.	.16	.16	**.60**	-.01	**.53**	-.04	.11
I like to tell my partner what they should do.	.21	-.04	**.58**	-.12	**.49**	-.14	.04
In our relationship, my partner often uses force (like hits, holds me down, or uses a weapon) to make me have sex.	.07	-.08	**.43**	-.20	.37	.01	-.08
In our relationship, I often use force (like hitting, holding down, or using a weapon) to make my partner have sex.	.18	-.13	**.42**	-.12	.35	-.03	-.12
When I tell my partner about something good that has happened to me, my partner reminds me that most good things have their bad aspects as well.	-.13	.05	**.41**	-.28	.37	.04	.05
The people other than my partner with whom I might become involved with are very appealing.	.10	-.18	**.40**	-.25	.32	-.08	-.09
My needs for intimacy and companionship could easily be fulfilled in an alternative relationship.	-.01	-.34	**.40**	-.48	.31	-.13	-.12
In our relationship, I often punch, kick, or beat-up my partner.	.06	-.02	.37	-.13	.32	.03	-.04
In our relationship, I often have a sprain, bruise, or small cut because of a fight with my partner.	-.15	.06	.37	-.26	.34	.12	-.01
In our relationship, I often push, shove, or slap my partner.	.01	-.04	.37	-.20	.32	.02	-.04
In our relationship, my partner often has a sprain, bruise, or small cut because of a fight with me.	.08	-.06	.37	-.15	.31	.01	-.07
It is likely that I will date someone other than my partner within the next year.	-.05	-.11	.36	-.31	.31	.04	-.10
When we have problems, I push, shove, slap, hit, or kick my partner.	.00	-.06	.36	-.21	.31	.05	-.08
In our relationship, I often destroy something belonging to my partner or threaten to hit my partner.	-.03	-.03	.35	-.23	.31	.01	-.01
In our relationship, my partner often punches, kicks, or beats me up.	-.16	.07	.35	-.26	.32	.10	.02
I can readily put the needs of my partner out of my thoughts.	.06	-.17	.34	-.25	.27	-.07	-.08
In our relationship, my partner often destroys something belonging to me or threatens to hit me.	-.30	.08	.33	-.37	.31	.12	.05
When we have problems, I call my partner names, swear at them, or attack their character.	-.27	.11	.32	-.33	.30	.02	.16
During a discussion of a relationship issue or problem, I criticize while my partner defends themself.	.00	-.12	.32	-.27	.26	-.14	.04
When we have problems, my partner pushes, shoves, slaps, hits, or kicks me.	-.31	.16	.31	-.31	.31	.16	.08
My partner and I differ on our general values and beliefs.	-.14	-.08	.31	-.35	.27	.01	-.01
In our relationship, I often insult or swear at my partner.	-.25	.08	.31	-.33	.28	.04	.11
In our relationship, my partner often pushes, shoves, or slaps me.	-.27	.07	.30	-.34	.29	.16	.01
When I tell my partner about something good that has happened to me, he/she points out the potential down sides of the good event.	-.17	.00	.29	-.31	.26	-.01	.07
My partner frequently tries to change my ideas.	-.26	-.01	.28	-.40	.26	.01	.08
It would be easy for me to accept not helping my partner.	-.04	-.17	.28	-.31	.23	.03	-.13
During a discussion of a relationship issue or problem, I pressure, nag, or demand while my partner withdraws, becomes silent, or refuses to discuss further.	-.19	.04	.27	-.29	.24	-.04	.12
I would be reluctant to sacrifice for my partner.	.07	-.17	.27	-.19	.21	-.05	-.10
During a discussion of a relationship issue or problem, my partner tries to start a discussion while I try to avoid a discussion.	-.10	.05	.25	-.19	.23	-.04	.12
When sacrificing for my partner, I generally do so to avoid tension in our relationship.	-.22	.10	.24	-.27	.22	-.04	.19
When I see my partner being treated unfairly, I sometimes don't feel very much pity for him/her.	-.19	-.16	.24	-.42	.21	.05	-.10
When sacrificing for my partner, I generally do so to avoid conflict in our relationship.	-.22	.03	.23	-.31	.21	-.02	.12
I prefer not to show my partner how I feel deep down.	-.12	-.20	.22	-.39	.17	-.06	-.05
My partner's misfortunes do not usually disturb me a great deal.	.03	-.14	.21	-.17	.17	-.01	-.09

When I tell my partner about something good that has happened to me, my partner tries not to make a big deal out of it, but is happy for me.	.03	.03	.19	-.05	.17	-.05	.06
When sacrificing for my partner, I generally do so to prevent my partner from feeling upset.	-.16	.16	.18	-.13	.17	-.01	.18
My alternatives to our relationship are close to ideal (dating another, spending time with friends or on my own, etc.).	.07	-.09	.17	-.08	.14	-.02	-.07
My partner has feelings that are easily hurt.	-.03	-.01	.16	-.13	.13	-.09	.08
When I tell my partner about something good that has happened to me, my partner is usually silently supportive of the good things that occur to me.	.04	.04	.15	-.02	.13	-.06	.07
At times I take my partner for granted.	.00	-.07	.13	-.14	.09	-.17	.10

Note. Item loadings ≥ |.40| on their primary factor are bolded to emphasize representative items.
